# Emergent Global Patterns of Ecosystem Structure and Function from a Mechanistic General Ecosystem Model

**DOI:** 10.1371/journal.pbio.1001841

**Published:** 2014-04-22

**Authors:** Michael B. J. Harfoot, Tim Newbold, Derek P. Tittensor, Stephen Emmott, Jon Hutton, Vassily Lyutsarev, Matthew J. Smith, Jörn P. W. Scharlemann, Drew W. Purves

**Affiliations:** 1 United Nations Environment Programme World Conservation Monitoring Centre, Cambridge, United Kingdom; 2 Computational Science Laboratory, Microsoft Research, Cambridge, United Kingdom; 3 Dalhousie University, Halifax, Nova Scotia, Canada; 4 School of Life Sciences, University of Sussex, Falmer, Brighton, United Kingdom; Centre National de la Recherche Scientifique, France

## Abstract

Anthropogenic activities are causing widespread degradation of ecosystems worldwide, threatening the ecosystem services upon which all human life depends. Improved understanding of this degradation is urgently needed to improve avoidance and mitigation measures. One tool to assist these efforts is predictive models of ecosystem structure and function that are mechanistic: based on fundamental ecological principles. Here we present the first mechanistic General Ecosystem Model (GEM) of ecosystem structure and function that is both global and applies in all terrestrial and marine environments. Functional forms and parameter values were derived from the theoretical and empirical literature where possible. Simulations of the fate of all organisms with body masses between 10 µg and 150,000 kg (a range of 14 orders of magnitude) across the globe led to emergent properties at individual (e.g., growth rate), community (e.g., biomass turnover rates), ecosystem (e.g., trophic pyramids), and macroecological scales (e.g., global patterns of trophic structure) that are in general agreement with current data and theory. These properties emerged from our encoding of the biology of, and interactions among, individual organisms without any direct constraints on the properties themselves. Our results indicate that ecologists have gathered sufficient information to begin to build realistic, global, and mechanistic models of ecosystems, capable of predicting a diverse range of ecosystem properties and their response to human pressures.

## Abstract 2

This paper presents the first mathematical model that attempts to represent the biology and behavior of all individual organisms globally, taking us a step closer to holistic ecological and conservation science founded on mechanistic predictions.

## Author Summary

Ecosystems across the world are being rapidly degraded. This threatens their provision of natural goods and services, upon which all life depends. To be able to reduce—and one day reverse—this damage, we need to be able to predict the effects of human actions on ecosystems. Here, we present the first example of a General Ecosystem Model (GEM)—called the Madingley Model—a novel class of computational model that can be applied to any ecosystem, marine or terrestrial, and can be simulated at any spatial scale from local up to global. It covers almost all organisms in ecosystems, from the smallest to the largest, encoding the underlying biology and behaviour of individual organisms to capture the interactions between them and with the environment, to model the fate of each individual organism, and to make predictions about ecosystem structure and function. Predictions made by the Madingley Model broadly resemble what we observe in real-world ecosystems across scales from individuals through to communities, ecosystems, and the world as a whole. Our results show that ecologists can now begin modelling all nonhuman life on earth, and we suggest that this type of approach may hold promise for predicting the ecological implications of different future trajectories of human activity on our shared planet.

## Introduction

The pace and scale of anthropogenic environmental change has caused the widespread degradation of ecosystems and the services they provide that ultimately support human life on Earth [1]. Understanding and mitigating these impacts necessitates the development of a suite of tools, including policy instruments, practical conservation measures, and empirical research. At present, a variety of models are used to assist decision-making in relation to biodiversity and ecosystem services. Most are correlative, relying on statistical relationships derived from limited observational data without explicit reference to the underlying mechanisms; examples include the GLOBIO model, species distribution models, and models of local extinction based on species–area relationships [2]–[4]. All of these models are useful, for different purposes. However, what is urgently needed is mechanistic models, which explicitly represent the biological, physiological, and ecological mechanisms underlying the systems in question [5]. One of the key benefits of mechanistic models is that they are likely to make more accurate predictions under novel conditions [6]. For example, Earth System Models (ESMs), containing mechanistic descriptions of multiple interacting components of the climate, atmosphere, and ocean, are used to project properties and dynamics under future climate conditions that have not been observed previously (at least in relation to historical data) [7]. Similarly, mechanistic models of ecosystems would allow us to predict a given combination of human pressures on a given ecosystem, even when there is no or little historical data on which to rely. Mechanistic models can also improve our understanding of the systems being modelled, allowing predictions to be understood in relation to the underlying mechanisms that generate them [8]. This in turn might lead to novel ways to mitigate or even reverse the degradation of ecosystems.

We present the first process-based, mechanistic General Ecosystem Model (GEM) (called the Madingley Model). It is general in the sense that it strives to use a unified set of fundamental ecological concepts and processes for any ecosystem to which it is applied, either terrestrial or marine, and it can be simulated at any spatial resolution from local up to global scales. Applying a general modelling approach globally has three main advantages: (1) it allows testing of whether the same set of ecological mechanisms and concepts can adequately capture broad-scale ecosystem behaviour in both the marine and terrestrial realms; (2) it enables the development of a suite of predictive outputs common to both realms, from which standardised metrics of ecosystem health can be calculated; and (3) it enables links between marine and terrestrial ecosystems, both natural and human-driven, to be modelled. The model is also spatially explicit, with dynamics in a given location driven by the climate and other local factors, as well as by connections with other ecosystems through dispersal, and is mechanistic, with dynamics being driven by ecological processes defined at the level of individual organisms. Specifically, we model autotroph (plant) stocks, and individual herbivorous, omnivorous, and carnivorous animals of all body sizes, and their interactions. From these interactions, patterns emerge at larger spatial and temporal scales, including communities, ecosystems, and global macroecological gradients, without any direct model constraints imposed on those properties.

Such a GEM has great potential if it can, at a minimum, reproduce the observed properties of ecosystem structure and function, and enable the formation of valuable, novel hypotheses, and precise, testable predictions. Here, we test the model's ability to simulate ecosystems that persist over long time scales (1,000 y) by comparing model predictions with empirical data and test two theoretical predictions that to date have not been assessed empirically and have only been studied with simple trophic models: that the net primary productivity (NPP) of ecosystems determines the length of trophic chains [9],[10] and that herbivore pressure on autotrophs will reduce once a critical level of carnivore biomass is supported (“trophic release hypothesis” [11]). Finally, we provide a suite of other novel predictions that demonstrate the potential utility of the model as an operational tool with which the effects of human impacts on ecosystems can be explored.

Mechanistic models of specific ecosystems have been developed previously, and to date these have been constrained to particular spatial locations or to particular sets of organisms within ecosystems. For example, dynamic global vegetation models (DGVMs) are used to represent the physiological and ecological processes driving plant community dynamics on the global land surface, enabling investigations into how terrestrial vegetation interacts with climate [12]. However, these do not include animals or other heterotrophs, and so are limited in the extent to which they can be used to understand the roles of heterotrophs in ecosystems, or to address questions about the conservation of organisms other than plants. For the marine realm, “end-to-end” ecosystem models have been developed, which include most trophic levels for particular regions [13]. Examples are the Ecopath With Ecosim (EwE) [14] and Atlantis [15] models. But marine models tend to focus either on biogeochemical cycles or on organisms of economic importance, such as fish, rather than on the properties of the ecosystem as a whole. They also generally either use a stock-and-flow formulation [14],[15], making them unable to follow trajectories of individual organisms, or are restricted to simulating particular guilds of organisms [16].

There have also been previous theoretical examinations of the potential effect of select processes on select ecosystem metrics, such as the role of bioenergetics in determining size distributions [17]. Such theoretical studies have been very useful in providing insight into the potential mechanisms underlying ecosystem structure; but they have tended to be carried out for single, abstract locations that are not tied to any real geographical location and to omit most of the key processes affecting ecosystem structure and function in reality. We are not aware of any previous attempt to model emergent ecosystem structure and function by identifying a minimal, but putatively complete, set of key processes and then simulating these processes for all organisms globally, over the actual climate and geography of all marine and terrestrial environments. It is for these reasons that we refer to the model presented here as a GEM.

## Methods

### Model Scope

**Figure 1:**
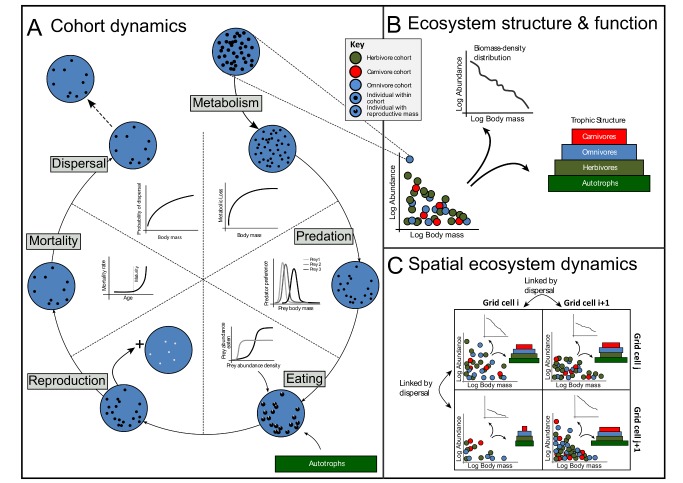
Schematic of the model. Ecosystem structure and function (B) emerges from a combination of processes operating on individual organisms within a grid cell (A), and exchange of individuals among grid cells via dispersal (C). Life histories (e.g., average lifespan, lifetime reproductive success, and generation length) are also emergent (not shown in this diagram, but see Figure 3). Fundamental ecological processes affect the ecological properties (principally body mass and abundance; represented as the diameter and number of black dots in A, respectively) of organisms. For computational efficiency, organisms with similar properties are grouped into cohorts (coloured circles in all panels). Graphs in (A) show illustrative examples of functional forms used to model each ecological process; full mathematical details can be found in the main text and in Text S1. Panel (C) illustrates how dispersal links different grid cells through the exchange of cohorts via dispersal. As result of the within-grid-cell processes, and dispersal, the state of the ecosystem—that is, the collection of cohorts within each grid cell—changes dynamically through time. Panel (B) shows two example measures of ecosystem structure that can be calculated at any time: the relative biomasses in different trophic levels, and the body-mass abundance distribution of heterotrophs. All such community-level properties and metrics emerge from bottom-up processes in the model without any model-imposed constraints beyond those processes operating on individual organisms.

We identified a set of core biological and ecological processes necessary to predict ecosystem-level properties: primary production for autotrophs, and eating, metabolism, growth, reproduction, dispersal, and mortality for heterotrophs (Figure 1 and Box 1). We modelled both marine and terrestrial environments but excluded freshwater ecosystems. We included all photoautotrophs, and all heterotrophs that feed on living organisms (i.e., we did not include chemoautotrophs and detritivores). We generally represented only macroscopic organisms (>1×10^−3^ g), except that we included plankton in the marine realm because of their known importance to the marine food web [18]. All plant biomass in the terrestrial realm was modelled, but only leaves, flowers, fruits, and seeds were available as a food source for herbivores and omnivores. The marine component included phytoplanktonic autotrophs, which provide more than 90% of primary productivity in the oceans [18]. Seagrasses, mangroves, macroalgae, and corals, which are important autotrophs in coastal systems, are not yet included. In this proof-of-concept model, we consider a world without any human impacts, except that we used modern-day climatic conditions. The model and user guide can be downloaded from www.madingleymodel.org, and simulation outputs for main manuscript figures can be downloaded from the Dryad Digital Repository: doi:10.5061/dryad.5m96v [19].

**Box 1. Running simulations within our GEM**

Each simulation is initialised with environmental information from multiple datasets (as described in the Methods section and in Table S1) and spatially distributed stocks and cohorts (see Methods) according to user specifications (functional groups and grid locations). During a simulation, the stocks and cohorts then interact through time and space. For each time step and each grid cell, the first computation is to increment the biomass of autotrophs according to the relevant growth function (Table 5). Next, the ecology of the heterotroph cohorts is modelled (see functions in Table 6). The order in which heterotroph cohorts act is randomised at each time step. Each heterotroph cohort performs multiple ecological processes per time step (Figure 1A): Individuals in the cohort (i) metabolise biomass to sustain their activities; (ii) eat biomass from either autotrophs if herbivorous or other heterotrophs if carnivorous, or from both if omnivorous; (iii) use net biomass gain to grow, if juvenile, or store biomass for future reproduction if they have reached sufficient body mass to be reproductively mature; (iv) give birth to a separate, offspring cohort if sufficient reproductive biomass has been accumulated; (v) suffer mortality as a result of being eaten by other cohorts feeding on them, or as a result of background mortality processes, starvation, or senescence; and (vi) disperse from their current grid cell to another grid cell. As a result of these interactions across space and time, communities of cohorts possessing different functional traits, individual biomasses, and abundances self-assemble (Figure 1B and 1C).

### Traits, Cohorts, and Stocks

Traditional approaches to mechanistic modelling in community ecology focus on densities or abundances of individuals belonging to different species [16],[20]. These are well suited to modelling a small set of focal species, but are unfeasible for modelling whole ecosystems at a global scale, because the vast majority of the world's species remain to be described, or are at best represented by few data describing their distribution and ecology [21]–[23].

Instead, we adopted a trait-based approach [24] where individuals are characterized by their functional traits: categorical traits, such as feeding mode, which determine the mechanisms by which organisms exist and which were used to define functional groups; and continuous traits, such as body mass, which modulate those mechanisms but do not determine functional grouping. Taxonomic identity of individuals is ignored for three reasons. First, there is insufficient species-level information to model whole ecosystems worldwide. Second, it is more feasible to model the role of individuals in ecosystems in terms of their traits than in terms of their taxonomic identity, because of limited taxonomic knowledge and data [21]–[23]. Third, in comparison to taxonomic identity, organisms' functional traits are more directly relevant to most ecosystem functions and ecosystem services [25],[26].

A separate issue is whether to define the model in terms of population densities and biomasses within functional groups (i.e., “stocks” or “pools”), or as collections of interacting individuals each characterized by their combination of functional traits (individual-based). For all organisms except autotrophs, we used an individual-based approach, because this allowed the model to be more finely resolved, and because it enabled us to capture variation in body mass—one of the most important traits for determining the rates of ecological processes [27]–[29] over the lifetime of an individual. It also enabled us to follow the fates of individual organisms. Higher level ecosystem properties emerge from these individual-based rules. Capturing this emergence was a central aim of this initial work.

Autotrophs were represented as *stocks*—that is, the total biomasses of such organisms—because either the definition of an individual was problematic (terrestrial plants) or rates of turnover were faster than the modelled time step (marine phytoplankton).

For heterotrophs, simulating every individual separately would have been computationally intractable given the vast number of individuals in global ecosystems. Therefore, we adopted a computational approach based on *cohorts*. A cohort consisted of a group of organisms occurring within the same grid cell, with identical traits—that is, in the same functional group and with identical continuous traits—but not necessarily belonging to the same taxon. This cohort-based approach allowed us to define the model in exactly the way that one would do in a fully individual-based model (i.e., processes defined at an individual level), but also allowed us to keep the number of computations low enough to be feasible.

### Functional Groups

All stocks and cohorts belong to a functional group, defined according to the categorical traits of the individuals in that stock or cohort (Tables 1 and 2).

**Table 1:** Stock functional group definitions.

Realm	Nutrition Source	Mobility	Leaf Strategy
Marine	Photosynthesis	Planktonic	N/A
Terrestrial	Photosynthesis	Sessile	Deciduous
Terrestrial	Photosynthesis	Sessile	Evergreen

Categorical trait values (column names) with the specific trait values for each stock functional group modelled.

N/A values reflect traits that are not applicable to a functional group.

**Table 2:** Cohort functional group definitions.

Realm	Feeding Mode	Mobility	Reproductive Strategy	Thermoregulation Mode	Log_10_ Minimum Mass for Functional Group (kg)	Log_10_ Maximum Mass for Functional Group (kg)	Herbivory Proportional Assimilation Efficiency	Carnivory Proportional Assimilation Efficiency
Marine	Herbivore (plankton)	Planktonic	Iteroparity	Ectotherm	−8.00	−4.00	0.7	0
Marine	Herbivore (plankton)	Planktonic	Semelparity	Ectotherm	−8.00	−4.00	0.7	0
Marine	Herbivore (plankton)	Mobile	Iteroparity	Ectotherm	−7.00	1.00	0.7	0
Marine	Herbivore (plankton)	Mobile	Semelparity	Ectotherm	−7.00	1.00	0.7	0
Marine	Omnivore	Mobile	Iteroparity	Endotherm	1.00	5.18	0	0.8
Marine	Omnivore	Mobile	Iteroparity	Ectotherm	−8.00	2.00	0.6	0.64
Marine	Omnivore	Mobile	Semelparity	Ectotherm	−8.00	2.00	0.6	0.64
Marine	Carnivore	Mobile	Iteroparity	Endotherm	−1.00	4.70	0	0.8
Marine	Carnivore	Mobile	Iteroparity	Ectotherm	−7.00	3.30	0	0.8
Marine	Carnivore	Mobile	Semelparity	Ectotherm	−7.00	3.30	0	0.8
Terrestrial	Herbivore	Mobile	Iteroparity	Endotherm	−2.82	3.70	0.5	0
Terrestrial	Herbivore	Mobile	Semelparity	Ectotherm	−6.40	0.00	0.5	0
Terrestrial	Herbivore	Mobile	Iteroparity	Ectotherm	−3.00	2.48	0.5	0
Terrestrial	Omnivore	Mobile	Iteroparity	Endotherm	−2.52	3.18	0.4	0.64
Terrestrial	Omnivore	Mobile	Semelparity	Ectotherm	−6.40	0.30	0.4	0.64
Terrestrial	Omnivore	Mobile	Iteroparity	Ectotherm	−2.82	1.74	0.4	0.64
Terrestrial	Carnivore	Mobile	Iteroparity	Endotherm	−2.52	2.85	0	0.8
Terrestrial	Carnivore	Mobile	Semelparity	Ectotherm	−6.10	0.30	0	0.8
Terrestrial	Carnivore	Mobile	Iteroparity	Ectotherm	−2.82	3.30	0	0.8

Categorical trait values (column names) and specific trait values for each cohort functional groups modelled. Organisms with body mass less than 10^−5^ kg are only found in the marine realm and are dispersed planktonically, whereas all organisms with body mass greater than 10^−5^ kg moved through mobile dispersal.

Individuals in the same functional group interact with one another and with their environment in a qualitatively similar manner. Therefore, cohorts within the same functional group are modelled using the same mathematical representations of the ecological processes, though the rates predicted by those functions differ according to continuous traits that differ between cohorts, such as body mass. Individuals belonging to different functional groups have at least one qualitatively different interaction with other individuals or with their environment. We use the same functional forms for analogous functional groups in the ocean and on land, but with different parameter values, where justified by previous research.

Body mass affects many individual properties and interactions, including feeding preferences and rates, metabolic rates, and dispersal [27]–[29]. Therefore, body mass was included as a parameter in nearly all ecological processes of heterotrophs (Figure 1).

### The Environment

The environment is defined as a two-dimensional layer representing the land surface and the upper mixed layer (top 100 m) of the oceans. This layer is divided into grid cells within which individuals and stocks are assumed to be well mixed. The ecological processes can be affected by the size of the grid cell, the physical environment at that cell, and dispersal of organisms among adjacent grid cells. The model can be employed for any number of grid cells, at any resolution, locally or globally, subject to computational limitations. For the results presented here, we used either simulations for individual 1°×1° grid cells, or a 2°×2° grid covering the whole globe (see below, and Tables 3 and 4) except for high latitudes (>65°) because remotely sensed, exogenous environmental data currently used are not available for the polar regions. Each grid cell in the model is assigned to either the terrestrial or marine realm based on a land/ocean mask [30]. Environmental conditions for each grid cell are read as model inputs from publicly available datasets: for the terrestrial realm air temperature [31], precipitation [31], soil water availability [32], number of frost days [31], and seasonality of primary productivity [33]; and for the marine realm sea-surface temperature [34], NPP [35], and ocean current velocity (Table S1) [34]. The model is flexible with respect to the specific environmental data used, and future simulated environmental conditions can be used.

**Table 3:** Settings used for the six model studies conducted in this research article.

Study	Description	Spatial Extent	Length (y)	Ensemble Number	Output Detail
1	Grid-cell numerical analyses	Four 1°×1° focal grid cells (Table 4)	1,000	10	Biomass and abundance densities by functional groups
2	Grid-cell individual- and community-level predictions	Four 1°×1° focal grid cells (Table 4)	100	1	Detailed individual-level process diagnostics
3	Grid-cell community-level predictions at empirically observed locations	Fourteen 1°×1° grid cells at locations where ecosystem structure has been empirically estimated (Table S3)	100	10	Biomass and abundance densities by functional group
4	Global predictions	Global grid of 2°×2° cells extending from 65°N to 65°S in latitude and from 180°E to 180°W in longitude	100	1	Biomass and abundance densities by functional group
5	Effects of dispersal on marine trophic structure	Global grid of 2°×2° cells in the marine realm only extending from 65°N to 65°S in latitude and from 180°E to 180°W in longitude	100	1	Biomass and abundance densities by functional group
6	Effects of biomass turnover rates on marine trophic structure	Grid cell M1 (Table 4)	100	10	Biomass and abundance densities by functional group for simulations with differing biomass turnover rates.

**Table 4:** Descriptions and coordinates of the focal grid cells for which detailed numerical-, individual-, and community-level model simulations were made.

Cell Number	Cell Description	Latitude	Longitude	Location
T1	Terrestrial∶tropical, aseasonal	0°N	32.5°E	Southern Uganda
T2	Terrestrial∶temperate, seasonal	52.5°N	0.5°E	Central England
M1	Marine: low productivity, aseasonal	−25.5°S	−119.5°W	South Pacific Ocean
M2	Marine: high productivity, seasonal	42.5°N	−45.5°W	North Atlantic Ocean

### The Ecology

We provide a summary of how simulations are run in Figure 1 and Box 1, and an overview of how the ecological processes are modelled, with the main mathematical functions, is summarised in Tables 5 and 6. Full details are provided in Text S1.

**Table 5:** Summary of how autotroph ecological processes are modelled (for full details, see Text S1 and Table S2).

Process	Realm	Main Mathematical Functions	Eqn(s).	Assumptions
Growth	Marine	The growth of phytoplankton, *p*, biomass in marine cell, *M*, during month, *m*, is given by: 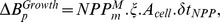 where 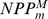 is a remotely sensed estimate of monthly marine NPP; *ξ* converts from carbon to wet matter biomass; *A_cell_* is the grid cell area; *δt_NPP_* converts from monthly values to the model time step.	3 in Text S1	The modelled standing biomass of phytoplankton is capable of generating the remotely sensed productivity in any given time step
Growth	Terr.	The growth of biomass in autotroph stock, *l*, in terrestrial cell, *T*, during month, *m*, is given by: 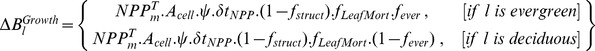 where 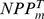 is a remotely sensed estimate of monthly terrestrial NPP; *A_cell_* is the grid cell area; *ψ* converts from carbon to wet matter biomass; *δt_NPP_* converts from monthly values to the model time step; *f_struct_* is the fractional allocation of primary production to structural tissue; *f_ever_* is the proportion of NPP produced by evergreen leaves at a particular location; and *f_LeafMort_* is the proportion of total mortality that is leaf mortality.	5, 7–19 in Text S1	Annual mean environmentally determined NPP is allocated to months in the same relative proportions as that observed in remotely sensed NPP data.
Mortality	Marine	The loss of phytoplankton biomass is given by: 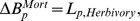 where 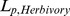 is the cumulative phytoplankton biomass consumed through herbivory.	4 in Text S1	Background mortality of phytoplankton is negligible compared to losses from herbivory
Mortality	Terr.	The loss of biomass from autotroph stock, *l*, is given by: 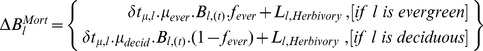 where 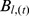 is the biomass of stock l at time *t*; *μ_ever_* and *μ_decid_* are annual leaf mortality rates for evergreen and deciduous stocks, respectively; 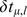 converts annual leaf mortality rates to the model time step; 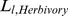 is the cumulative biomass of stock, *l*, consumed through herbivory; and *f_ever_* is the proportion of NPP produced by evergreen leaves at a particular location.	6, 10–15 in Text S1	Herbivory rates do not affect plant allocation strategies and plants do not have the capacity for defensive strategies

**Table 6:** Summary of how heterotroph ecological processes are modelled.

Process	Main Mathematical Functions	Eqn(s).	Assumptions
Herbiv.	The total biomass assimilated as food by cohort *i* through herbivory on all stocks is calculated as follows: 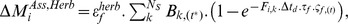 where 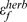 is the fractional herbivore assimilation efficiency for the functional group, *f*, to which cohort *i* belongs; 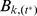 is the biomass of stock *k* at time t^*^ when herbivore cohort *i* acts; Δ*t_d_* is the length of the model time step in days; *τ_f_* is the proportion of the time step for which functional group *f* is typically active; 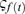 is the proportion of the time step that is suitable for a cohort of functional group *f* to be active; and 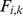 the instantaneous rate at which stock *k* is eaten by an individual from herbivore cohort *i* is determined by: 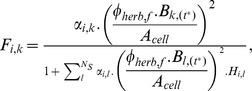 where 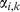 is the effective rate at which an individual herbivore searches its environment in hectares per day, and which is assumed to scale linearly with herbivore body mass; 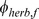 is the proportion of the current biomass of stock *k* that is experienced by cohort *i*; 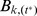 is the biomass of stock *k* at time t^*^ herbivore cohort *i* acts; *A_cell_* is the area of the cell; 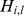 is the time taken for an individual in cohort *i* to handle a unit mass of autotroph stock *l*.	S23, S24, S26, S30–S32	Autotroph biomass and herbivore cohorts are well mixed throughout each cell.Each herbivore cohort encounters a separate fraction, 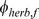 , of the total autotroph biomass available.
Predation	The total biomass assimilated as food by cohort *i* through predation on all cohorts is calculated as follows: 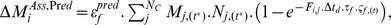 where 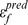 is the fractional herbivore assimilation efficiency for the functional group, *f*, to which cohort *i* belongs; 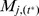 is the body mass of an individual of cohort *j* at time t^*^ when predator cohort *i* acts; 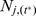 is the abundance of individuals in cohort *j* at time t^*^; and 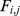 , the instantaneous rate at which a prey cohort *j* is eaten by an individual predator from cohort *i*, is determined by: 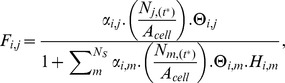 where 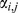 is the effective rate at which an individual predator searches its environment and successfully kills prey; 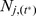 is the abundance of cohort *i* at time t^*^ when predator cohort *i* acts; 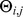 is the cumulative density of organisms with a body mass lying within the same predator-specific mass bin as cohort *j*; 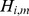 is the time taken for an individual in cohort *i* to handle one individual prey individual in cohort *m*, per unit time spent searching for food.	S23, S25, S28, S34–S39	Predator and prey cohorts are well mixed throughout each cell.Predator cohorts can experience all other cohorts sharing the same cell.While searching for one prey, predators can be simultaneously encountering another prey—that is, they are not limited by search time.
Omniv.	The total biomass assimilated as food by an omnivore cohort is the sum of the assimilation terms for herbivory and predation as described above but with 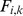 , the instantaneous rate at which stock *k* is eaten by an individual from an omnivorous cohort *i* determined by: 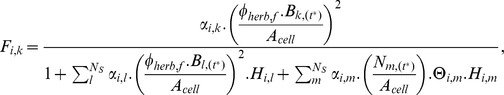 and 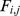 , the instantaneous rate at which a prey cohort *j* is eaten by an individual omnivore from cohort *i*, given by: 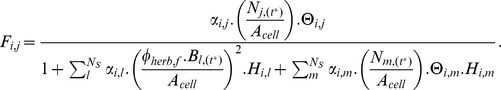 Where variables and parameters are as described for herbivory and predation above.	S23–S25, S27, S29, S30–S32, S34–S39	As described above for herbivory and predation.Omnivores spend a fixed fraction of each time step engaged in each of herbivory and predation.
Metab.	The metabolic loss of biomass from each individual of cohort *i*, each with body mass *M_i,(t)_*, was modelled as follows: 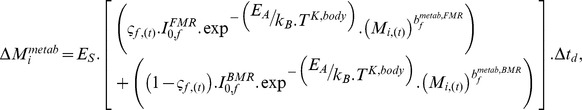 where 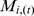 is the body mass of an individual in cohort *i*; *E_S_* converts from energy to biomass; 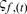 ; 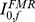 and 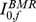 are mass- and temperature-independent metabolic rate constants for field and basal metabolic rates, respectively; *E_A_* is aggregate activation energy of metabolic reactions; *k_B_* is the Boltzmann constant; *T^K,body^* is the body temperature of the individual; 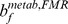 and 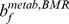 are body mass exponents for field and basal metabolic rates, respectively.	S48	Body temperature, *T^K,body^*, is assumed to be 310 K for endothermic organisms and equal to ambient temperature for ectothermic functional groups.
Reprodn.	The biomass allocated to reproduction for cohort *i* is modelled as: 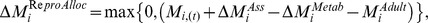 where 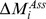 is the total biomass assimilated as food; 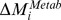 is mass lost through metabolism; 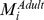 is the body mass at which an individual of cohort *i* reaches reproductive maturity.A reproductive event was assumed to occur when the following threshold condition was met: 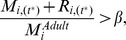 where 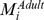 is the mass at which an individual of cohort *i* reaches reproductive maturity; 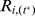 is the stored reproductive potential biomass of each individual in cohort *i* at the current time; *β* is the threshold value for accumulation of reproductive potential biomass.	S49–S54	Semelparous organisms can allocate a fraction of their adult mass to reproductive events.
Mortality	The instantaneous rate of senescence mortality was modelled as: 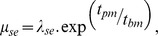 where *λ_se_* is the instantaneous rate of senescence mortality for a cohort at the point of maturity; *t_pm_* is the time that it took for the cohort to reach maturity; *t_bm_* is the time since the cohort reached maturity.The instantaneous rate of starvation mortality is given by: 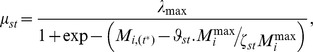 where *λ*_max_ is the maximum possible instantaneous fractional starvation mortality rate; 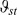 determines the inflection point of the logistic function describing the ratio of the realised mortality rate to the maximum rate; *ζ_st_* is the scaling parameter for the logistic function describing the ratio of realised mortality rate to the maximum rate; 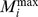 is the maximum body mass ever achieved by individuals in cohort *i*.The instantaneous rate of background mortality, *μ_bg_*, was modelled as a constant value.	S55–S58	There is no senescence mortality applied to cohorts that have not reached maturity.
Dispersal	Three types of dispersal were included in the model, two of which—diffusive natal dispersal and responsive dispersal—applied across all realms, whereas advective dispersal applied in the marine realm and to planktonic size organisms only.Diffusive natal dispersal modelled the characteristic dispersal distance of each cohort as a function of body mass as follows: 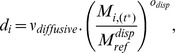 where 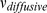 is the dispersal speed of an individual of body mass equal to the dispersal reference mass 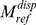 ; *o_disp_* dispersal distance body mass exponent. Active dispersal in adults is attempted if intracohort density of adult individuals is below a mass-related density threshold: 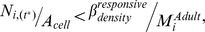 or if the proportion of body mass lost in a time step exceeds a starvation threshold: 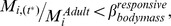 where the betas represent those thresholds.The final, advectively driven dispersal is applicable solely to planktonic organisms in the marine realm and modelled using the two-dimensional advective vector at that time step and location, with an additional diffusive component of random direction and length.	S59–S61	Cohorts are spread homogeneously across grid cells.Cohorts disperse in entirety not as fractions.The diffusive dispersal of immature organisms is assumed to represent them searching for new territory.

Note that the same processes apply in the terrestrial and marine realms. For full details, see Text S1 and Table S2.

#### Autotroph Ecology

In the marine realm, one stock of phytoplankton per grid cell is modelled, characterised by a total wet matter biomass at time *t*, 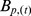. For terrestrial autotrophs, we track two stocks of leaves, *l*, one deciduous and one evergreen, each characterized by a total biomass at time *t*, 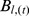. The biomass of an autotroph stock *s*, which could be either phytoplankton (*p*) or leaves (*l*), is incremented in each time step (of length Δ*t*) as follows:
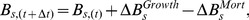
(1)where 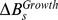 and 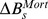 are the gain and loss of biomass from stock *s* over the time interval Δ*t*, respectively. 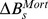 includes losses due to herbivory. We use different approaches to model the gain and loss of biomass from marine and terrestrial autotroph stocks (Table 5).

In the marine realm, we model growth of the phytoplankton stock by incrementing the total biomass of phytoplankton in each cell using satellite-derived estimates of NPP (Table 5). This avoids us having to adopt computationally impractical time-steps (days) in order to accurately simulate the dynamics of phytoplankton, which have rapid turnover rates (in our opinion, an explicit nutrient–phytoplankton–zooplankton model would be a major improvement to the marine part of the model; see Table 7). Loss of phytoplankton arises directly from modelling grazing by marine organisms (Table 5; Equations 3 and 4 in Text S1).

**Table 7:** The major development needs for the Madingley Model organised by development category.

Category	Development Need
**Data**	1. Source individual organism-level data with which to constrain ecological processes such as mortality from disease and environmental disturbance, reproductive behaviour, dispersal behaviour, and activity rates in response to environmental or food stress.2. Gather information on local community structure to evaluate community-level predictions, such as total biomass of functional or trophic groups, whole communities, individual size distributions for entire communities in the terrestrial realm, and biomass fluxes through ecosystems.3. Collect data detailing ecosystems across space and through time to evaluate emergent ecosystem-level properties—for example, latitudinal or longitudinal transects of biomass and/or abundance, total biomass and/or abundance within a region, and changes in ecosystem structure in response to environmental change through space and/or time.4. Assemble data on quantified interaction networks for communities with which to compare the individual-level interaction networks predicted by the model.
**Ecology**	1. Include detritivores as a functional group, including the ecological processes that link detritivores to the organic matter inputs from the ecosystem and cycle that processed organic matter back to form inputs for the primary producers of the system, as well as representing detritivore-based food chains.2. Resolve sub-grid-cell habitat structure and organismal preferences within that structure that will likely lead to patterns of interactions among organisms that violate the well-mixed assumption, for example, by providing refugia for prey: in forests for instance, canopy-dwelling predators encounter canopy-dwelling prey more often than expected, but ground-dwelling prey less often than expected.3. Incorporate an explicitly resolved, mechanistic model of phytoplankton dynamics with two-way linking between phytoplankton and zooplankton.4. Incorporate a three-dimensional spatial structure in the oceans with a temporally and spatially varying mixed-layer and deep chlorophyll maxima5. Represent nonphytoplanktonic coastal NPP (seagrass beds or coral reefs) in order to more realistically capture biodiverse and productive coastal ecosystems.6. Capture varying organismal ecological stoichiometry to account for biochemical limitations on performance and also to being able to explore biogeochemical cycling through entire ecosystems; useful starting points include ecological stoichiometric [112] and dynamic energy budget [113] concepts.7. Simulate freshwater ecosystems in addition to terrestrial and marine, and source necessary datasets to constrain ecological parameters and evaluate outputs.8. Introduce the concept of intelligent behaviour—for example, directed dispersal (e.g., along a gradient of resources), hibernation and stasis strategies, or complex predator–prey and herbivore–plant interactions
**Methods**	1. How does underlying behaviour feed up to mathematical representations of ecological functions? For example, how does the intelligent behaviour of predators and prey affect the Hollings' functions employed to model this interaction?2. Analysis of the model framework to identify analytical solutions for emergent properties such as size distributions or trophic structure.3. Study the implications of the numerical methods employed in the model such as the time step of ecological processes and the cohort approximation.4. Implement a variable time-scale method wherein smaller, more metabolically rapid organisms have a faster time step [14], which may stabilise the marine realm and will be needed to implement a fully coupled dynamic and mechanistic phytoplankton model.5. Formally constrain the model against data (data needs are described above) in order to rigorously select the most appropriate assumptions, functional forms, and parameter values.

Terrestrial autotrophs are modelled using the climate-driven terrestrial carbon model of Smith et al. [36]. We selected this model because it has been parameterized and tested against empirical data on carbon stocks and flows more rigorously than similar models of which we are aware (Equations 5–19 in Text S1) [36]. Moreover, it has a similar level of complexity to that used to represent heterotrophic organisms. However, like the other model components we adopted, the vegetation model could be replaced by alternatives in future studies, including more complex models able to address particular issues like CO_2_ fertilization (e.g., [12],[37]). Terrestrial plant growth is modelled as a function of NPP, the proportion of NPP that is produced by evergreen or deciduous leaves, and the fraction of NPP allocated to structural tissues (Table 5), all of which depend on the local climate. The loss of plant biomass is determined by leaf mortality rate, which is also function of the climate, as well as the consumption of biomass by herbivorous terrestrial organisms (Table 5).

#### Heterotroph Ecology

Heterotrophs are modelled as cohorts. Each cohort *i* is characterized by a functional group (Table 2), by two traits that do not change through time—body mass at birth, 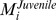, and body mass at reproductive maturity 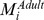 (for cohort, *i*)—and by three state variables whose values do change through time—the abundance of individuals *N_i,(t)_*, the wet matter body mass of each individual within the cohort *M_i,(t)_*, and a stored reproductive mass of each individual, *R_i,(t)_* (Figure 1). The values of these state variables are updated each time step according to the effects of ecological processes (Table 6). Individuals in each cohort are assumed to interact only with stocks and cohorts in the same grid cell.

The growth of individual body mass is modelled as follows:
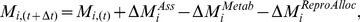
(2)where 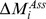 is the total biomass assimilated as food, which is the sum of the biomasses assimilated through herbivory and predation, 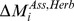 and 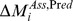, respectively; 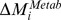 is mass lost through metabolism; and 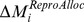 is mass lost by allocation to reproduction (Table 6, see also Figure 1).

Predation and herbivory are modelled using a Holling's Type III functional response, which assumes that the number (or biomass) of prey (or plant material) eaten by an individual predator (or herbivore) is a sigmoidal function of prey density (or biomass density) (Table 6) [38]. The Holling's functions require definition of the attack rate and handling time for each predator (or herbivore) cohort on each prey cohort (or plant stock). Attack rates of herbivores on plants scaled according to the body mass of herbivore. Attack rates of predators on animals were derived from the size-structured model of Williams et al. [39], where the probability of predation is a Gaussian function around an optimal prey body size (as a proportion of predator size) (see Equations 35 and 36 in Text S1) estimated from large empirical datasets on feeding relationships [40]. This size-structured model is an extension of the long-standing niche model [41] but could be replaced with other predator–prey interaction models in future studies if desired. For carnivores and omnivores, the handling time of each predator on each prey increases linearly with prey body mass (larger prey take longer to eat) but decreases as defined by a power-law relationship with predator body mass (larger predators handle prey more quickly) (Equation 40 in Text S1). For herbivores, handling time depends on herbivore body mass only (a decreasing power-law relationship) (Equation 32 in Text S1).

Metabolic costs are modelled as a power-law relationship with body mass, following Brown [29], using parameter values derived from field metabolic rates (Table 6) [42]. We assume that each cohort is active for some proportion of each time step according to ambient temperature (Equations 41–47 in Text S1). Endotherms are assumed to thermoregulate, and thus are active for 100% of each time step. Marine ectotherms are active for 100% of each time step. Terrestrial ectotherms do not thermoregulate, and thus are only active for the proportion of each time step during which ambient temperature was within their upper and lower activity temperature limits, estimated following Deutsch et al. [43].

Once an individual reaches its adult mass, we assume that all further mass gained is stored as reproductive potential. An individual's reproductive potential mass is incremented as follows:
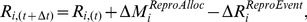
(3)where 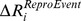 is the potential reproductive biomass lost by each individual of cohort *i* through reproductive events (Figure 1). Reproductive events occur when an individuals' stored reproductive potential reaches a threshold proportion of adult mass (Table 6). During reproductive events, iteroparous organisms devote all of their stored reproductive potential mass to producing offspring; semelparous organisms devote all of their stored reproductive potential mass, and also a proportion (Equations 50–52 in Text S1, Table S2) of their adult mass.

The number of individuals in each cohort is incremented as follows:
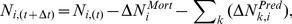
(4)where 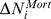 is the number of individuals of cohort *i* lost to nonpredation mortality, and 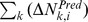 is the total number of individuals of cohort *i* lost through predation, summed over all predator cohorts *k* as outlined above (Figure 1). We model three sources of nonpredation mortality: a constant proportional rate of background mortality, which applies to all individuals; starvation mortality, which is applied according to how much body mass has been lost compared to the maximum body mass ever obtained by an individual; and senescence mortality, which increases exponentially after maturity with a functional form similar to the Gompertz model (e.g., [44],[45]) (Table 6). Note that abundance only ever decreases within a cohort. New individuals generated through reproduction produced new offspring cohorts (see below) (Equations 52–54 in Text S1). For computational efficiency, once the number of cohorts exceeds a user-specified, computationally tractable threshold, a number of pairs of cohorts equal to the excess are merged together. On merging, the biomass of one of the cohort pair is converted into an equivalent number of individuals of the other cohort in the pair (Equations 68–69 in Text S1). The cohort pairs identified for merging are those lying closest together in continuous trait space, and belonging to the same functional group (Equation 67 in Text S1).

Individuals were exchanged among the grid cell via three types of dispersal: (1) random diffusive dispersal of newly produced (juvenile) cohorts, (2) active dispersal of individuals determined by the degree of starvation experienced, and (3) advective-diffusive dispersal driven by ocean currents (in the marine realm only) (Table 6). Dispersal occurred via the movement of whole cohorts, such that cohorts remained intact. This was necessary numerically to keep the number of cohorts manageable. We carried out some targeted simulations to explore the effects of allowing cohorts to split on dispersal, and found that could have quantitative effects, but does not fundamentally alter dynamics (Figure S1). Assumptions and functional forms about dispersal, and numerical schemes to implement them, are another potentially important area for future research.

When the model was applied to a specific grid cell in isolation, dispersal into or out of the grid cell was not modelled.

### Emergent Properties

The properties of individuals and communities that we present below are “emergent”; that is, they are not prescribed, but instead emerge through time as a result of the large number of interactions that take place between individual organisms (approximated as cohorts). As a result of these interactions, life histories of individuals are formed over time and can be tracked, and communities and ecosystems of individuals self-assemble. Moreover, the dynamics of any one grid cell are affected by the exchange of individuals with other grid cells, which occurs due to dispersal. Thus macroscale predictions (e.g., over the generation length of an individual cohort, across functional groups, or across entire ecosystems) emerge from microscale biological mechanisms. The macroscale predictions differ for ecosystems in different climates, but only because the microscale biology is sensitive to the climate. Similarly, the macroscale predictions differ between the land and sea, but only because microscale biology differs between land and sea. We compared these emergent properties to empirical data.

### Model Simulations and Comparison to Data

We carried out four distinct types of simulations for different assessments of model capabilities (Table 3).

Terrestrial grid cells were seeded with two autotroph stocks, deciduous and evergreen, as detailed above, and marine grid cells were seeded with a single phytoplankton stock. Grid cells were seeded with around 1,000 cohorts each, with 112 cohorts in each of nine functional groups in the terrestrial realm and 100 cohorts in each of 10 functional groups in the marine realm. Juvenile and adult body masses of cohorts were drawn at random from a prespecified range (Table 2), and initial abundance was scaled negatively with initial body mass to provide reasonable initial densities (see Text S1 for full details).

Detailed numerical analyses were conducted on four focal grid cells (Study 1, Table 3) to investigate ecosystem dynamics over longer time scales. These simulations were used to check for the persistence of key community components (autotrophs, herbivores, carnivores, and omnivores), and to determine the typical time scales for the dynamics to reach some form of equilibrium. These analyses used the same climatological time series per year to remove the effects of interannual environmental variation. For each focal grid cell, we ran 10 model simulations for 1,000 y at a monthly time step. To test the effect of the cohort-merging regime on modelled dynamics, we repeated the simulation ensembles for each focal grid cell with the threshold number of cohorts at which merging is activated set at 500, 1,000, 5,000, and 10,000 cohorts and for a shorter period of 100 y.

Additional detailed simulations were carried out for the focal grid cells over a 100-y period to generate highly resolved predictions of emergent ecosystem properties at two levels of biological organisation: individual and ecosystem level (Study 2, Table 3).

We compared the properties of individual organisms with empirical data. Importantly, none of these properties were defined in the model as parameters, but rather they emerged as a result of the ecological interactions among individuals. The predicted relationship between body mass and growth rates was compared to estimates for reptiles, mammals, birds, and fish [46]–[48], and the relationship between body mass and time to reach maturity to estimates for invertebrates, reptiles, mammals, birds, and fish [49]–[57] (where necessary body masses were estimated from body lengths using relationships in [58]–[61]). The predicted relationship between body mass and mortality rates was compared with data for invertebrates, mammals, birds, and fish, taken from a single study [62]. The predicted relationship between body mass and lifetime reproductive success was compared with data for mammals, birds, and a few insect species [63]–[71]. Of these emergent properties, the growth rate of organisms derives most closely from the functional forms and input parameters. Specifically, growth rate could theoretically be a simple sum of food assimilation rates under conditions of saturating prey density minus metabolic costs. To test whether this was the case, we calculated the theoretical growth rate for organisms of a range of body masses under these conditions.

We compared our novel predictions of complete ecosystem structure in two grid cells (T1 and M1) (Table 4) to empirical data: the biomass density of large herbivores with an estimate for Uganda [72], and the predicted herbivore to autotroph biomass ratios with average observed ratios for similar ecosystems [73]. We further compared the modelled relationships between body mass and population density with empirical estimates derived from fish assemblages [74],[75].

To test the ability of our model to capture broad-scale patterns in the basic trophic structure of ecosystems, we compared our model predictions (Study 3, Table 3) to empirical estimates from the same dataset used to calculate the global average trophic structure ([73]; see above), but this time using specific values for 14 sites for which we could identify the spatial location (Table S3). This dataset is the most geographically wide-ranging dataset on ecosystem structure that we are aware of, including sites in both the terrestrial and marine realms.

We also generated model outputs at a global scale (Study 4, Table 3). These were used in two ways: firstly, to investigate the mechanisms giving rise to variation in ecosystem structure by assessing the relationship between trophic structure and productivity in the model along large gradients of autotroph productivity in both terrestrial (along a meridional transect from the low productivity Saharan desert to high productivity Congo Basin tropical forest region) and marine (along a meridional transect from low productivity Antarctic waters to high productivity East Atlantic upwelling zones) realms; and secondly, to make novel predictions of as-yet unmeasured global properties (e.g., latitudinal gradients in total biomass) and to compare to other modelled estimates of total biomass and density at a global scale [76]. We also compared modelled global average ratios of herbivore to autotroph biomass with the average ratios observed in the same dataset that we used to test the predictions made by individual cells [73]. Finally, we investigated how modelled marine ecosystem structure responded to mechanisms that have been proposed to cause inverted trophic biomass pyramids, such as dispersal [77], turnover rate of autotrophs, and turnover rate of consumers [77]–[79]. To do this, we simulated the global marine realm with no dispersal permitted between grid cells (Study 5, Table 3) and the effects of reducing the turnover rate of biomass through the ecosystem (Study 6, Table 3).

## Results

### Grid Cell: Dynamics

**Figure 2:**
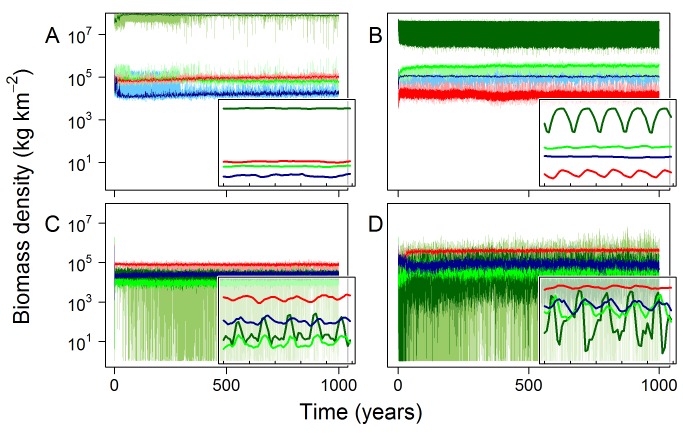
1,000-year dynamics for four locations. Medians from ensembles of 10 replicate simulations (lines) and absolute ranges (shaded regions) of biomass densities for autotrophs (dark green lines), herbivores (light green), omnivores (blue), and carnivores (red) within four 1°×1° focal grid cells; T1, terrestrial aseasonal (A); T2, terrestrial seasonal (B); M1, marine aseasonal (C); and M2, marine seasonal (D) (Table 4). The temporal dynamics in these metrics emerges from underlying ecological processes that affect a large number of cohorts within each grid cell. Insets zoom in on medians for the last 5 y of the simulations, demonstrating the seasonal variability in each cell.

For the 1,000-y simulations (Study 1), model dynamics converged rapidly (<100 y) to dynamic equilibria for all 10 replicates in all four focal grid cells (Figure 2). Autotrophs, herbivores, omnivores, and carnivores persisted in all simulations. The dynamics of total biomass by functional group differed markedly among the four grid cells. Terrestrial grid cells were dominated by autotroph biomass and, among heterotrophic organisms, by herbivores, with lower biomasses of omnivores and carnivores. Marine grid cells, in comparison, had much lower biomasses of autotrophs, and omnivorous and carnivorous organisms were more dominant. Unsurprisingly, the seasonal grid cells in both the terrestrial and marine realms (Figure 2B and 2D) exhibited much greater fluctuations in biomasses within years, particularly for lower trophic levels. The high-productivity marine grid cell exhibited large-amplitude, high-frequency variations in zooplankton abundance. Biomass dynamics were robust to the choice of the threshold number of cohorts at which to activate merging above a threshold of 1,000 cohorts (Figure S2).

### Grid Cell: Individuals

**Figure 3:**
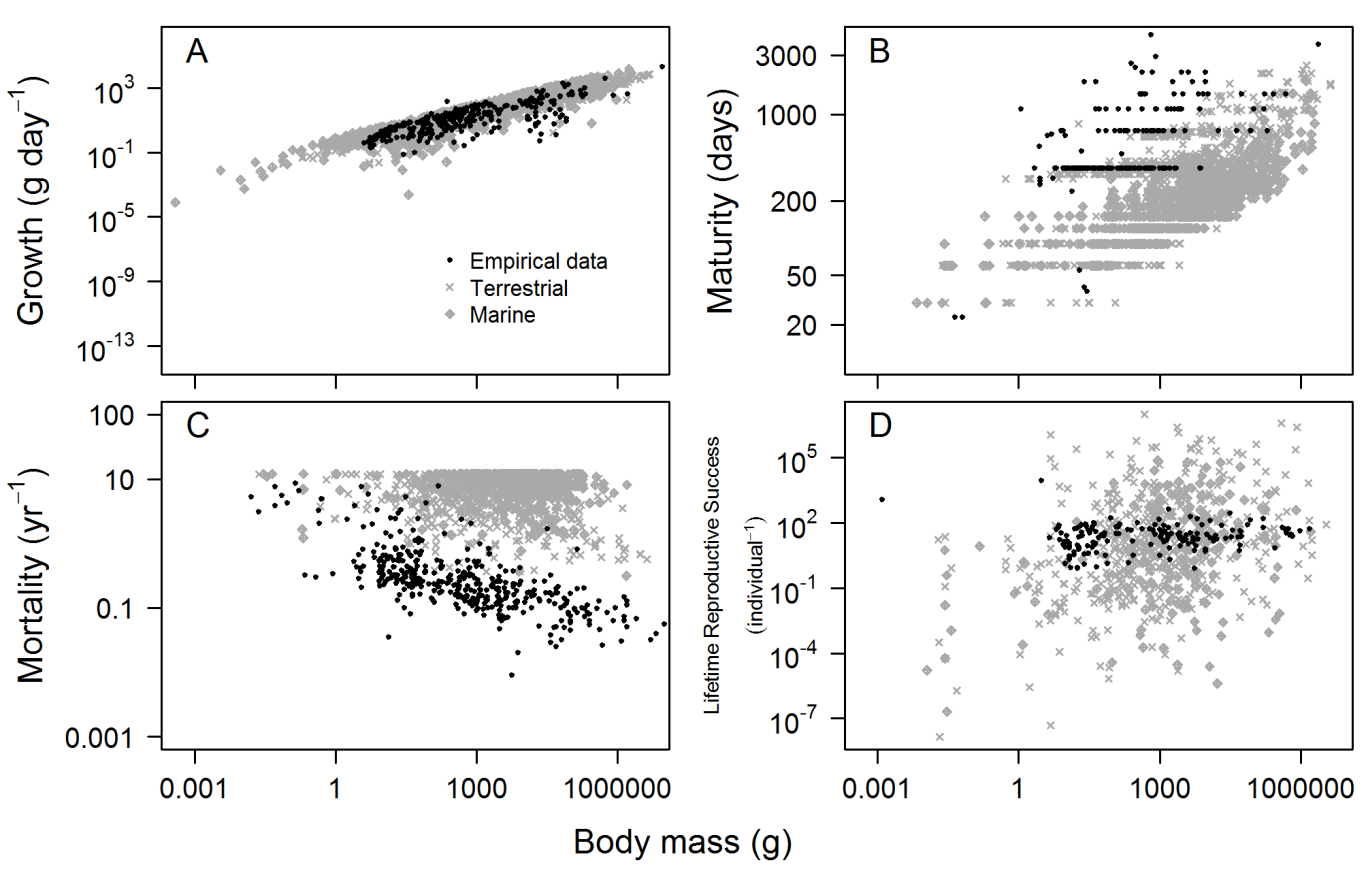
Comparison of emergent life history metrics with empirical data. Empirical (black) and emergent model (grey) relationships between body mass and (A) growth rate, (B) maturity, (C) individual mortality rates, and (D) lifetime reproductive success. These life history metrics are not part of the model definition. Rather, they emerge from underlying ecological processes such as metabolism and feeding (see main text). Life history metrics were sampled from 100-y model runs for the four focal grid cells (Table 4). Individual mortality rates are estimated as the inverse of lifespan, and because the minimum simulated lifespan is one model time step (1 mo), estimated individual mortality rates were bounded at 12.

The power-law relationships between body mass and the properties of individual organisms that emerged from the model were generally consistent with empirical data (Figure 3). For growth rates and times taken to reach reproductive maturity, the modelled and empirical values were very similar, although the slope of the relationship between predicted growth rates and body mass was steeper. In absolute terms the predicted growth rates tended to be higher and the predicted times to maturity tended to be lower than those observed in the empirical data (Table S4).

Our assumptions about underlying ecological processes, such as handling times and metabolic rates, place a fundamental limit on the growth rate of organisms of a given body mass (i.e., the net growth rate of individuals that are feeding at the maximum rate). If most individuals attained this maximum, then the growth rates would not be emergent, so much as defined by the model assumptions. But this was not the case. The emergent relationship between body mass and growth rate was not a simple function of maximum possible food assimilation and metabolic costs: modelled growth rates were typically one-tenth of theoretical maximum model growth rates and showed large variation for any given body mass (Figure S3). This variation resulted from the many other factors that affected growth rate, most notably the abundance and body masses of potential prey and predators competing for the same prey.

Predicted mortality rates showed a negative power-law relationship with body mass (Figure 3C), qualitatively consistent with empirical data, although the relationship was generally shallower and absolute rates were higher (Table S4). Finally, predicted reproductive rates in the terrestrial realm showed a weak positive power-law relationship with body mass, broadly consistent with empirical estimates (Figure 3D, Table S4), whereas predicted reproductive rates in the marine realm were weakly negative. Predicted reproductive rates were substantially more variable than the empirical data (Figure 3D).

### Grid Cell: Community

**Figure 4:**
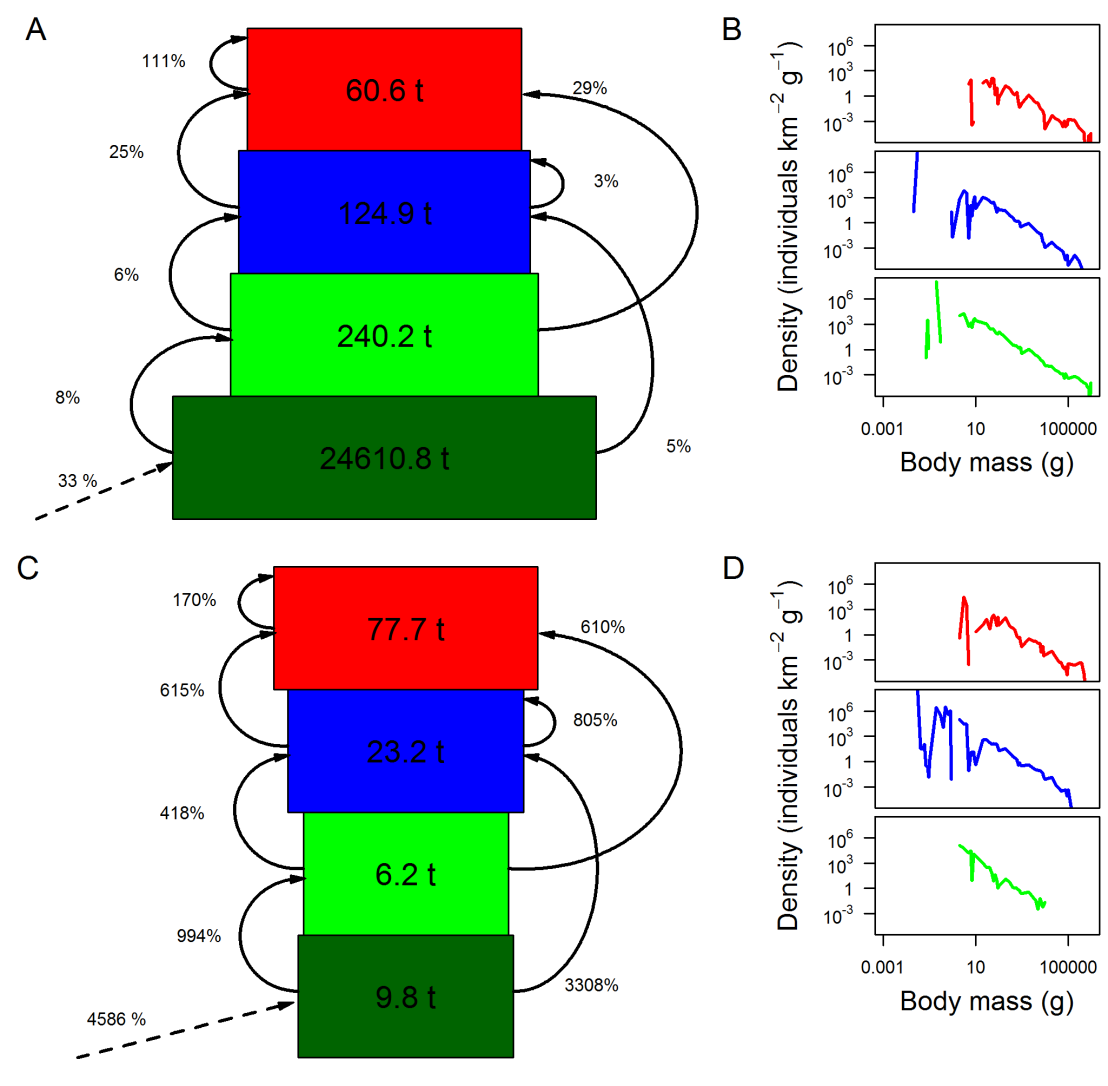
Community-level emergent properties. Community-level properties—(A, C) biomass pyramids and (B, D) body mass–density relationships across all cohorts belonging to each trophic level—emergent from the model for an example terrestrial (A, B) and marine (C, D) grid cell (grid cells T1 and M1 from Table 4). Results are from the final year of a 100-y model run. Dark green represents autotrophs, light green herbivores, blue omnivores, and red carnivores. In (A) and (C), standing stocks of biomass are indicated by the widths (after log-transformation) and numbers within the boxes; curved arrows and percent values represent the biomass transferred among or within trophic levels from herbivory and predation, as a proportion of the standing stock of the source of each flow; dashed arrows and percent values represent NPP of autotrophs as a proportion of the autotroph standing stock.

For the four focal grid cells, terrestrial ecosystems exhibited a pyramid of biomass across the different trophic levels (Figure 4A), which is widely accepted to be present in terrestrial systems [80]. The predicted herbivore biomass as a proportion of producer biomass (0.98%) was consistent with empirical terrestrial estimates (median value = 0.93%) (Table S5) [73]. However, predicted biomass of large-bodied herbivores was four to six times higher than estimated from a field study [72]. Consistent with current opinion and observations [78],[81]–[84], marine ecosystems exhibited an inverted pyramid of biomass structure [73],[82], with the highest biomasses in the highest trophic levels (Figure 4C). Marine systems exhibited relatively faster flow rates of productivity from autotrophs to higher trophic levels compared with terrestrial ecosystems—and at rates much higher than those estimated to date (Figure 4A and 4C) [85]. Predicted herbivore biomass as a proportion of producer biomass for the marine grid cells was much higher than in terrestrial ecosystems (63%), and of a similar magnitude to empirical estimates (median value = 52%) (Table S5) [73].

Expressed as abundance rather than biomass, and consistent with theoretical expectations [86],[87], trophic pyramids were not inverted in either realm: that is, communities contained a greater number of herbivores than carnivores (Figure S4).However, omnivores were more abundant than herbivores, by a factor of 3 in the terrestrial cell and by two orders of magnitude in the marine cell. This was because the average size of omnivores was smaller than the average size of herbivores or carnivores.

In both terrestrial and marine grid cells, densities of organisms showed a negative, approximately log-linear relationship with individual body mass (Figure 4B and 4D), the slopes of which fell within observed ranges in fish community assemblages from some sources (Figure S5) [75], although not from others (Table S6) [74].

### Geographical Patterns in Ecosystem Structure Across 14 Sites

**Figure 5:**
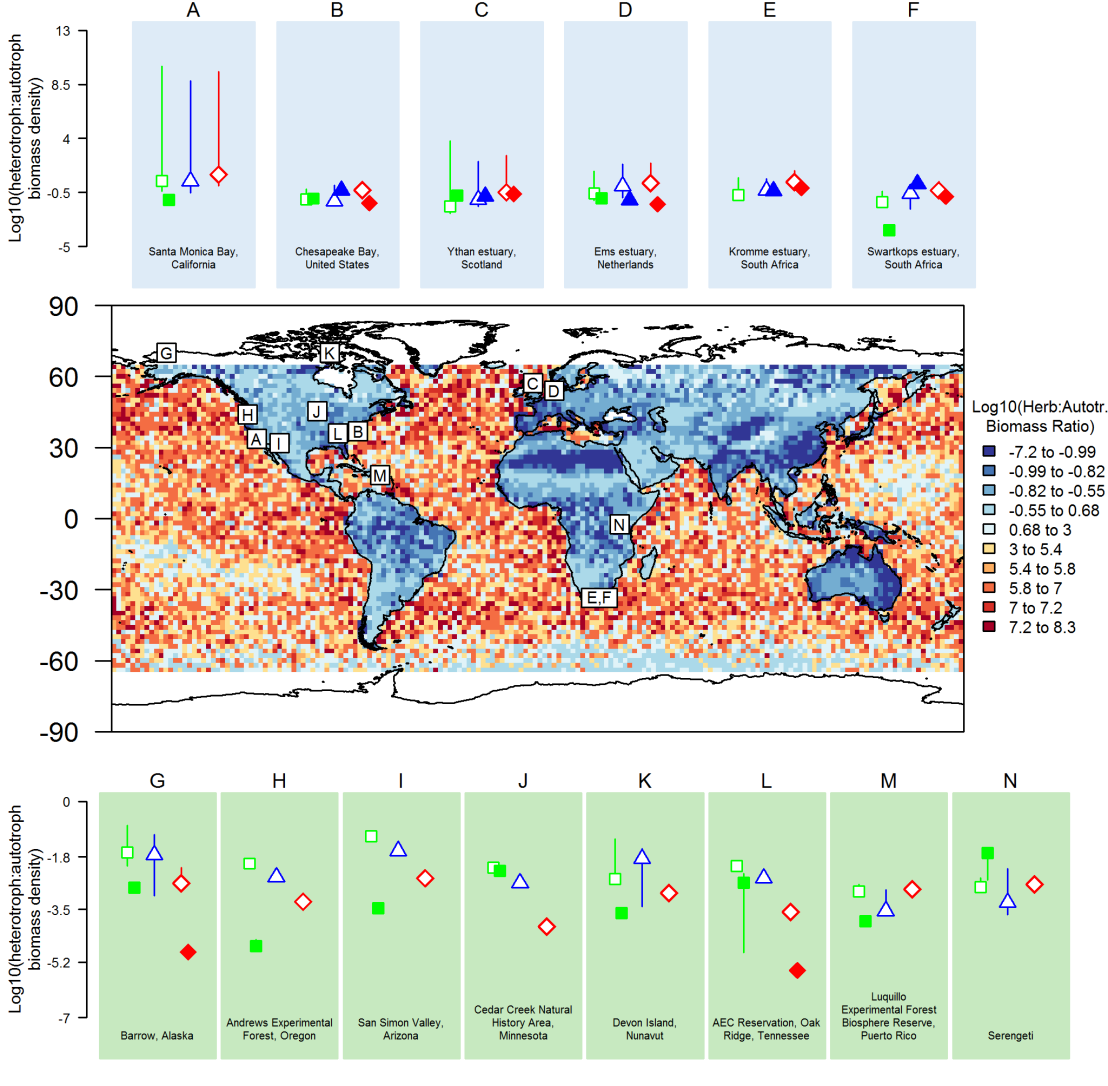
Global heterotroph∶autotroph biomass ratios. Comparisons of modelled (open) and empirical (filled) heterotroph to autotroph biomass ratios in marine (A–F) and terrestrial (G–N) environments (Table S3). Green squares are herbivore to autotroph ratios, blue triangles are omnivore to autotroph ratios, and red diamonds are carnivore to autotroph ratios. Modelled ratios are medians from 10 simulations, and vertical lines are 1 standard deviation over these simulations. Empirical ratios are individual estimates or, where more than one estimate was available, the median of these with sample sizes of H (*n* = 5), K (*n* = 2), L (*n* = 2), and N (*n* = 3), and vertical lines indicate maximum and minimum empirical estimates. Comparison locations are shown on a map of the predicted ratio of herbivore to autotroph biomass constructed from the global simulation (Study 4, Table 3).

Predicted ratios of heterotroph to autotroph biomass were broadly consistent with empirical estimates in many of the terrestrial and marine locations (Figure 5). For terrestrial ecosystems, the model and empirical data were in closest agreement in the two savannah ecosystems (Figure 5J and N). For the other ecosystem types—desert, tundra, deciduous forest, and tropical forest—there was lower agreement between model and empirical estimates of trophic structure, with modelled heterotroph to autotroph biomass ratios generally greater than empirical estimates, sometimes by orders of magnitude (Figure 5).

Modelled ecosystems in the marine realm generally showed closer agreement with empirical estimates than in the terrestrial realm. However, in both Santa Monica Bay, San Francisco (Figure 5A), and the Swartkops estuary in South Africa (Figure 5F), median modelled herbivore to autotroph biomass ratios were three orders of magnitude larger than empirical estimates.

### Trophic Structure Along Productivity Gradients

**Figure 6:**
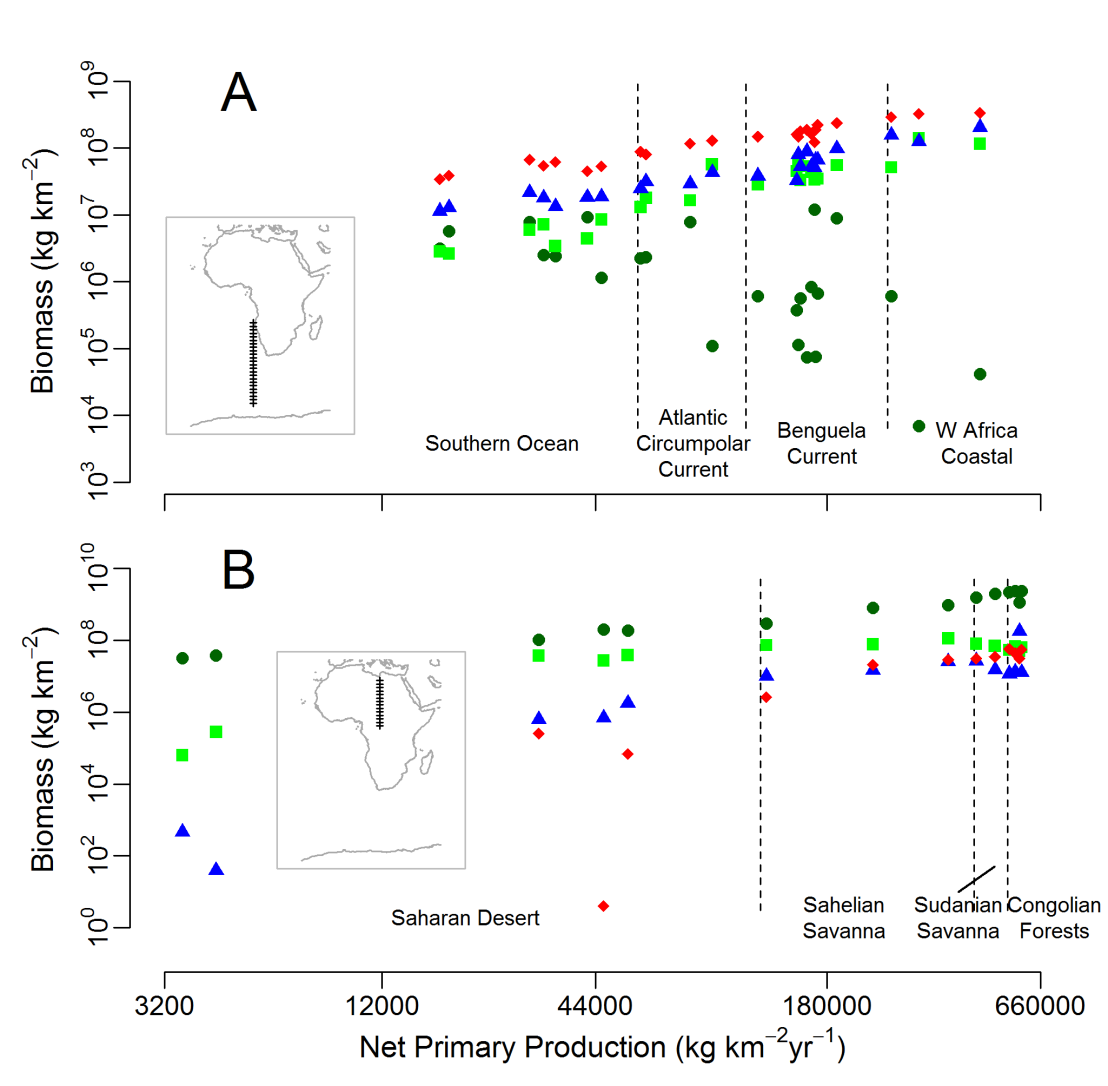
Ecosystem structure along productivity gradients. Variation in emergent ecosystem structure along productivity gradients in the marine environment (A) from the Southern Ocean to the West African Coast and in the terrestrial realm (B) from the Saharan Desert to the Congolian Forests. Transect locations are presented on the maps set into each panel. Dark green circles correspond to autorotroph biomass, light green squares correspond to herbivore biomass, blue triangles to omnivore biomass, and red diamonds to carnivore biomass. Broad biogeographic regions are roughly distinguished using dashed vertical lines.

The structure of both marine and terrestrial ecosystems showed marked changes along a gradient of increasing NPP (Figure 6). Marine ecosystems showed increasing biomass for all three heterotroph types (carnivores, omnivores, and herbivores), and flat then declining and highly variable autotroph standing biomass (Figure 6A). Terrestrial ecosystems also showed a trend of increasing heterotrophic biomass with productivity (Figure 6B). Carnivores increased in biomass with productivity more steeply than for other trophic levels, and were entirely absent from the lowest productivity desert ecosystem. In the most productive terrestrial ecosystems, carnivores typically had higher biomass densities than omnivores.

### Global Ecosystems

**Figure 7:**
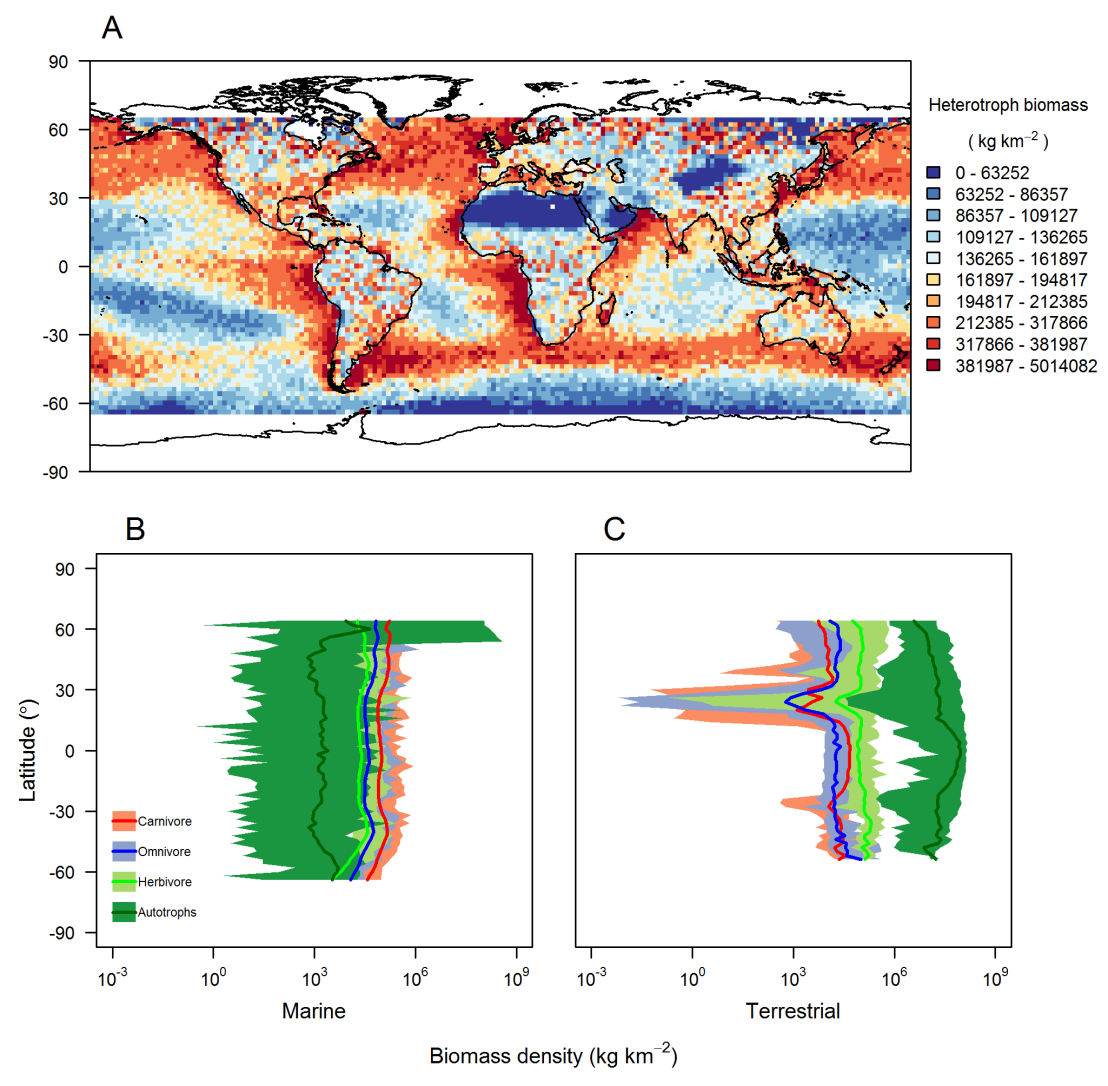
Emergent global-level ecosystem properties. Properties emergent from the model after a 100-y global (65°N to 65°S) simulation using a grid-cell resolution of two degrees. (A) The spatial distribution of annual mean heterotroph biomass density; breaks in the colour scheme were based on quantiles in the data. (B, C) Latitudinal gradients in biomass density; solid lines represent means for each trophic level, and shading represents the range of values across all longitudes in each latitude band.

Global patterns of total heterotroph biomass, averaged across the final year of the simulation (Figure 7A), were similar to patterns of primary productivity (Figure S6). In the marine realm, our modelled estimate of median heterotroph biomass density was 167,147 kg km^−2^, approximately 7–30 times greater than previous modelled estimates, which range from 5,500–25,000 kg km^−2^ [76],[88]–[90]. However, a recent empirical study into mesopelagic fish biomass suggests that some fish biomass densities are likely to be an order of magnitude higher than these previous estimates [90] and so our prediction is plausible. Global median ratios of herbivore to primary producer biomass estimated by the model were 0.8% for terrestrial and 189% for marine ecosystems, compared to 0.93% and 52% for empirical estimates (Table S5) [73]. Our modelled estimate of median total terrestrial heterotroph biomass density was 151,089 kg km^−2^, a prediction which, as far as we are aware, has never been made previously.

In the marine realm, high heterotroph biomass is predicted in upwelling systems and areas of high annual productivity (e.g., the North Atlantic). In the terrestrial realm, predicted heterotroph biomass was highest in naturally forested areas and lowest in deserts. There was no clear latitudinal gradient of biomass density in either system, but latitudinal variability was substantially greater in the terrestrial realm. At subtropical latitudes in the northern hemisphere in the terrestrial realm, there was a band in which carnivores had higher biomass density than omnivores, whereas elsewhere omnivores had greater biomass density. This switch in the relative dominance of omnivores in the northern hemisphere coincided with a decline in mean herbivore biomass density. No discernible decline in mean herbivore biomass densities was observed at subtropical latitudes in the terrestrial southern hemisphere.

Not all grid cells conformed to the pattern of inverted biomass pyramids in the marine realm and noninverted biomass pyramids in the terrestrial realm. Out of all terrestrial cells modelled, 9% were predicted with more omnivore than herbivore biomass and 46% with greater carnivore than omnivore biomass (Figure S7). Conversely in the marine realm, 12% of cells had less herbivore than autotroph biomass, 10% of cells had less omnivore than herbivore biomass, and 0.4% of cells had less carnivore than omnivore biomass (Figure S7). The spatial extent and frequency of cells in the marine realm with noninverted pyramids was significantly higher when dispersal was prevented from occurring (Figures S8 and S9). There was also evidence that noninverted trophic structure was more likely when the turnover rate of phytoplankton was lower and when the rate and efficiency with which matter is transferred through the system were reduced (Figure S10).

## Discussion

We have shown that it is possible to derive global predictions about the emergent properties of ecosystem structure and function from a GEM based on processes of, and interactions among, individual organisms, without any model-imposed constraints on those properties.

### Stability of Emergent Dynamics

The model reached a dynamic steady state in all grid cells, with the persistence of all trophic levels, which is expected in the absence of perturbation [91]. Real ecosystems are not expected to exhibit such stable dynamics because they are subject to numerous interannual environmental fluctuations and perturbations. These were not incorporated for this study, but could be in the future. The global simulations (which included dispersal) converged to equilibria with similar characteristics to the focal grid cells, although with higher biomass densities. The higher biomass must have been a result of dispersal, as this is the only difference between the focal-cell simulations and may be owing to a rescue effect from neighbouring grid cells. Nonetheless, the similarity of the simulations with and without dispersal provides support for the use of isolated focal grid cells in more detailed studies.

One form of instability in the dynamics was the large temporal variation in biomasses in the high-productivity, seasonal marine grid cell. Plankton and zooplankton are known to exhibit marked biomass fluctuations, but the modelled variation was much greater, and has a different temporal signature (chaotic variation within the productive season, whereas real cycles are repeatable year to year). This appears to be an artefact of the long time step used, allowing massive unconstrained changes in biomass over a single time step. Model simulations carried out using a daily time step (only feasible over shorter time periods and for single grid cells due to the computational demand) produced substantially more stable dynamics. However, the mean predicted biomass was similar between simulations using different time steps, suggesting that most of the properties of our results are robust. Nevertheless, future research is needed into numerical and computational methods for more appropriately simulating small organisms in GEMs (see Table 7 and Text S2).

### Emergent Life Histories and Trophic Structure

Many aspects of the life history of individuals emerge from the underlying ecological processes and interactions with other organisms. For example, growth rates are determined by a combination of food assimilation rates and metabolic losses, which themselves depend on the abundance and properties of the other individuals at the location, and are also constrained by the maximum possible rate of assimilation and environmentally determined metabolic rate. Modelled growth rates varied widely among cohorts, between the maximum and minimum theoretically possible rates (Figure S3), but showed good correspondence with observed values (Figure 3A). Predicted mortality rates were much higher than empirically observed rates, especially for larger organisms (Figure 3C). This may reflect a mismatch with the data, as discussed below; in this case, empirical mortality rates were observed in laboratory conditions in the absence of predation mortality. Comparing the empirical data and model predictions at a higher level of resolution, for example within functional groups or biomes, might help us to better diagnose discrepancies in the future.

Growth rates were slightly faster and times to maturity slightly shorter than those from observation. Modelled rates of biomass transfer calculated for the two focal grid cells were higher than empirically observed rates, particularly in the marine realm (Figure 4) [85]. At least in part, this discrepancy may result from a bias in the data toward larger organisms, leading to empirical estimates underestimating real biomass flow rates.

Similarly, the negative log-linear relationship between individual body mass and organism density is consistent with ecological theory [92], although uni- [93],[94] or multimodal [95],[96] relationships have been observed. The modelled slopes were shallower than empirical estimates for a log-linear relationship, which again might indicate that turnover rates in the model were overestimated, causing greater suppression of smaller organism abundance and higher abundance of larger organisms than in real ecosystems.

Another difference between the model and data was that the modelled biomass of large-bodied herbivores was several times higher than observations [72]. Modelled ratios of herbivore to autotroph biomass were also higher than empirical estimates in most cases, especially for terrestrial ecosystems. There are three potential explanations for this. The first is that herbivores, like predators, are eating too efficiently in the model, which might result from incorrect functional forms or missing processes. The second explanation is that because the available data are extremely sparse, the data are thus potentially not representative of the system. Thirdly, human impacts might have reduced large herbivore biomass in the empirical data compared to the undisturbed ecosystem assumed in these simulations of the model.

The closer match between modelled and empirical trophic structure for marine and savannah ecosystems, compared to forests, might be because these ecosystems conform much better to the assumption that individuals are well mixed. This assumption implies that herbivores can find all plant material and predators can find all prey. In all ecosystems, but most obviously in forests, many leaves are physically out of reach of many herbivores, and many prey are able to find refuge from predation. This may act to slow down biomass turnover in forests in comparison to the ocean and savannas, in a way that cannot be captured by a model that assumes complete mixing within a grid cell. On the other hand, the discrepancy could also be caused by incomplete empirical data. It is easier to estimate whole-ecosystem properties in savannas (where animals tend to be highly visible) and in the ocean (which can be trawled with nets) compared with forests. Alternatively, the terrestrial plant model might be inaccurately capturing the allocation of productivity to structural or nonstructural matter at these specific forest sites. However, this appears less likely, as the allocation function of this model has been rigorously constrained using a global dataset, and evaluation shows the model predicts well for this property [36]. We note, however, that at finer, site-level scales, plant allocation strategies will certainly be more heterogeneous and will certainly be different from the larger scale average predicted by the global model.

It is important to note that differences between the marine and terrestrial realms were not imposed on the model as top-down limits on the structure of ecosystems but rather emerged from individual-scale ecology. A necessarily different set of representative functional groups was defined for the marine environment compared to the terrestrial environment, but all functional groups in the model followed the same fundamental ecological functions. The only other encoded differences between marine and terrestrial cohorts were the proportion of total autotroph biomass available for consumption by a given herbivore cohort, the different optimal prey body sizes for predators, and the assumption that marine ectotherms are not thermally restricted in their capacity to function. The primary cause of the much lower ratio of herbivore biomass to autotroph biomass in the focal terrestrial cells versus in the oceans was the allocation by terrestrial plants of primary productivity to structural tissues, which are inedible to all herbivores in the model (Figure S11). This assumption probably made it more likely that terrestrial cells would exhibit a conventional pyramid of biomass structure. However, the model predicted considerable variation in the shape of the trophic biomass pyramid across terrestrial and marine cells, including an inverted structure in some terrestrial cells and a noninverted structure in some marine cells (Figure S7).

### Emergent Global Patterns: Model Results and Predictions

The modelled global estimates of biomass density represent the first attempt to assess the scaling of ecosystem properties from individuals to communities at a global level, and to apply a general ecological modelling methodology consistently across both the marine and terrestrial realms.

Some of these properties have been estimated previously in specific locations, but none have been estimated globally using mechanistic models (although marine animal biomass and biomass densities have been predicted mechanistically [76],[88],[89]). In addition, we have calculated emergent properties that are, as far as we are aware, unprecedented (Table 8), and we discuss these below.

**Table 8:** Novel predictions and datasets needed to evaluate them.

Prediction	Data to Evaluate
Median terrestrial total heterotrophic biomass density is predicted to be 151,089 kg km^−2^ and maximum terrestrial heterotrophic biomass densities are found in ecosystems with intermediate levels of NPP (Figure 7A and Figure S6A)	Empirical or modelled information on the total biomass of heterotrophic organisms in a sample of terrestrial biomes, from which median terrestrial biomass density could be inferred
Median marine total heterotophic biomass density is predicted to be 167,147 kg km^−2^, approximately 7–30 times greater than previous modelled estimates	Empirical information on the total biomass of heterotrophic organisms in a sample of marine locations, from which median marine biomass density could be inferred
Omnivorous organisms account for greater biomass than carnivores in low to mid productivity terrestrial environments, for example in the Saharan desert—Sahelian savanna transition (Figure 6B), or more widely in the mid to high latitudes in the northern hemisphere (Figure 7C). At equatorial latitudes and extending to 30°S, carnivorous organisms constitute greater biomass than omnivores on average	Ecosystem trophic structure or food web structure for low to mid productivity terrestrial environments in equatorial latitudes and mid latitudes in both hemispheres
Terrestrial biomass densities vary more than marine biomass densities across latitudes (Figure 7B and C)	Observations of total biomass contained in trophic groups at a set of sites within the same latitude band, carried out across a range of latitude bands. For the terrestrial realm, the band between 20° and 30°N has potentially substantial latitudinal variation in total biomass.

#### Heterotroph Biomass and NPP

At a global scale, the spatial pattern of heterotroph biomass was broadly consistent with observed NPP [97], which is unsurprising, as NPP is the basal resource in all of the modelled ecosystems. However, the relationship between primary productivity and heterotroph biomass was not simple: a given level of primary productivity could result in a wide range of heterotroph biomass (Figure S6A–D). This variation arises from differences in climate, which lead to differences in biomass allocation to different plant tissues and to variation in animal metabolic rates and levels of activity. The predicted peak in terrestrial heterotroph biomass densities observed in locations with intermediate levels of NPP is also likely to result from these factors; for example, plants in the most productive areas allocate a greater proportion of biomass to structural tissues, whereas plants in intermediate productivity ecosystems such as grasslands have a greater relative allocation to nonstructural tissues. Autotroph biomass was also influenced by herbivory, as demonstrated by removing all heterotrophs from the model and representing mortality of plants through herbivory using a constant loss term (Figure S6E and F). This experiment suggests the impact of herbivory on plants varies significantly across the world, indicating the potential importance of explicitly considering herbivores in carbon cycle models, such as DGVMs, or models of ocean biogeochemistry, which is not done at present. Exploring the impacts of heterotrophs on carbon cycling and predicted carbon dynamics is an important avenue of research for future development of GEMs.

#### Emergent Structure and Productivity

Modelled variation in trophic structure along gradients of productivity supported theoretical expectations that the NPP of systems will determine the length of trophic chains [9],[10],[98]. In low-productivity terrestrial systems, there is insufficient autotrophic biomass propagating to higher trophic levels to support carnivores, whereas in high-productivity ecosystems, carnivore biomass is greater than omnivore biomass. In the marine realm, autotroph biomass decreased with increasing primary productivity beyond NPP of 40,000 kg km^−2^ y^−1^, inconsistent with expectations from the trophic release hypothesis (Figure 6B) [11]. The model predicts for marine ecosystems that with increasing primary productivity herbivore biomass increases and carnivore biomass, as a proportion of total biomass, decreases. For terrestrial ecosystems, herbivore biomass increased to around 40,000 kg km^−2^ and then decreased, whereas the carnivore biomass proportion decreased only after around 550,000 kg km^−2^. The relationship predicted for marine ecosystems is in agreement with the general findings from available empirical data on changes in trophic structure associated with changes in productivity, derived from freshwater, intertidal, and reef fish communities [99]–[103]. However, our results indicate that a qualitatively different relationship between trophic structure and productivity appears to exist in the terrestrial realm. There are additional factors that influence modelled community structure along productivity gradients that will require detailed investigation in the future, including (i) seasonality, which will vary among different modelled locations with similar productivity; (ii) climatic differences, which might affect the composition of autotroph biomass in terrestrial ecosystems; and (iii) dispersal regimes.

#### Emergent Latitudinal Patterns of Community Structure

Predictions of latitudinal variation in community structure (as shown in Figure 7B and 7C) give an insight into the mechanistic basis of these patterns at a global scale. On land, there was greater variation in the biomass density of all trophic groups, both latitudinally and longitudinally, compared to in the oceans (Table 8). The greater variation in terrestrial biomass densities was likely driven by greater environmental heterogeneity in the terrestrial realm and the greater range and speed of dispersal processes (e.g., advective dispersal) in the oceans. For example, both temporal [104],[105] and spatial variability [106] in environmental temperature is higher on land versus the ocean.

In the mid to high latitudes of the terrestrial northern hemisphere, omnivorous organisms account for greater biomass than carnivores. However, in equatorial latitudes and extending to 30°S, carnivorous organisms constituted a greater fraction of the total ecosystem biomass than omnivores (Figure 7C and Table 8). The equatorial and low southern hemisphere latitudes correspond to more stable environmental conditions [106] compared with mid to high northern latitudes. Therefore, the model appears, in the terrestrial realm, to be selecting for generalist feeding strategies in more variable environments, while favouring specialism where the environment is more stable.

#### Mechanisms Giving Rise to Inverted Marine Trophic Structure

The model showed that dispersal of organisms plays an important role in the spatial extent and frequency of inversion of marine trophic structure (Figures S8 and S9), which is consistent with the hypothesis that the import of allocthonous organic matter supports relatively higher heterotrophic biomass in oligotrophic freshwater systems, which has been postulated previously but not investigated in detail [77]. Furthermore, we show that noninverted trophic biomass structure is more frequent when the turnover rate of phytoplankton is reduced and when the rate and efficiency of biomass transfer through the ecosystem is lower (Figure S10), in agreement with current theory [77]–[79]. The generality of inverted trophic pyramids in the marine realm remains unknown, but there are numerous examples of herbivore biomass being larger than producer biomass in ocean environments [73]. Inversion at the top of the trophic pyramid has apparently been observed in some near-pristine reef habitats [84], though this may simply be an overestimation artefact of the visual census approach used [107].

### Future Model Development

In developing the first global GEM, our strategy was to start with a “simple” model that can be improved later. To keep it simple, we excluded numerous aspects of ecology—some of which are captured by existing models of particular ecosystems [14],[15]. Adding this ecology back into our model would, arguably, make it more realistic. Then again, attempting to include all known ecological processes occurring in every ecosystem around the world would not only be completely impractical but would also be excessive for most of the purposes for which a GEM is intended. Thus, we would expect the set of ecological processes represented in GEMs to evolve over time and to depend on the ways in which GEMs come to be used. Moreover, there are several other important ways in which our model could be improved, as outlined in Table 7, and discussed below.

#### Future Model Development: Ecology

The physical environment is currently represented very simply in the model. For example, in reality, variation in habitat structure within grid cells will alter interactions between organisms. And a fully integrated and explicitly resolved mechanistic model of phytoplankton is required in future iterations of the model, as is representation of nonphytoplanktonic coastal autotrophs (e.g., seagrass beds or coral reefs) and detritivores. The detrital loop in particular is important in “closing the system.” Other ecological processes that should be incorporated in future iterations of the model include ecological stoichiometry; intelligent behaviour, such as directed dispersal, hibernation, and stasis; and complex predator–prey and herbivore–plant interactions (Table 7).

#### Future Model Development: Data

Robust data-constrained parameterisation of the model and rigorous evaluation of our novel but testable outputs was not possible because the necessary data were not generally available. Therefore, another set of developments are needed around the acquisition and collation of data for use in GEM modelling (Table 8 and Table 7), which are reflected by the biodiversity informatics community [108].

#### Future Model Development: Numerical and Mathematical Methods

The development of the Madingley Model was enabled by combination of numerical methods that is, far as we are aware, novel (e.g., the cohort approach to trait-driven interactions, combined with cohort merging, randomly ordered updating, the treatment of autotrophs as continuous state variables, and exponential differencing of loss rates within time steps; see Text S2 for further examples). Given the novelty of this modelling paradigm in ecology, at least five areas of development will be required: detailed analysis and understanding of the mathematical representations of ecology, analytical tractability of predicted ecosystem properties, the implications of alternative abstractions and numerical methods used to implement them, the infrastructure to allow flexibility in scale, and the infrastructure to constrain and evaluate the model so that the benefits of additional components or realism can be assessed objectively (Table 7). In addition, it would be interesting to explore the approach referred to as “parameterization” in the biogeosciences modelling community—that is, to fit phenomenological relationships to the output of GEMs, thus allowing approximate versions of them to be run much more quickly, or within the context of more general ESMs.

#### Future Model Development: Community

The further development of GEMs discussed above will be expedited by a community of researchers improving upon the model presented here or developing alternative GEMs. We call for such a community of ecologists, biologists, mathematicians, and computer scientists to form around the GEM concept so that this class of model can be rapidly advanced to better meet the pressing needs of conservationists and policymakers. In this spirit, we have developed our model architecture in such a way that it is relatively easy to alter the representation of ecological processes and have made our model code freely available to the community (www.madingleymodel.org).

### Use of GEMs

Our model is the first step towards the development of a more ecologically refined GEM (and GEMs) that occupies a very different niche to the more specialised modelling approaches that have been used to inform biodiversity policy to date, and is more akin to the global climate models that inform global climate science and policy [8]. Our GEM necessarily made numerous simplifying assumptions in order to capture a broad range of ecological processes and organisms. This nevertheless provides a new approach to answering important outstanding questions in ecology and with which to begin to ask entirely new questions—questions that require an integrated, mechanistic understanding of whole ecosystems; the connections among them; and their response to environmental variation and natural or human perturbations. In the following sections, we briefly discuss how GEMs could be used to inform ecological science and conservation policy.

#### Use of GEMs: Ecology

GEMs will help in the development of ecological theory. The study of ecology is so broad that most pieces of ecological research necessarily focus on a small subset, whether in terms of scale, taxa, or the processes concerned. The implications of the findings in these disparate research areas for the longer term and larger scale dynamics of whole communities and ecosystems are hard to assess. This has led some ecologists to call for a renewed interest in “systems ecology,” which aims to study important ecological processes within the context of other important ecosystem and earth-system processes to enable a better understanding of the natural environment [109]. Models like ours provide one way to do this: new ecological findings can be used to modify the model, or different competing formulations of ecological theories (e.g., prey density or ratio-dependent predation functions [110]) can be represented in the model and the results assessed against known ecosystem structure. By doing so, the processes of critical importance for ecosystem structure and functioning could be identified and priorities for development of new ecological hypotheses or data-gathering determined. Our preliminary investigations of community trophic structure along productivity gradients and of the mechanisms giving rise to the inversion of marine cells illustrate the potential of GEMs in this regard.

#### Use of GEMs: Conservation

The simulations presented here are not directly relevant to conservation decision-making, as the model currently does not directly include anthropogenic influences. In principle, a model of this kind could be used to better inform the management of the world's ecosystems, in much the same way that mechanistic ESMs are used to predict and explore scenarios of anthropogenic climate change [7]. Forcing the model with time-varying historic or future projections of environmental variables would provide the kinds of novel and highly relevant outputs that could enable a mechanistic approach to conservation decision-making.

By taking a system-level approach, GEMs are uniquely able to simulate the interacting relationships between various simultaneous human pressures (such as climate change, land use change, and harvesting of wild animals) and various metrics of ecosystem structure and function, exploring the trajectories derived from external scenarios of human pressure. For example, they could predict (1) static measures of ecosystem properties, such as the variation in the functional traits represented in ecosystems, an important measure of biodiversity thought to be related to ecosystem functioning [26]; (2) metrics that are currently used to monitor the state of ecological systems, such as the total abundance of large endotherms as an analogue of the Living Planet Index [111]; (3) metrics relating to focal species, by examining the fate of organisms with similar traits to those species, although important species-specific factors may not be captured that way; and (4) dynamic ecosystem measures, such as stability (the magnitude of temporal variation in ecosystem properties) or resilience (predicted time to recover from a perturbation). A unique advantage of GEM-like models relative to statistical modelling approaches is their capacity to model completely novel perturbation scenarios—that is, perturbations for which there are no data, experiments, or observations.

The structure of this type of model also allows for a wide set of anthropogenic perturbations to be considered simultaneously. For example, such models will be able to simulate how whole ecosystems respond to, and feed back upon, the effects of changes in atmospheric carbon dioxide concentrations on plants; altered climate on all organisms and ecological processes; harvesting of animals, land-use change, and harvesting of vegetation; invasive species; and toxic pollutants. The effects of these disparate perturbations could be measured using a common set of metrics. Considering these impacts together in a GEM should allow for much more holistic understanding, and management, of the world's ecosystems, which will make GEMs a powerful tool for bodies such as the Intergovernmental Platform on Biodiversity and Ecosystem Services.

### Conclusions

We have developed the first General Ecosystem Model (GEM) that synthesizes fundamental aspects of ecological theory, to model, simulate, and predict how the structure and function of ecosystems at multiple scales emerges from the biology and interactions of individual organisms. Our model matches a broad range of empirical data well, to a first approximation. Where it departs from either observations or expectations, it does so in ways that afford interesting avenues for future ecological research, future model development, and empirical data collection. It also provides a set of novel predictions that can be independently assessed. We anticipate that this will be the beginning of a long-term exploration of GEMs, and we call upon ecologists, biologists, mathematicians, and computer scientists to join in creating a modelling community surrounding GEMs that can catalyse the development of more realistic, sophisticated, yet better understood models of ecosystems worldwide. Our hope is that GEMs will form the basis of new science in ecology and, in particular, science that proves actionable to those charged with conserving the biosphere on which we all depend.

## Supporting Information

Figure S1 **Cohort dispersal effects on autotrophic and heterotrophic biomass.** The difference between fracturing cohort and whole cohort dispersal expressed as a percentage of the whole cohort dispersal value. Percentage differences were calculated over a 10×10 grid of 1°×1° marine grid cells extending from 30° to 40°N and 40° to 30°W, using median and annual mean biomasses from an ensemble of 10 simulations for both fracturing cohort and whole cohort dispersal. Negative values therefore indicate lower biomass in the fracturing cohort ensemble median, whereas positive values indicate the opposite.
(TIFF)

Figure S2 **Cohort number effects on long-term means of trophic-level biomass.** Medians from the mean over the last 5 y of ensembles of 20 replicate simulations (points) and absolute ranges (error bars) of biomass densities for autotrophs (dark green lines and dark green squares), herbivores (green lines and green circles), omnivores (blue lines and blue triangles), and carnivores (red lines and red diamonds). Ensembles of replicates were run with a threshold of 500, 1,000, 5,000, or 10,000 cohorts per grid cell for terrestrial cell T1 and marine cell M1 (Table 4).
(TIFF)

Figure S3 **Emergent model growth rates compared to theoretical maximum rates.** (A) Absolute emergent model growth rate (grey crosses and diamonds) relationship with body mass compared with empirical (black points) and theoretical maximum (red line). (B) The relationship between emergent model growth rate as a fraction of theoretical maximum growth rate (grey open circles) and body mass for each trophic level in terrestrial or marine cells. Modelled emergent individual-level properties are sampled from 100-y model runs for the four focal grid cells (Table 4).
(TIFF)

Figure S4 **Trophic abundance pyramids.** Community-level abundance pyramids across all cohorts belonging to each trophic level emergent from the model for an example of terrestrial and marine grid cell (grid cells T1 and M1 from Table 4). Results are from the final year of a 100-y model run. Light green represents herbivores, blue represents omnivores, and red represents carnivores. Total abundance densities (1,000 s individuals/km^2^) are indicated by the widths (after log-transformation) and numbers within the boxes.
(TIF)

Figure S5 **Comparison of model predicted with empirical normalised body mass spectra (NBS).** Frequency distribution of the slope of NBS from [75] with model-derived NBS slope values, calculated following Sprules and Munawar [114], for carnivores (red), omnivores (blue), and herbivores (green). Triangles correspond to slopes for the low productivity, aseasonal marine cell (M1, Table 4) and circles to the high productivity, aseasonal terrestrial cell (T1, Table 4).
(TIF)

Figure S6 **Relationships between predicted biomass densities and NPP.** The global relationship between total heterotrophic biomass and NPP split between terrestrial and marine realms (A). The global relationship between the ratio of herbivore to autotroph biomasses and NPP split between terrestrial and marine realms (B). The relationships between different trophic levels and NPP across terrestrial (C) and marine (D) environments. The relationship between autotroph biomass and NPP across terrestrial (E) and marine environments (F) with heterotrophs modelled explicitly “full” and constant proportional autotroph herbivory loss rates of 0.25, 0.5, and 0.75.
(TIFF)

Figure S7 **Frequency distributions of trophic biomass structure.** Frequency distributions of log-transformed ratios of trophic-level biomasses in terrestrial grid cells (brown) and marine grid cells (blue), for H∶A = herbivore to autotroph, O∶H = omnivore to herbivore, C∶H = carnivore to herbivore, and C∶O = carnivore to omnivore biomass ratio. Red dashed lines indicate where the biomass ratio equals 1.0, which means equality of the two trophic-level biomasses.
(TIFF)

Figure S8 **Spatial extent of un-inverted marine trophic structure for the bottom two trophic levels: herbivores and autotrophs.** Spatial locations (green points) of un-inverted herbivores to autotroph trophic structure (i.e., where there is less herbivore than autotroph biomass) in (A) a simulation where dispersal was permitted (Study 4, Table 3) and (B) when dispersal is not modelled.
(TIFF)

Figure S9 **Frequency distribution of marine trophic structure in the absence of dispersal.** Frequency distributions of log-transformed ratios of trophic-level biomasses in marine grid cells with dispersal (upper set of histograms—Study 4, Table 3) and marine grid cells without any dispersal modelled (lower set of histograms). H∶A, herbivore to autotroph; O∶H, omnivore to herbivore; C∶H, carnivore to herbivore; C∶O, carnivore to omnivore biomass ratio. Red dashed lines indicate where the biomass ratio equals 1.0, which means equality of the two trophic-level biomasses.
(TIFF)

Figure S10 **The effects of turnover rates and trophic transfer efficiencies on marine trophic structure.** Box and whisker plots of the predicted ratios of trophic levels (H∶A, herbivore to autotroph; O∶H, omnivore to herbivore; C∶H, carnivore to herbivore; C∶O, carnivore to omnivore biomass ratio) for ensembles of 10 replicate simulations with different model assumptions investigating the mechanisms giving rise to inverted marine trophic biomass structure: N, the full model for a single grid cell; H, herbivore assimilation efficiency reduced to 20% (from 60–70% omnivore–herbivore); HP, herbivore and predator assimilation efficiency reduced to 20% (from 60–80% omnivore–carnivore); A, attack rates of herbivores and predators decreased by two orders of magnitude; AHP, combined reduction of attack rates, herbivore assimilation, and predator assimilation as above. Dark bars indicate median values, boxes the interquartile ranges, and whiskers the maximal range. Upper panels correspond to grid cell M1 and lower panels to grid cell M2 (Table 4).
(TIFF)

Figure S11
**Community-level properties for cell T1 with all edible plant matter available for herbivory.** Trophic pyramid and size distribution spectra for focal cell T1 with the value of parameter 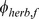 equal to 1 for terrestrial herbivores, which means that each terrestrial herbivore cohort experiences 100% of the edible plant matter in the grid cell when it is eating. The release of this parameter does not affect the low herbivore to primary producer ratio for terrestrial communities. Here, the ratio is 1.0%, marginally higher than that calculated for the same location using a value for 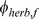 of 10% (Figure 4).(TIFF)

Table S1 **Environmental data sources.** External global environmental data sources used within the model. Units represent those used within the Madingley model, not those of the original source data. ^a^ Environmental variables were long-term/multidecadal average values.
(DOCX)

Table S2 **Model parameters and their values.**
(DOCX)

Table S3 **Empirical estimates of trophic-level biomasses in globally widespread ecosystems in both marine and terrestrial environments.** Notes: 1. Consider only the pelagic producers. Assume that dissolved organic carbon (DOC) and suspended particulate organic carbon (POC) are available to the pelagic community. Do not include benthic suspension feeders. Assume micro-zooplankton are herbivores (as defined in our model). For omnivores and carnivores, assume that the proportion of biomass that is supported originally by pelagic primary production is equal to the proportional rate of consumption of pelagically derived foods relative to consumption of foods from all sources. 2. From Table 12.2 (plants taken as above ground vascular + Moss + Algae + Lichens) of Chapin et al [115]. 3. Herbivore biomass taken from [73]. 4. Producer and herbivore biomasses from [73]. 5. Producer biomass of 9,999 g C m-2 comes from Figure 8 of Frangi and Lugo [116]. Montane palm floodplain forest; herbivore biomass taken from [73].
(DOCX)

Table S4 **Comparison of emergent individual-level properties from the model with observations.** The slopes and intercepts of the relationships between predicted properties and body mass compared to empirical data. The probability that the slope and intercept of the predicted relationship were different from those for the empirical data was calculated as the *t* statistic of linear models fitted to combined model and empirical data for each emergent property using a categorical factor to indicate a model or empirical datum. The probability that the *t* statistic for the model including the categorical factor indicates the significance of the difference.
(DOCX)

Table S5 **Summary statistics of empirical community herbivore and primary producer biomasses.** Summary statistics, derived from [73], for those ecosystem types most closely representing the ecosystems within the two grid cells shown in Figure 3. PB, primary producer biomass; HB, herbivore biomass. Median herbivore biomass in temperate and tropical grassland ecosystems is 52.3% of that in communities of marine phytoplankton, whereas median primary producer biomass is 57 times larger in the terrestrial compared to the marine ecosystem.
(DOCX)

Table S6 **Comparison of abundance–density slopes predicted by the model with observed slopes.** Abundance–density relationships predicted by the model (for cells T1 and M1, Table 4) compared with observations. Empirical abundance–density relationships were derived from Jennings et al. [74]. The reported total biomass versus body mass relationships were converted to abundance versus body mass relationships, by dividing the total biomass in each mass bin by the central mass of that mass bin, which can be approximated as subtracting 1 from the slope of total biomass against body mass.
(DOCX)

Text S1 **Technical and mathematical details of the model.**
(DOCX)

Text S2 **Model time step effects.**
(DOCX)

## Abbreviations

DGVMs: dynamic global vegetation models
DOC: dissolved organic carbon
ESMs: Earth System Models
EwE: Ecopath With Ecosim
GEM: General Ecosystem Model
NBS: normalised body mass spectra
NPP: net primary productivity
POC: particulate organic carbon
