# Supplementary figures and images for: Emergent Global Patterns of Ecosystem Structure and Function from a Mechanistic General Ecosystem Model
Source: PLoS Biol. 2014 Apr 22;12(4):e1001841. doi: 10.1371/journal.pbio.1001841 (PMC3995663; doi:10.1371/journal.pbio.1001841)

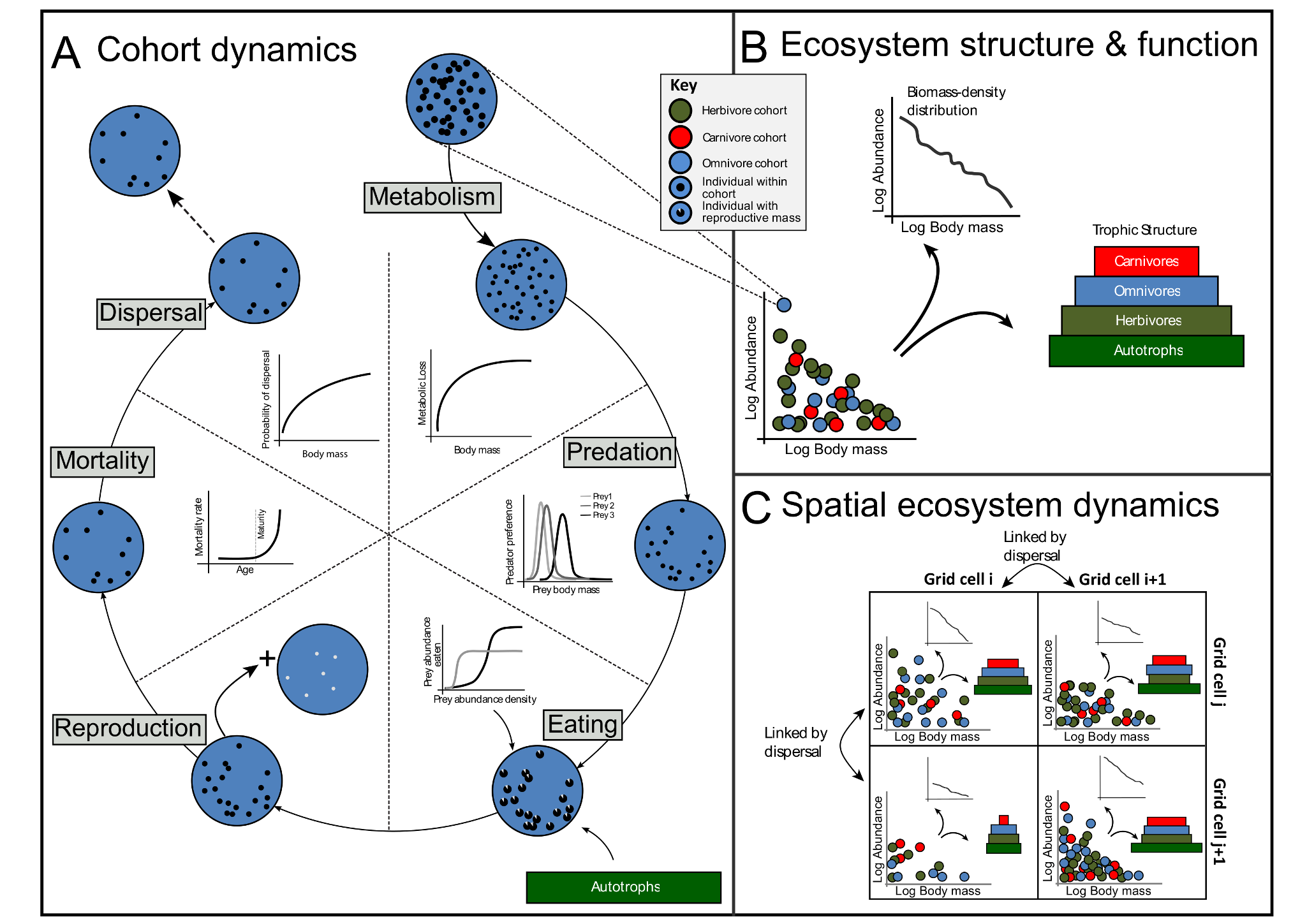

Supplement: Figure S1 — Cohort dispersal effects on autotrophic and heterotrophic biomass. The difference between fracturing cohort and whole cohort dispersal expressed as a percentage of the whole cohort dispersal value. Percentage differences were calculated over a 10×10 grid of 1°×1° marine grid cells extending from 30° to 40°N and 40° to 30°W, using median and annual mean biomasses from an ensemble of 10 simulations for both fracturing cohort and whole cohort dispersal. Negative values therefore indicate lower biomass in the fracturing cohort ensemble median, whereas positive values indicate the opposite. (TIFF) [file pbio.1001841.s001.tiff]

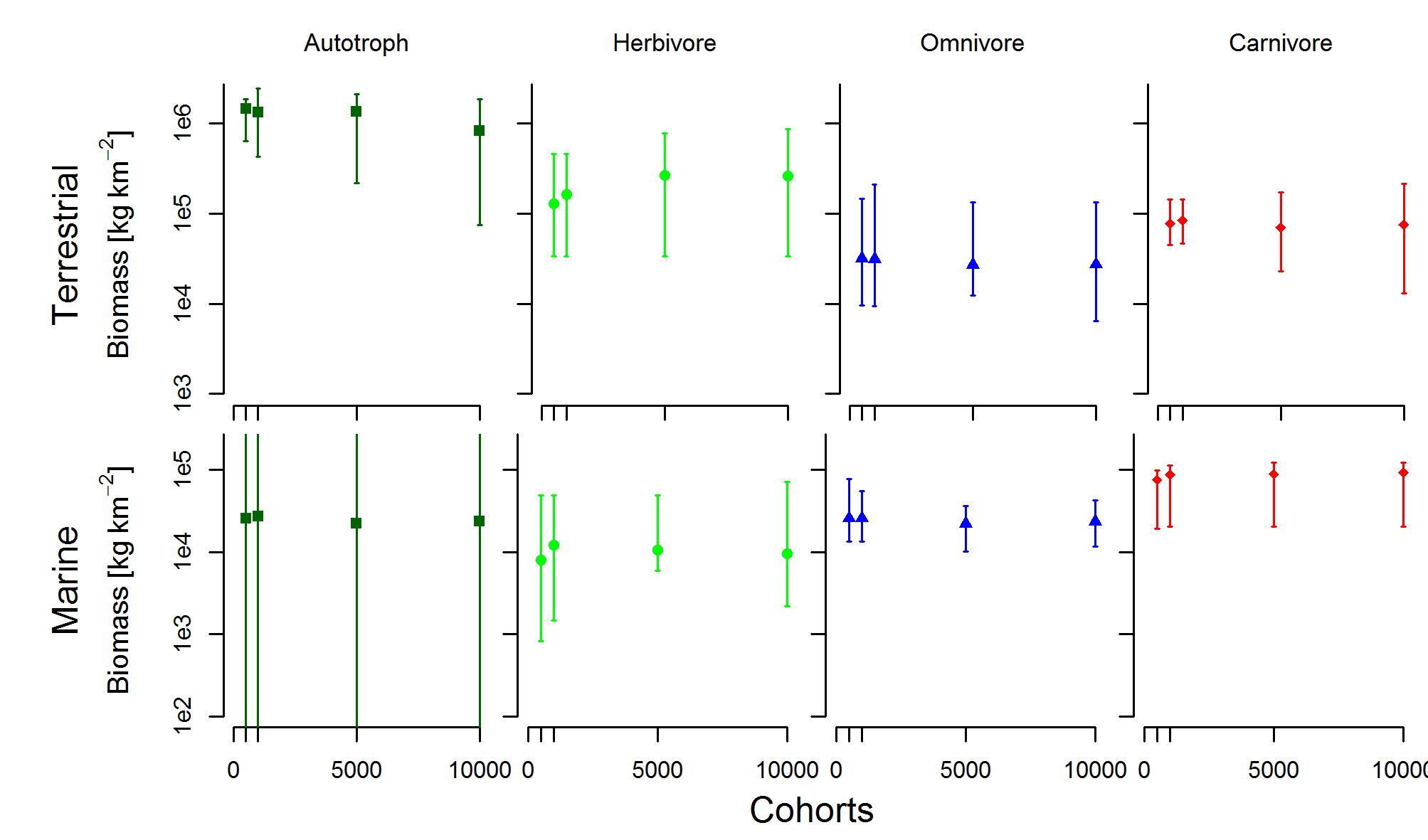

Supplement: Figure S2 — Cohort number effects on long-term means of trophic-level biomass. Medians from the mean over the last 5 y of ensembles of 20 replicate simulations (points) and absolute ranges (error bars) of biomass densities for autotrophs (dark green lines and dark green squares), herbivores (green lines and green circles), omnivores (blue lines and blue triangles), and carnivores (red lines and red diamonds). Ensembles of replicates were run with a threshold of 500, 1,000, 5,000, or 10,000 cohorts per grid cell for terrestrial cell T1 and marine cell M1 (Table 4). (TIFF) [file pbio.1001841.s002.tiff]

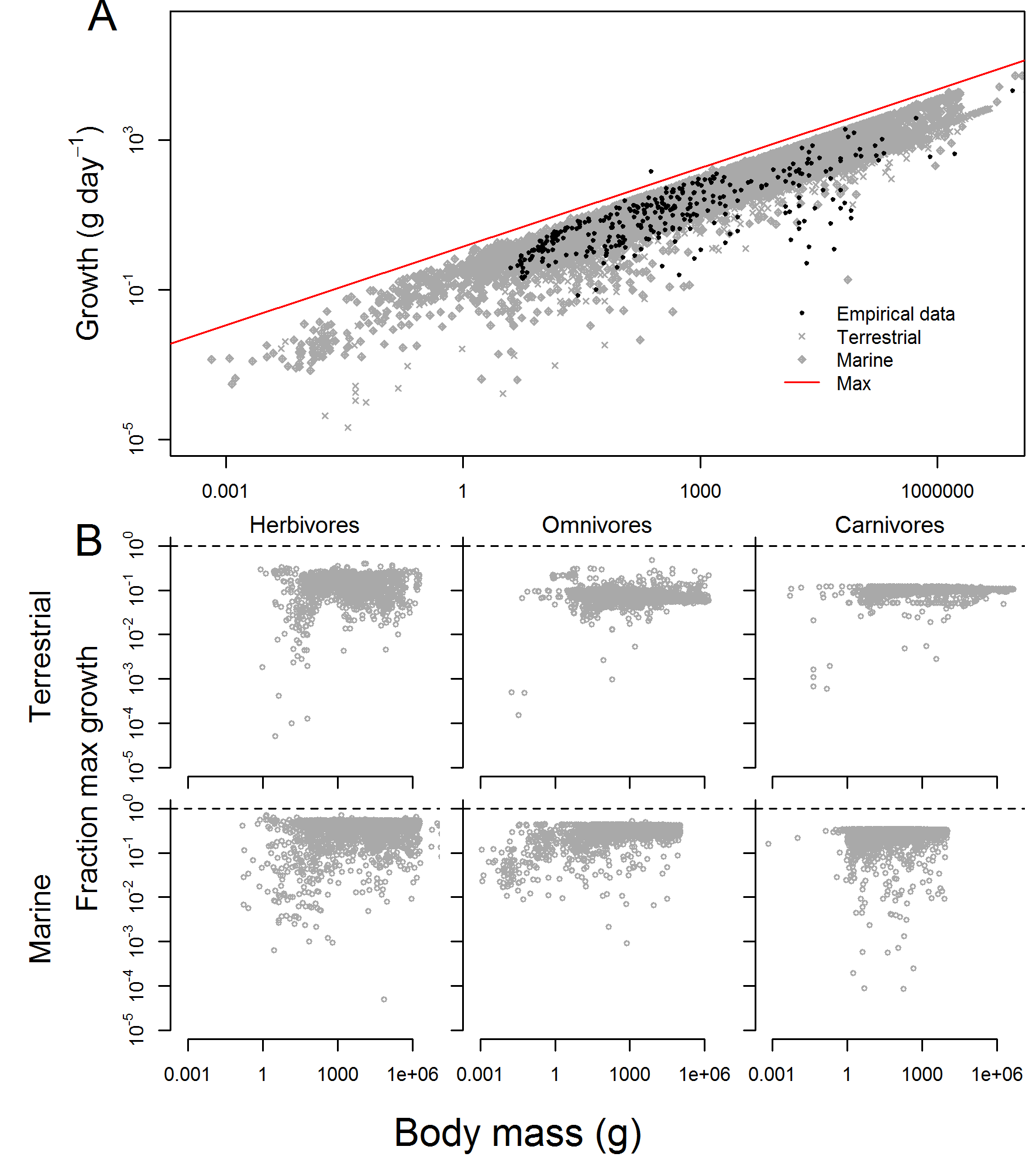

Supplement: Figure S3 — Emergent model growth rates compared to theoretical maximum rates. (A) Absolute emergent model growth rate (grey crosses and diamonds) relationship with body mass compared with empirical (black points) and theoretical maximum (red line). (B) The relationship between emergent model growth rate as a fraction of theoretical maximum growth rate (grey open circles) and body mass for each trophic level in terrestrial or marine cells. Modelled emergent individual-level properties are sampled from 100-y model runs for the four focal grid cells (Table 4). (TIFF) [file pbio.1001841.s003.tiff]

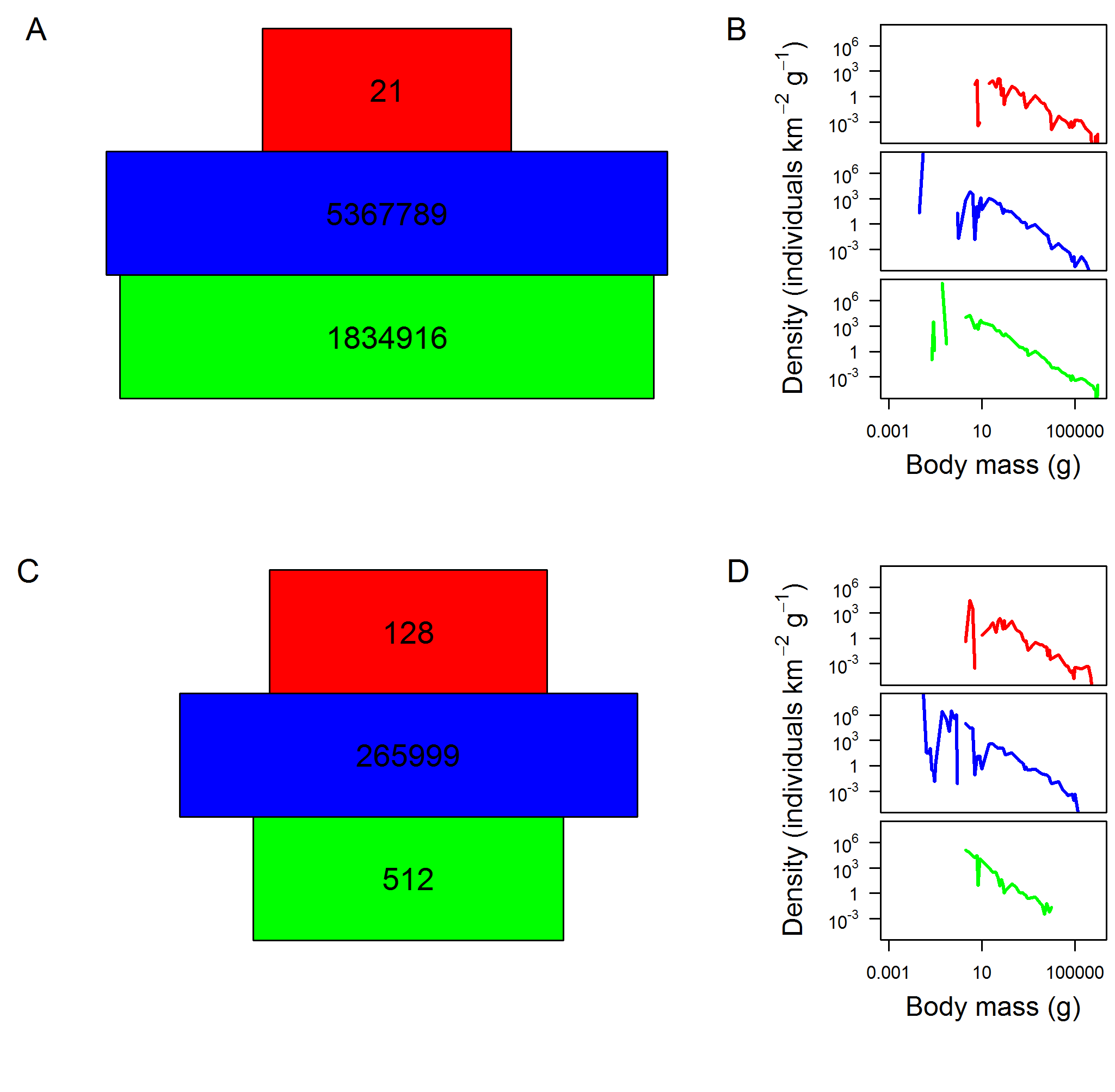

Supplement: Figure S4 — Trophic abundance pyramids. Community-level abundance pyramids across all cohorts belonging to each trophic level emergent from the model for an example of terrestrial and marine grid cell (grid cells T1 and M1 from Table 4). Results are from the final year of a 100-y model run. Light green represents herbivores, blue represents omnivores, and red represents carnivores. Total abundance densities (1,000 s individuals/km2) are indicated by the widths (after log-transformation) and numbers within the boxes. (TIF) [file pbio.1001841.s004.tiff]

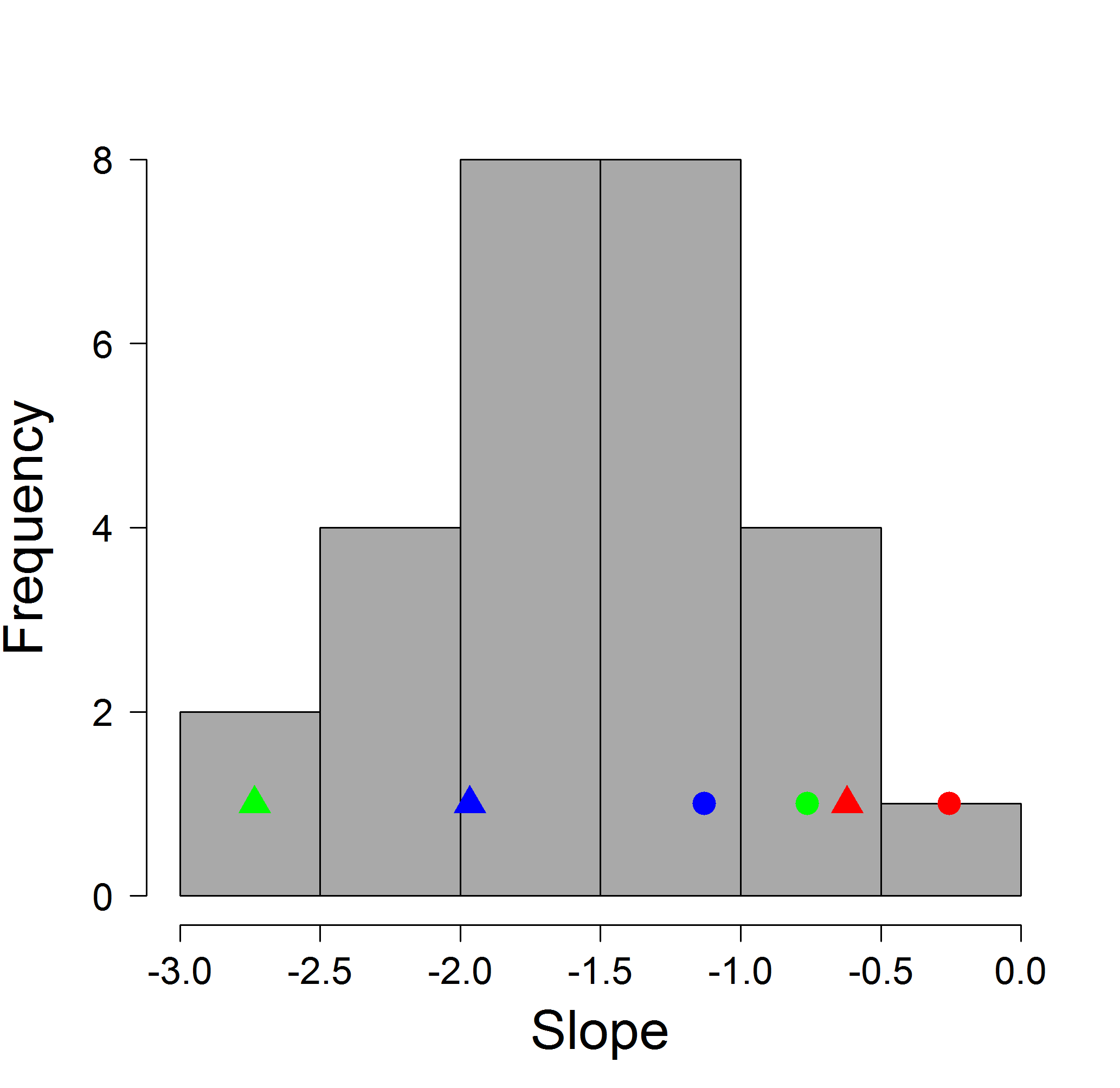

Supplement: Figure S5 — Comparison of model predicted with empirical normalised body mass spectra (NBS). Frequency distribution of the slope of NBS from [75] with model-derived NBS slope values, calculated following Sprules and Munawar [114], for carnivores (red), omnivores (blue), and herbivores (green). Triangles correspond to slopes for the low productivity, aseasonal marine cell (M1, Table 4) and circles to the high productivity, aseasonal terrestrial cell (T1, Table 4). (TIF) [file pbio.1001841.s005.tiff]

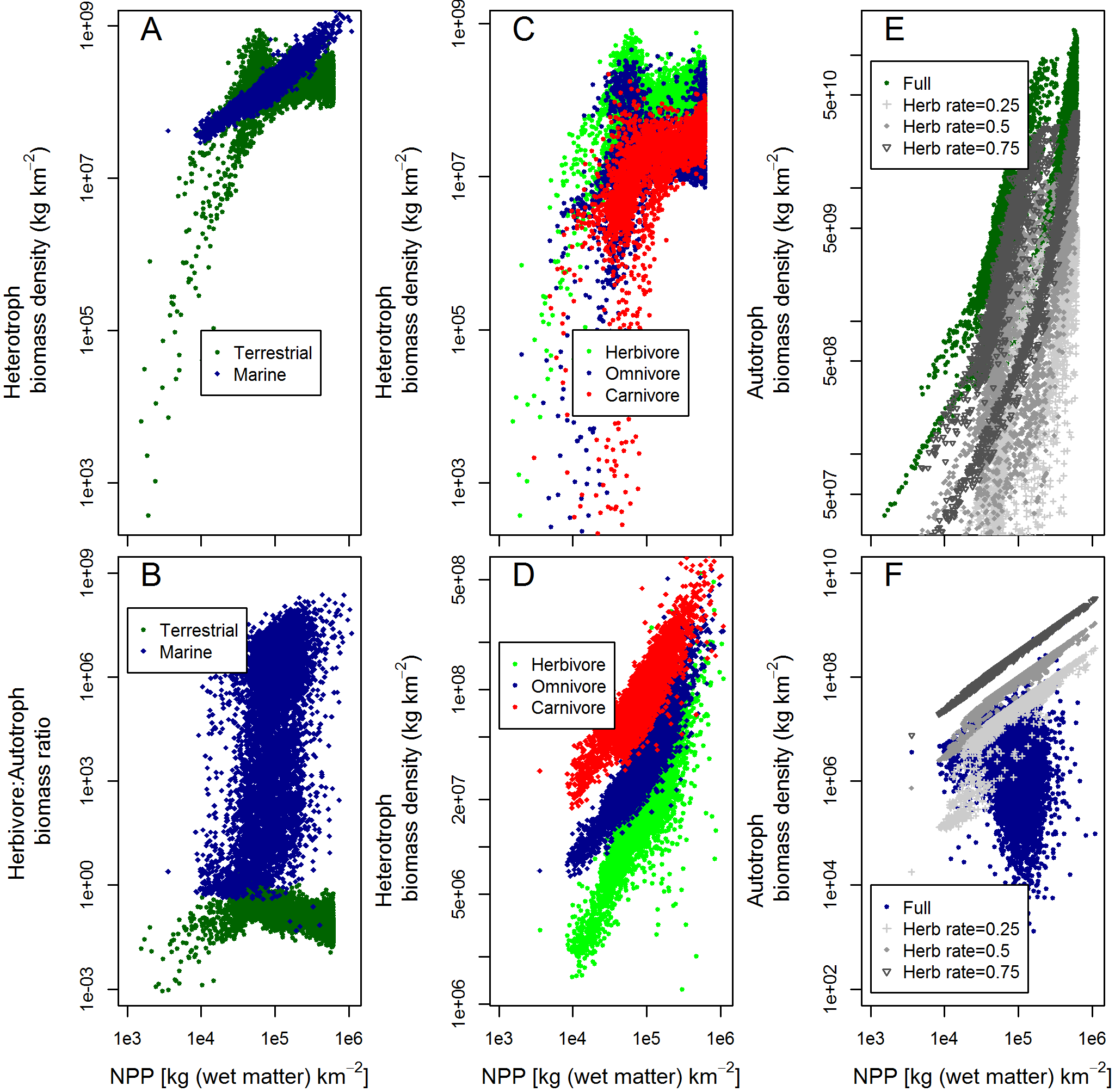

Supplement: Figure S6 — Relationships between predicted biomass densities and NPP. The global relationship between total heterotrophic biomass and NPP split between terrestrial and marine realms (A). The global relationship between the ratio of herbivore to autotroph biomasses and NPP split between terrestrial and marine realms (B). The relationships between different trophic levels and NPP across terrestrial (C) and marine (D) environments. The relationship between autotroph biomass and NPP across terrestrial (E) and marine environments (F) with heterotrophs modelled explicitly “full” and constant proportional autotroph herbivory loss rates of 0.25, 0.5, and 0.75. (TIFF) [file pbio.1001841.s006.tiff]

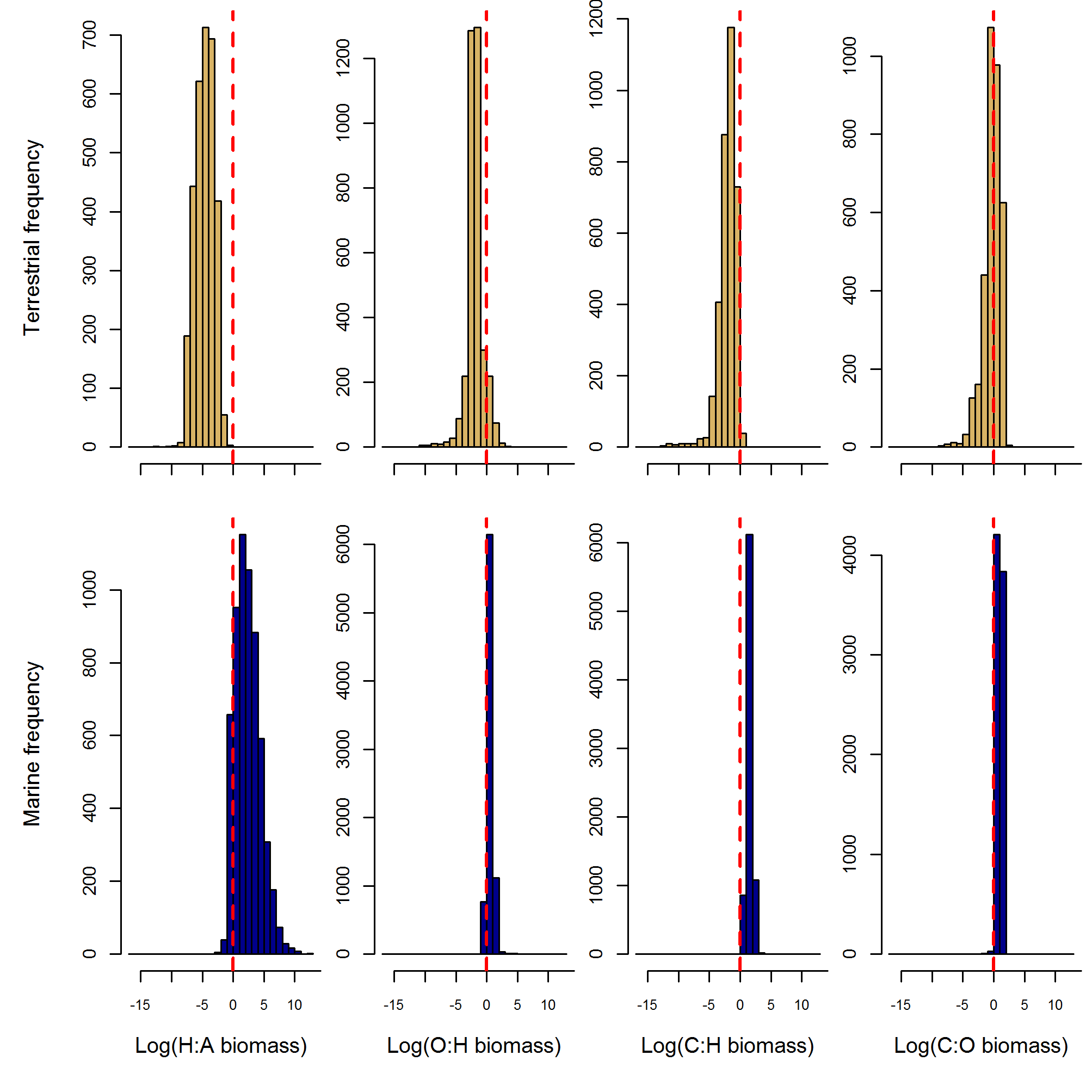

Supplement: Figure S7 — Frequency distributions of trophic biomass structure. Frequency distributions of log-transformed ratios of trophic-level biomasses in terrestrial grid cells (brown) and marine grid cells (blue), for H∶A = herbivore to autotroph, O∶H = omnivore to herbivore, C∶H = carnivore to herbivore, and C∶O = carnivore to omnivore biomass ratio. Red dashed lines indicate where the biomass ratio equals 1.0, which means equality of the two trophic-level biomasses. (TIFF) [file pbio.1001841.s007.tiff]

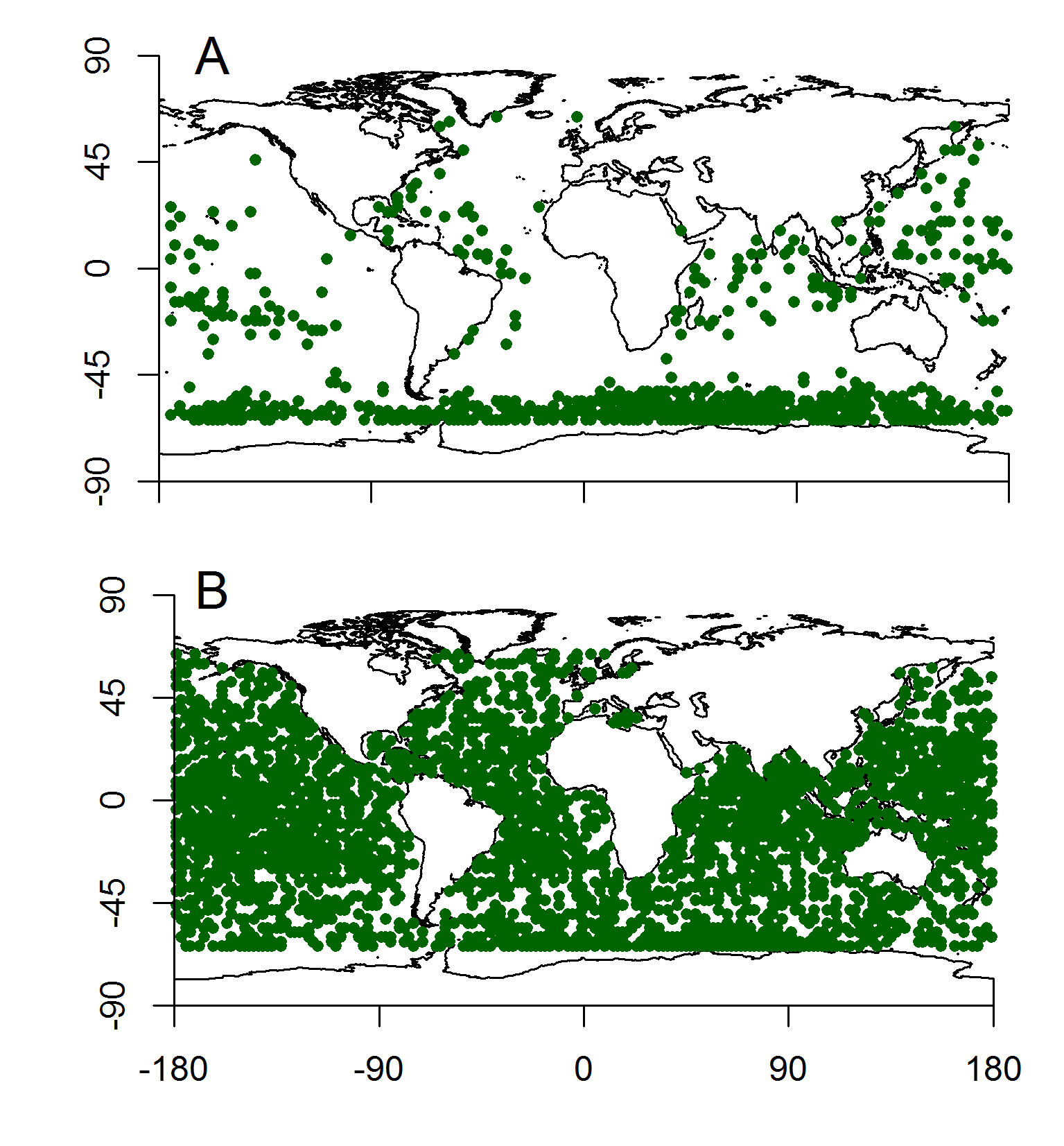

Supplement: Figure S8 — Spatial extent of un-inverted marine trophic structure for the bottom two trophic levels: herbivores and autotrophs. Spatial locations (green points) of un-inverted herbivores to autotroph trophic structure (i.e., where there is less herbivore than autotroph biomass) in (A) a simulation where dispersal was permitted (Study 4, Table 3) and (B) when dispersal is not modelled. (TIFF) [file pbio.1001841.s008.tiff]

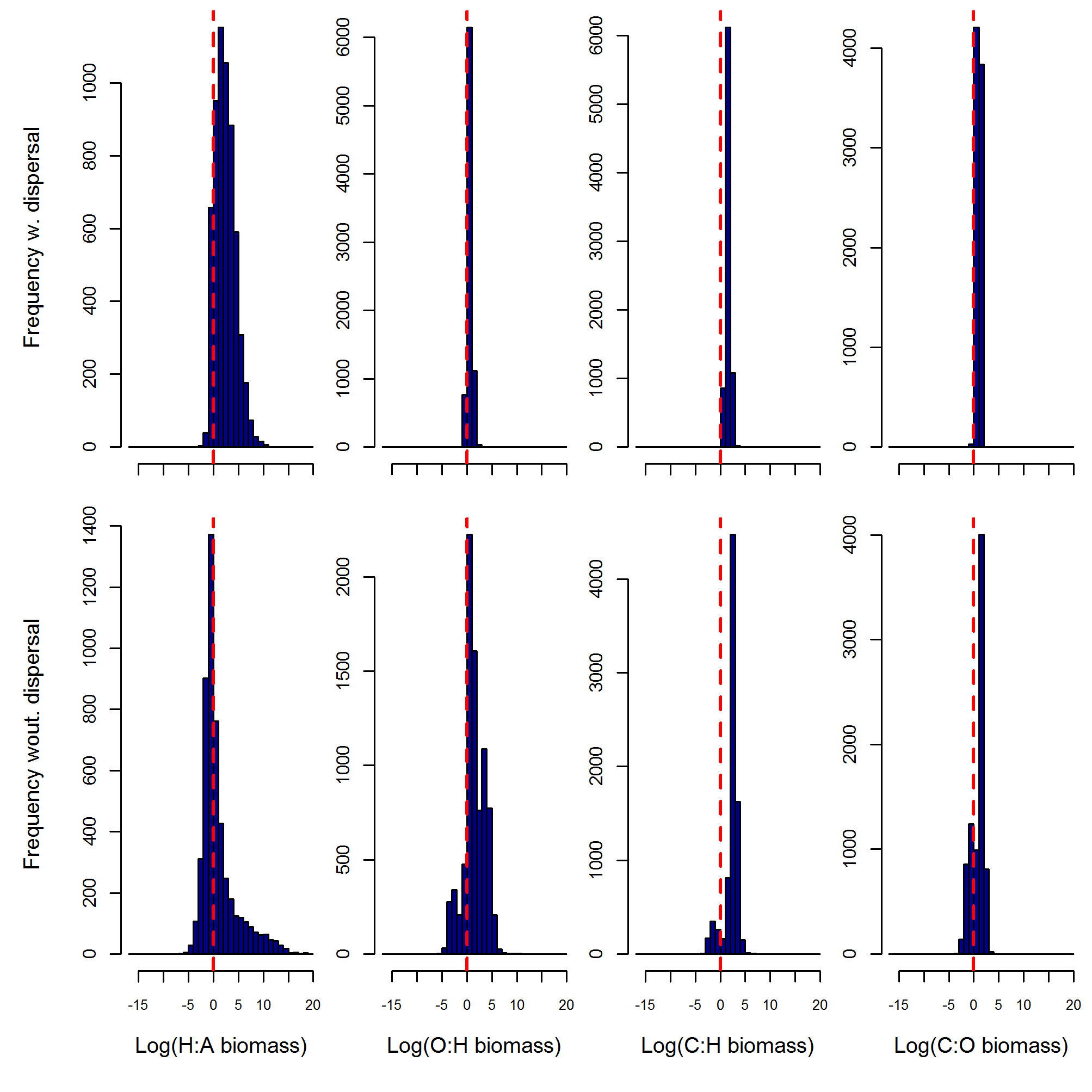

Supplement: Figure S9 — Frequency distribution of marine trophic structure in the absence of dispersal. Frequency distributions of log-transformed ratios of trophic-level biomasses in marine grid cells with dispersal (upper set of histograms—Study 4, Table 3) and marine grid cells without any dispersal modelled (lower set of histograms). H∶A, herbivore to autotroph; O∶H, omnivore to herbivore; C∶H, carnivore to herbivore; C∶O, carnivore to omnivore biomass ratio. Red dashed lines indicate where the biomass ratio equals 1.0, which means equality of the two trophic-level biomasses. (TIFF) [file pbio.1001841.s009.tiff]

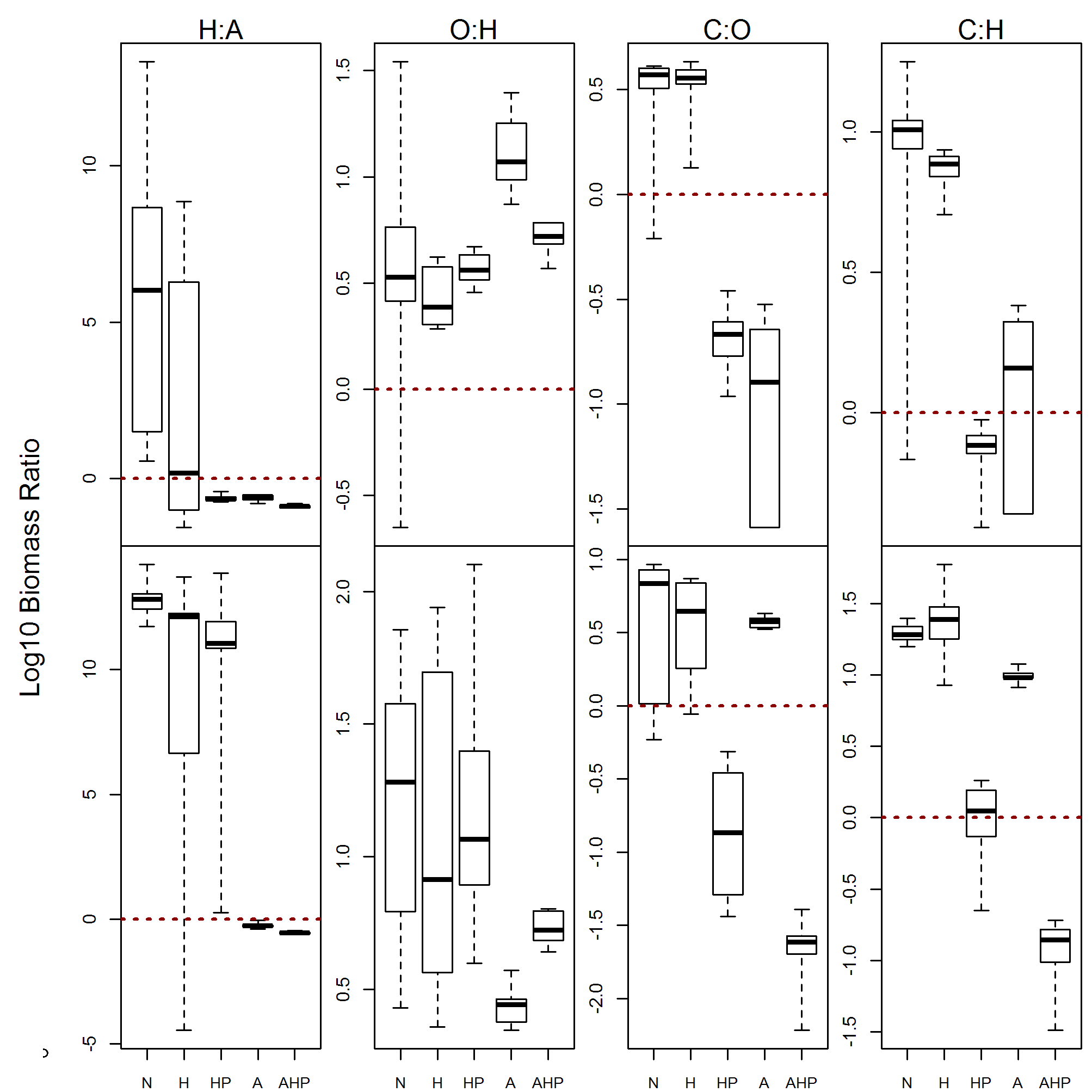

Supplement: Figure S10 — The effects of turnover rates and trophic transfer efficiencies on marine trophic structure. Box and whisker plots of the predicted ratios of trophic levels (H∶A, herbivore to autotroph; O∶H, omnivore to herbivore; C∶H, carnivore to herbivore; C∶O, carnivore to omnivore biomass ratio) for ensembles of 10 replicate simulations with different model assumptions investigating the mechanisms giving rise to inverted marine trophic biomass structure: N, the full model for a single grid cell; H, herbivore assimilation efficiency reduced to 20% (from 60–70% omnivore–herbivore); HP, herbivore and predator assimilation efficiency reduced to 20% (from 60–80% omnivore–carnivore); A, attack rates of herbivores and predators decreased by two orders of magnitude; AHP, combined reduction of attack rates, herbivore assimilation, and predator assimilation as above. Dark bars indicate median values, boxes the interquartile ranges, and whiskers the maximal range. Upper panels correspond to grid cell M1 and lower panels to grid cell M2 (Table 4). (TIFF) [file pbio.1001841.s010.tiff]

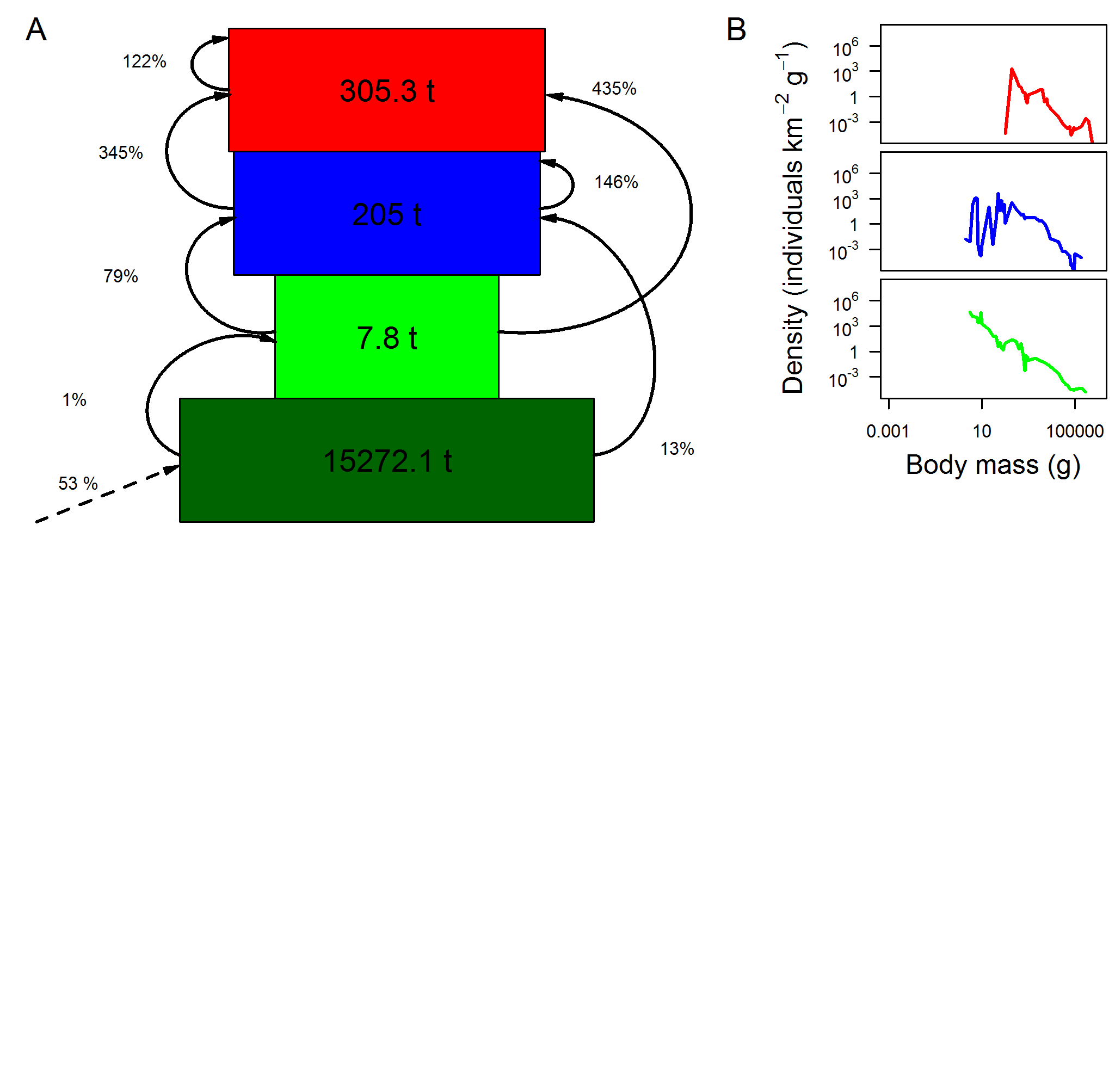

Supplement: Figure S11 — Community-level properties for cell T1 with all edible plant matter available for herbivory. Trophic pyramid and size distribution spectra for focal cell T1 with the value of parameter equal to 1 for terrestrial herbivores, which means that each terrestrial herbivore cohort experiences 100% of the edible plant matter in the grid cell when it is eating. The release of this parameter does not affect the low herbivore to primary producer ratio for terrestrial communities. Here, the ratio is 1.0%, marginally higher than that calculated for the same location using a value for of 10% (Figure 4). (TIFF) [file pbio.1001841.s011.tiff]
