# Supplementary material for: Emergent Global Patterns of Ecosystem Structure and Function from a Mechanistic General Ecosystem Model
Source: PLoS Biol. 2014 Apr 22;12(4):e1001841. doi: 10.1371/journal.pbio.1001841 (PMC3995663; doi:10.1371/journal.pbio.1001841)
Supplement: Table S1 — Environmental data sources. External global environmental data sources used within the model. Units represent those used within the Madingley model, not those of the original source data. a Environmental variables were long-term/multidecadal average values. (DOCX) [file pbio.1001841.s012.docx]

Supplementary Material: Emergent global patterns of ecosystem structure and function from a mechanistic General Ecosystem Model

Running head: A mechanistic general model of global ecosystems

Harfoot, M. B. J.^1,2^*^,†^, Newbold T.^1,2^*, Tittensor, D. P.^1,2,3^*, Emmott, S.^2^, Hutton, J.^1^, Lyutsarev, V. ^2^, Smith, M. J.^2^, Scharlemann, J. P. W.^1,4^, Purves, D. W.^2^

^1^ United Nations Environment Programme World Conservation Monitoring Centre, Cambridge, CB3 0DL, UK

^2^ Microsoft Research Computational Science Laboratory, Cambridge, CB1 2FB, UK

^3^ Dalhousie University, Halifax, NS, B3H 4R2, Canada

^4^ School of Life Sciences, University of Sussex, Falmer, Brighton, BN1 9QG, UK

^*^ These authors contributed equally to this work

^†^ Email: mike.harfoot@unep-wcmc.org

# Table S1. Environmental data sources

| **Data layer** | **Description** | **Time period** | **Time step** | **Source** | **Units** | **Model realm** | **Used for** |
| --- | --- | --- | --- | --- | --- | --- | --- |
| Land-sea mask | Land-Sea Mask from NASA International Satellite Land Surface Climatology Project | N/A | N/A | [1,2] | N/A | Both | Allocation of cell to realm |
| Near surface ocean temperature | Near surface ocean temperature | 1961-1990 | Monthly^a^ | [3] | °C | Both | In conjunction with land-sea mask for allocation of cells realm |
| Near surface air temperature | Near surface air temperature | 1961-1990 | Monthly^a^ | [3] | °C | Both | Temperature dependent ecological processes |
| Precipitation | Precipitation | 1961-1990 | Monthly^a^ | [3] | mm | Terrestrial | Modelling terrestrial primary productivity |
| Available water capacity | Available soil water capacity | 1960-2000 | Constant | [4] | mm | Terrestrial | Modelling terrestrial primary productivity |
| Number of frost days | Number of frost days | 1961-1990 | Monthly^a^ | [3] | Days | Terrestrial | Modelling terrestrial primary productivity |
| Terrestrial net primary productivity | MODIS derived net primary productivity | 2005 | Monthly | [5] | gC m^-2­^ day^-1^ | Terrestrial | Seasonality of modelled NPP |
| Diurnal air temperature range | Diurnal air temperature range over land | 1961-1990 | Monthly^a^ | [3] | °C | Terrestrial | Calculating temperature limitation of ectotherm activity |
| Sea surface temperature | Sea surface temperature | 1958-2001 | Monthly^a^ | [6] | °C | Marine | Temperature dependent ecological processes |
| Ocean net primary productivity | Net primary productivity via Vertically Generalized Production Model | 2003-2011 | Monthly^a^ | [7,8] | gCm^-2^day^-1^ | Marine | Productivity of marine stocks (phytoplankton) |
| Ocean current velocity | Meridional and latitudinal current speeds | 1958-2001 | Monthly^a^ | [6] | cm s^-1^ | Marine | Drives advective dispersal |

External global environmental data sources used within the model. Units represent those used within the Madingley model, not those of the original source data. ^a^ indicates environmental variables were long-term/multi-decadal average values.

# References

1. Meeson BW, Corprew FE, McManus JMP, Myers DM, Closs JW, et al. (1995) ISLSCP Initiative I -- Global Data Sets for Land-Atmosphere Models,1987-1988. Volumes 1Ð5.

2. Sellers PJ, Meeson BW, Closs J, Collatz J, Corprew F, et al. (1995) An Overview of the ISLSCP Initiative I -- Global Data Sets. On: ISLSCP Initiative I Global Data Sets for Land-Atmosphere Models, 1987-1988. Volumes 1-5.

3. Microsoft Research Cambridge Computational Science Laboratory (2012) FetchClimate. Available: http://fetchclimate.cloudapp.net/. Accessed 11 October 2012.

4. ISRIC-WISE (2012) Global data set of derived soil properties on a 0.5 by 0.5 degree grid (ver. 3.0). Available: http://www.isric.org/data/isric-wise-global-data-set-derived-soil-properties-05-05-degree-grid-ver-30. Accessed 11 October 2012.

5. National Aeronautics and Space Administration (2012) NASA Earth Observations: Net Primary Productivity. Available: http://neo.sci.gsfc.nasa.gov/Search.html?group=53. Accessed 11 October 2012.

6. Carton JA, Giese BS (2008) A reanalysis of ocean climate using Simple Ocean Data Assimilation (SODA). Mon Weather Rev 136: 2999–3017. doi:10.1175/2007MWR1978.1.

7. Behrenfeld M, Falkowski P (1997) Photosynthetic rates derived from satellite-based chlorophyll concentration. Limnol Oceanogr 42: 1–20.

8. Rutgers Institute of Marine and Coastal Sciences (2004) Vertically Generalized Production Model Estimates of Global Primary Production. Available: http://marine.rutgers.edu/opp/Production/Production1.html. Accessed 11 October 2012.
