# Supplementary material for: Emergent Global Patterns of Ecosystem Structure and Function from a Mechanistic General Ecosystem Model
Source: PLoS Biol. 2014 Apr 22;12(4):e1001841. doi: 10.1371/journal.pbio.1001841 (PMC3995663; doi:10.1371/journal.pbio.1001841)
Supplement: Table S2 — Model parameters and their values. (DOCX) [file pbio.1001841.s013.docx]

Supplementary Material: Emergent global patterns of ecosystem structure and function from a mechanistic General Ecosystem Model

Running head: A mechanistic general model of global ecosystems

Harfoot, M. B. J.^1,2^*^,†^, Newbold T.^1,2^*, Tittensor, D. P.^1,2,3^*, Emmott, S.^2^, Hutton, J.^1^, Lyutsarev, V. ^2^, Smith, M. J.^2^, Scharlemann, J. P. W.^1,4^, Purves, D. W.^2^

^1^ United Nations Environment Programme World Conservation Monitoring Centre, Cambridge, CB3 0DL, UK

^2^ Microsoft Research Computational Science Laboratory, Cambridge, CB1 2FB, UK

^3^ Dalhousie University, Halifax, NS, B3H 4R2, Canada

^4^ School of Life Sciences, University of Sussex, Falmer, Brighton, BN1 9QG, UK

^*^ These authors contributed equally to this work

^†^ Email: mike.harfoot@unep-wcmc.org

# Table S2. Model parameters and their values

| **Model component** | **Parameter** | **Description** | **Units** | **Value** | **Source** |
| --- | --- | --- | --- | --- | --- |
| ***Terrestrial plant model*** | *ψ* | Conversion factor from kg C to plant wet matter in grams | g (kgC)^-1^ | 9.86 | Derived using data from [1] |
|  | $f_{Struct}^{max}$ | Maximum allowable value of fractional allocation of primary production to structural tissue | - | 3.63 × 10^-1^ | [2] |
|  | $f_{Struct}^{min}$ | Minimum allowable value of fractional allocation of primary production to structural tissue | - | 0.01 | [2] |
|  | $\varphi_{f_{struct}}$ | Coefficient relating fractional allocation to structural tissue to NPP | [(kgC)m^-2^yr^-1^]^-1^ | 7.15 | [2] |
|  | *m_e_* | Slope of the linear relationship between temperature and the mortality of evergreen leaves | yr^-1^ °C^-1^ | 4.03 × 10^-2^ | [2] |
|  | *m_d_* | Slope of the linear relationship between temperature and the mortality of deciduous leaves | yr^-1^ °C^-1^ | 2.06 × 10^-2^ | [2] |
|  | *m_f_* | Slope of the linear relationship between temperature and the mortality of fine roots | yr^-1^ °C^-1^ | 4.31 × 10^-2^ | [2] |
|  | *c_e_* | Intercept of the linear relationship between temperature and the mortality of evergreen leaves | yr^-1^ | 1.01 | [2] |
|  | *c_d_* | Intercept of the linear relationship between temperature and the mortality of deciduous leaves | yr^-1^ | -1.20 | [2] |
|  | *c_f_* | Intercept of the linear relationship between temperature and the mortality of fine roots | yr^-1^ | -1.48 | [2] |
|  | $a_{f_{ever}}$ | Relates fractional allocation of productivity to evergreen plant matter | - | 1.27 | [2] |
|  | $b_{f_{ever}}$ | Relates fractional allocation of productivity to evergreen plant matter | - | -1.83 | [2] |
|  | $c_{f_{ever}}$ | Relates fractional allocation of productivity to evergreen plant matter | - | 8.45 × 10^-1^ | [2] |
|  | *c_p_* | Intercept for the linear exponent term describing the Miami model relationship between net primary production and temperature | - | 2.37 × 10^-1^ | [2] |
|  | *m_p_* | Slope of the linear exponent term relating net primary production to temperature | °C^-1^ | 1.01× 10^-2^ | [2] |
|  | *ρ* | Relates net primary production to total annual precipitation | mm^-1^ | 1.18 × 10^-3^ | [2] |
|  | ${NPP}_{max}$ | Maximum possible net primary production | (kgC) m^-2^ yr^-1^ | 9.62 × 10^-1^ | [2] |
| ***Marine primary productivity*** | *ξ* | Scalar to convert net marine primary productivity in carbon to total algal biomass | g (gC)^-1^ | 10.0 | [3] |
| ***Heterotroph eating*** | *τ_f_*_,_ | Proportion of time for which functional group is active | - | 0.5 | Own calculations |
|  | $\varepsilon_{f}^{herb}$ | Proportional herbivore assimilation efficiency | - | Functional group specific (see Table S2) | [4,5] |
|  | $\alpha_{0}^{herb}$ | Effective rate per unit body mass at which a herbivore searches its environment | ha day^-1^ gram^-1^ | 1 × 10^-11^ | Own calculations |
|  | $\phi_{herb,f}$ | Fraction of the total herbivore stock that is available to any one herbivore cohort | - | 0.1 (terrestrial functional groups); 1.0 (marine functional groups) | Own calculations |
|  | *b^herb^* | Exponent of the power-law function relating the handling time of autotroph matter to herbivore mass | - | 0.7 | Own calculations |
|  | $M_{ref}^{herb}$ | Reference mass for herbivore handling time (see next parameter for usage) | g | 1.0 | Own calculations |
|  | $h_{0}^{herb}$ | Time that it would take a herbivore of body mass equal to the reference mass,  $M_{ref}^{herb}$, to handle one gram of autotroph biomass | days | 0.7 | Own calculations |
|  | $\varepsilon_{f}^{pred}$ | Proportional carnivore assimilation efficiency | - | Functional group specific (see Table S2) | [4,5] |
|  | $\alpha_{0}^{pred}$ | The effective rate per unit body mass at which a predator searches its environment | ha day^‑1^ gram^-1^ | 1 × 10^-6^ | Own calculations |
|  | $b^{pred}$ | Exponent of the power-law relationship between the handling time of prey and the ratio of prey to predator body mass | - | 0.7 | Own calculations |
|  | $\sigma_{pred-prey}^{opt}$ | Standard deviation of the normal distribution describing realized attack rates around the optimal predator-prey body mass ratio | - | 0.7 | Own calculations |
|  | $\theta_{min,f}^{opt}$ | The minimum optimal prey-predator body mass ratio | - | 1.0 x10^-5^ (baleen whale functional groups); 0.01 (all other functional groups) | Own calculations |
|  | $\theta_{f}^{opt}$ | The mean optimal prey-predator body mass ratio, from which actual cohort optima are drawn | - | 0.01 (baleen whale functional groups); 0.1 (all other functional groups, both marine and terrestrial) | [6–8] |
|  | $\sigma_{f}^{opt}$ | The standard deviation of optimal predator-prey mass ratios among cohorts | - | 3 × 10^-3^ (baleen whale functional groups); 0.02 (all other functional groups); | Own calculations |
|  | $N_{\sigma_{pred-prey}^{opt}}$ | The standard deviations of the realized attack rates around the optimal predator-prey body mass ratio for which to calculate predator specific cumulative prey densities. |  | 3 |  |
|  | $h_{0}^{pred}$ | Time that it would take a predator of body mass equal to the reference mass, $M_{ref}^{pred}$, to handle a prey individual of body mass equal to one gram | days | 0.5 | Own calculations |
| ***Activity*** | $m_{tol, terrestrial}$ | Slope of the relationship between monthly temperature variability and the upper critical temperature limit relative to annual mean temperature, for terrestrial ectothermic functional groups | - | 1.6 | [9] |
|  | $c_{tol,terrestrial}$ | Intercept of the relationship between monthly temperature variability and the upper critical temperature limit relative to annual mean temperature, for terrestrial ectothermic functional groups | °C | 6.61 | [9] |
|  | $m_{tsm}$ | Slope of the relationship between monthly temperature variability and the optimal temperature relative to annual mean temperature, for terrestrial ectothermic functional groups | - | 1.53 | [9] |
|  | $c_{tsm}$ | Intercept of the relationship between monthly temperature variability and the optimal temperature relative to annual mean temperature, for terrestrial ectothermic functional groups | °C | 1.51 | [9] |
| ***Metabolism*** | $I_{0,f}^{FMR}$ | Mass- and temperature-independent metabolic rate constants for field metabolic rates | $\mathrm{eV}g^{-b^{metab,FMR}}$ | 9.08 × 10^11^ (endothermic functional groups); 1.49 × 10^11^ (ectothermic functional groups) | [10] |
|  | $I_{0,f}^{BMR}$ | Mass- and temperature-independent metabolic rate constants for basal metabolic rates | $\mathrm{eV}g^{-b^{metab,BMR}}$ | 4.19 × 10^10^ | [11] |
|  | $E_{A}$ | Aggregate activation Energy of metabolic reactions | eV | 0.69 | [11] |
|  | $b_{f}^{metab, FMR}$ | Body mass exponents for field metabolic rates | - | 0.7 (endothermic functional groups); 0.88 (ectothermic functional groups) | [10] |
|  | $b^{metab, BMR}$ | Body mass exponents for basal metabolic rates | - | 0.69 | [11] |
|  | $E_{S}$ | Scalar to convert energy in kJ to energy in grams body mass | g kJ^-1^ | 3.7 × 10^-2^ | Own calculation using [12] |
| ***Reproduction*** | $\beta^{repro}$ | Threshold ratio of total body mass to adult body mass above which reproductive events are assumed to occur | - | 1.5 | Own calculations |
|  | $\sigma^{J}$ | When evolution occurs, standard deviation of the normal distribution describing an offspring cohort’s juvenile mass around its parent cohort's juvenile mass | g | 0.05 | Own calculations |
|  | $\sigma^{A}$ | When evolution occurs, standard deviation of the normal distribution describing an offspring cohort’s adult mass around its parent cohort's adult mass | g | 0.05 | Own calculations |
|  | $\chi$ | Proportion of current body mass assigned to reproduction during semelparous reproduction | - | 0.5 | Own calculations |
| ***Non-predation mortality*** | *μ_bg_* | Instantaneous fractional rate of background mortality | day^-1^ | 1× 10^-3^ | Own calculations |
|  | $\lambda_{se}$ | Instantaneous fractional rate of senescence mortality for an individual at the point of maturity | day^-1^ | 3 × 10^-3^ | Own calculations |
|  | $\lambda_{max}$ | Maximum possible fractional rate of starvation mortality per day | day^-1^ | 1 | Own calculations |
|  | $\vartheta_{st}$ | The inflection point of the logistic function describing the ratio of the realized starvation mortality rate to the maximum starvation mortality rate | - | 0.6 | Own calculations |
|  | $\zeta_{st}$ | The scaling parameter for the logistic function describing the ratio of the realized starvation mortality rate to the maximum starvation mortality rate | - | 0.05 | Own calculations |
| ***Dispersal*** | $M_{ref}^{disp}$ | Diffusive dispersal reference mass | g | 1 | N/A |
|  | $\nu_{disp}$ | Diffusive dispersal speed of an individual of mass equal to the reference mass | km month^-1^ | 2.78 x10^-2^ | [13] |
|  | $o_{disp}$ | Exponent for the power law describing the scaling of dispersal distance with current individual body mass relative to the reference diffusive dispersal mass | - | 0.48 | [13] |
|  | $\beta_{density}^{responsive}$ | Mass-proportional density dependent threshold below which reproduction-related responsive dispersal is attempted | g km^-2^ | 5.0 x10^4^ | Own calculations |
|  | $\beta_{bodymass}^{responsive}$ | Ratio of adult body mass to mature mass below which starvation-related responsive dispersal is attempted | - | 0.8 | Own calculations |
| ***Other model processes*** | $\omega$ | Scales the minimum body mass specified for a functional group to establish a minimum adult mass for a cohort in that functional group | - | 50 | Own calculations |
|  | $\mathcal{a}_{Adult-Juv}$ | For seeding initial cohorts, the intercept term for the linear relationship between the expected log adult to juvenile mass ratio and adult mass | g | 2.24 (terrestrial); 2.5 (marine) | Own calculations |
|  | $\mathcal{b}_{Adult-Juv}$ | For seeding initial cohorts, the slope of the linear relationship between the expected log adult to juvenile mass ratio and adult mass | - | 0.13 (terrestrial); 0.2 (marine) | Own calculations |
|  | $\sigma_{Adult-Juv}$ | Standard deviation of the log normal distribution of Adult to Juvenile body mass ratios | g | 0.5 | Own calculations |
|  | $\beta^{extinct}$ | Abundance threshold below which a cohort is assumed to be destined for extinction and is removed from the model | - | 1 | Own calculations |
|  | $\mathbb{c}$ | Reference number of cohorts for which biomass relationship was established | cohort | 3.3 × 10^3^ | Own calculations |
|  | $\mathcal{w}$ | Scalar for the relationship describing initial cohort biomass density as a function of initial cohort body mass | g km^-2^ | 3 × 10^5^ | Own calculations, based on [14] |
|  | $\mathfrak{b}$ | Base of the exponential relationship describing initial cohort biomass density as a function of initial cohort body mass (the exponent) | - | 0.6 | Own calculations based on [14] |
| ***Mathematical constants*** | $\pi$ | Pi | - | 3.14 | [15] |
|  | $k_{B}$ | Boltzmann constant | eV K^-1^ | 8.62 × 10^-5^ | [16] |

# References

1. Kattge J, Díaz S, Lavorel S, Prentice IC, Leadley P, et al. (2011) TRY - a global database of plant traits. Glob Chang Biol 17: 2905–2935. doi:10.1111/j.1365-2486.2011.02451.x.

2. Smith MJ, Vanderwel MC, Lyutsarev V, Emmott S, Purves DW (2012) The climate dependence of the terrestrial carbon cycle; including parameter and structural uncertainties. Biogeosciences Discuss 9: 13439–13496.

3. Strickland JDH (1966) Measuring the production of marine phytoplankton. Bulletin No. 122. Ottawa, Canada: Fisheries Research Board of Canada.

4. Sterner RW, Hessen DO (1994) Algal nutrient limitation and the nutrition of aquatic herbivores. Annu Rev Ecol Syst 25: 1–29.

5. Chapin FS, Matson PA, Mooney HA (2011) Principles of Terrestrial Ecosystem Ecology. 2nd Editio. New York, USA: Springer-Verlag.

6. Brose U, Cushing L, Berlow E, Jonsson T, Banasek-Richter C, et al. (2005) Body sizes of consumers and their resources. Ecology 86: 2545.

7. Williams RJ, Anandanadesan A, Purves DW (2010) The probabilistic niche model reveals the niche structure and role of body size in a complex food web. PLoS One 5: e12092. doi:10.1371/journal.pone.0012092.

8. Scharf FS, Juanes F, Rountree RA (2000) Predator size - prey size relationships of marine fish predators: interspecific variation and effects of ontogeny and body size on trophic-niche breadth. Mar Ecol Prog Ser 208: 229–248.

9. Deutsch CA, Tewksbury JJ, Huey RB, Sheldon KS, Ghalambor CK, et al. (2008) Impacts of climate warming on terrestrial ectotherms across latitude. Proc Natl Acad Sci U S A 105: 6668–6672.

10. Nagy KA, Girard IA, Brown TK (1999) Energetics of free-ranging mammals, reptiles, and birds. Annu Rev Nutr 19: 247–277.

11. Brown JH, Gillooly JF, Allen AP, Savage VM, West GB (2004) Toward a metabolic theory of ecology. Ecology 85: 1771–1789.

12. Merrill AL, Watt BK (1955) Energy Value of Foods - basis and derivation. Washington DC, USA.

13. Schlag ZR, North EW (2012) Lagrangian TRANSport model (LTRANS v.2) User’s guide. Cambridge, MD.

14. Silva M, Downing JA (1995) CRC Handbook of Mammalian Body Masses. Boca Raton, Florida, USA: CRC Press.

15. Microsoft (n.d.) Math.Pi field. MSDN. Available: http://msdn.microsoft.com/en-us/library/system.math.pi.aspx.

16. NIST (n.d.) Boltzmann constant in eV/K. NIST Ref Constants, Units Uncertain. Available: http://physics.nist.gov/cgi-bin/cuu/Value?tkev|search_for=boltzmann.
