# Supplementary material for: Emergent Global Patterns of Ecosystem Structure and Function from a Mechanistic General Ecosystem Model
Source: PLoS Biol. 2014 Apr 22;12(4):e1001841. doi: 10.1371/journal.pbio.1001841 (PMC3995663; doi:10.1371/journal.pbio.1001841)
Supplement: Table S3 — Empirical estimates of trophic-level biomasses in globally widespread ecosystems in both marine and terrestrial environments. Notes: 1. Consider only the pelagic producers. Assume that dissolved organic carbon (DOC) and suspended particulate organic carbon (POC) are available to the pelagic community. Do not include benthic suspension feeders. Assume micro-zooplankton are herbivores (as defined in our model). For omnivores and carnivores, assume that the proportion of biomass that is supported originally by pelagic primary production is equal to the proportional rate of consumption of pelagically derived foods relative to consumption of foods from all sources. 2. From Table 12.2 (plants taken as above ground vascular + Moss + Algae + Lichens) of Chapin et al [115]. 3. Herbivore biomass taken from [73]. 4. Producer and herbivore biomasses from [73]. 5. Producer biomass of 9,999 g C m-2 comes from Figure 8 of Frangi and Lugo [116]. Montane palm floodplain forest; herbivore biomass taken from [73]. (DOCX) [file pbio.1001841.s014.docx]

Supplementary Material: Emergent global patterns of ecosystem structure and function from a mechanistic General Ecosystem Model

Running head: A mechanistic general model of global ecosystems

Harfoot, M. B. J.^1,2^*^,†^, Newbold T.^1,2^*, Tittensor, D. P.^1,2,3^*, Emmott, S.^2^, Hutton, J.^1^, Lyutsarev, V. ^2^, Smith, M. J.^2^, Scharlemann, J. P. W.^1,4^, Purves, D. W.^2^

^1^ United Nations Environment Programme World Conservation Monitoring Centre, Cambridge, CB3 0DL, UK

^2^ Microsoft Research Computational Science Laboratory, Cambridge, CB1 2FB, UK

^3^ Dalhousie University, Halifax, NS, B3H 4R2, Canada

^4^ School of Life Sciences, University of Sussex, Falmer, Brighton, BN1 9QG, UK

^*^ These authors contributed equally to this work

^†^ Email: mike.harfoot@unep-wcmc.org

# Table S3. Empirical estimates of trophic level biomasses in globally widespread ecosystems in both marine and terrestrial environments

| **Name** | **Realm** | **Ecosystem type** | **Lat** | **Long** | **Location notes** | **PB (gCm^-2^)** | **HB (gCm^-2^)** | **OB (gCm^-2^)** | **CB (gCm^-2^)** | **Reference** | **Notes** |
| --- | --- | --- | --- | --- | --- | --- | --- | --- | --- | --- | --- |
| Santa Monica Bay, California | Marine | Coastal | 33.75 | -118.80 |  | 1.27 | 0.10 |  |  | [1] | |
| Chesapeake Bay, United States | Marine | Coastal | 37.00 | -75.96 | Chesapeake Bay | 3.48 | 0.35 | 1.68 | 0.15 | [2] | 1 |
| Ythan estuary, Scotland | Marine | Estuary | 57.33 | -1.95 | Estuary - Ythan. Longitude coordinate in paper is wrong, updated using [3] | 0.75 | 0.14 | 0.12 | 0.19 | [4] | 1 |
| Ems estuary, Netherlands | Marine | Estuary | 53.43 | 6.90 | Estuary - Ems. | 0.90 | 0.09 | 0.07 | 0.03 | [4] | 1 |
| Kromme estuary, South Africa | Marine | Estuary | -34.13 | 24.85 | Estuary - Kromme | 0.41 |  | 0.18 | 0.31 | [4] | 1 |
| Swartkops estuary, South Africa | Marine | Estuary | -32.87 | 25.65 | Estuary - Swartkops | 3.73 | 0.00 | 6.12 | 0.55 | [4] | 1 |
| Barrow, Alaska | Terrestrial | Tundra | 71.30 | -156.77 |  | 75.27 | 0.12 |  | 0.00 | [5] | 2 |
| Andrews Experimental Forest, Oregon | Terrestrial | Temperate Forest | 44.00 | -122.50 |  | 26862.80 | 0.45 |  |  | [6] | 3 |
| Andrews Experimental Forest, Oregon | Terrestrial | Temperate Forest | 44.00 | -122.50 |  | 32528.80 | 0.67 |  |  | [6] | 3 |
| Andrews Experimental Forest, Oregon | Terrestrial | Temperate Forest | 44.00 | -122.50 |  | 20017.60 | 0.67 |  |  | [6] | 3 |
| Andrews Experimental Forest, Oregon | Terrestrial | Temperate Forest | 44.00 | -122.50 |  | 22590.40 | 0.67 |  |  | [6] | 3 |
| Andrews Experimental Forest, Oregon | Terrestrial | Temperate Forest | 44.00 | -122.50 |  | 39300.40 | 0.67 |  |  | [6] | 3 |
| San Simon Valley, Arizona | Terrestrial | Desert | 31.92 | -109.14 | Approximate | 184.00 | 0.06 |  |  | [7] | 4 |
| Cedar Creek Natural History Area, Minnesota | Terrestrial | Savannah | 45.41 | -93.20 |  | 17.74 | 0.10 |  |  | [8] | 3 |
| Devon Island, Nunavut | Terrestrial | Tundra | 75.13 | -87.85 | Approximate for island | 57.20 | 0.02 |  |  | [9] | |
| Devon Island, Nunavut | Terrestrial | Tundra | 75.13 | -87.85 | Approximate for island | 399.20 | 0.08 |  |  | [9] | |
| AEC Reservation, Oak Ridge, Tennessee | Terrestrial | Deciduous Forest | 35.93 | -84.32 |  | 8011.00 | 0.10 |  | 0.03 | [10] | From Figure 1 |
| Oak Ridge, Tennessee | Terrestrial | Grassland | 35.93 | -84.32 |  | 56.88 | 0.27 |  |  | [11] | Arthropods only |
| Luquillo Experimental Forest Biosphere Reserve, Puerto Rico | Terrestrial | Tropical Forest | 18.42 | -65.33 | Approximate | 9999.00 | 1.31 |  |  | [12] | 5 |
| Serengeti | Terrestrial | Savannah | -2.33 | 34.57 | Approximate. Note that there are 4 sites spread over about 1 degree in lat/long; not sure which site is which. | 33.47 | 0.73 |  |  | [13] | 3 |
| Serengeti | Terrestrial | Savannah | -2.33 | 34.57 | Approximate. Note that there are 4 sites spread over about 1 degree in lat/long; not sure which site is which. | 40.00 | 0.85 |  |  | [13] | 3 |
| Serengeti | Terrestrial | Savannah | -2.33 | 34.57 | Approximate | 116.56 | 0.34 |  |  | [14] | |

Notes:

1. Consider only the pelagic producers. Assume that DOC and suspended POC are available to the pelagic community. Don’t include benthic suspension feeders. Assume micro-zooplankton are herbivores (as defined in our model). For omnivores and carnivores. Assume that the propotion of biomass that is supported originally by pelagic primary production is equal the proportional rate of consumption of pelagically derived foods relative to consumption of foods from all sources.
2. From Table 12.2 (plants taken as above ground vascular + Moss + Algae + Lichens)
3. Herbivore biomass taken from [15]
4. Producer and Herbivore biomasses from [15]
5. Producer biomass of 9999 g C m-2 comes from Figure 8. Montane palm floodplain forest; Herbivore biomass taken from [15]

# Supporting Material References

1. Small LF, Landry MR, W ER, Azam F, Carlucci AF (1989) Role of plankton in the carbon and nitrogen budgets of Santa Monica Basin , California. Mar Ecol Prog Ser 56: 57–74.

2. Baird D, Ulanowicz RE (1989) The seasonal dynamics of the Chesapeake Bay ecosystem. Ecol Monogr 59: 329–364.

3. JNCC (n.d.) Ythan Estuary, Sands of Forvie and Meikle Loch. Available: http://jncc.defra.gov.uk/. Accessed 5 May 2013.

4. Baird D, E R (1993) Comparative study on the trophic structure , cycling and ecosystem properties of four tidal estuaries. Mar Ecol Prog Ser 99: 221–237.

5. Chapin FS, Miller PC, Billings WD, I CP (1980) Carbon and Nutrient Budgets and Their Control in Coastal Tundra. In: Brown J, Miller PC, Tieszen LL, Bunnell FL, editors. An Arctic Ecosystem: the Coastal Tundra at Barrow, Alaska. Stroudsburg, PA: Dowden, Hutchinson & Ross, Inc., Vol. 7. pp. 458–482.

6. Grier CC, Logan, Robert S (1977) Old-growth Pseudotsuga menziesii Communities of a Western Oregon Watershed: Biomass distribution and production budgets. Ecol Monogr 47: 373–400.

7. Chew RM, Chew AE (1970) Energy Relationships of the Mammals of a Desert Shrub ( Larrea tridentata ) Community. Ecol Monogr 40: 1–21.

8. Ritchie ME, Tilman D, Knops JMH (1998) Herbivore Effects on Plant and Nitrogen Dynamics in Oak Savanna. Ecology 79: 165–177. doi:10.1890/0012-9658(1998)079[0165:HEOPAN]2.0.CO;2.

9. Bliss LC, Courtin GM, Pattie DL, Riewe RR, Whitfield DWA, et al. (1973) Arctic Tundra Ecosystems. Annu Rev Ecol Syst 4: 359–399.

10. Harris WF, Sollins P, Edwards NT, Dinger BE, Shugart HH (1975) Analysis of carbon flow and productivity in a temperate deciduous forest ecosystem. In: Reichle D, Franklin J, Goodall D, editors. Washington DC: National Academy of Sciences. pp. 116–122.

11. Van Hook RI (1971) Energy and Nutrient Dynamics of Spider and Orthopteran Populations in a Grassland Ecosystem. Ecol Monogr 41: 1–26.

12. Frangi JL, Lugo AE (1985) Ecosystem Dynamics of a Subtropical Floodplain Forest. Ecol Monogr 55: 351–369.

13. McNaughton SJ (1985) Ecology of a Grazing Ecosystem : The Serengeti. Ecol Monogr 55: 260–294.

14. Sinclair ARE (1975) The resource limitation of trophic levels in Tropical Grassland Ecosystems. J Anim Ecol 44: 497–520.

15. Cebrian J, Shurin JB, Borer ET, Cardinale BJ, Ngai JT, et al. (2009) Producer nutritional quality controls ecosystem trophic structure. PLoS One 4: e4929. doi:10.1371/journal.pone.0004929.
