# Supplementary material for: Emergent Global Patterns of Ecosystem Structure and Function from a Mechanistic General Ecosystem Model
Source: PLoS Biol. 2014 Apr 22;12(4):e1001841. doi: 10.1371/journal.pbio.1001841 (PMC3995663; doi:10.1371/journal.pbio.1001841)
Supplement: Table S4 — Comparison of emergent individual-level properties from the model with observations. The slopes and intercepts of the relationships between predicted properties and body mass compared to empirical data. The probability that the slope and intercept of the predicted relationship were different from those for the empirical data was calculated as the t statistic of linear models fitted to combined model and empirical data for each emergent property using a categorical factor to indicate a model or empirical datum. The probability that the t statistic for the model including the categorical factor indicates the significance of the difference. (DOCX) [file pbio.1001841.s015.docx]

Supplementary Material: Emergent global patterns of ecosystem structure and function from a mechanistic General Ecosystem Model

Running head: A mechanistic general model of global ecosystems

Harfoot, M. B. J.^1,2^*^,†^, Newbold T.^1,2^*, Tittensor, D. P.^1,2,3^*, Emmott, S.^2^, Hutton, J.^1^, Lyutsarev, V. ^2^, Smith, M. J.^2^, Scharlemann, J. P. W.^1,4^, Purves, D. W.^2^

^1^ United Nations Environment Programme World Conservation Monitoring Centre, Cambridge, CB3 0DL, UK

^2^ Microsoft Research Computational Science Laboratory, Cambridge, CB1 2FB, UK

^3^ Dalhousie University, Halifax, NS, B3H 4R2, Canada

^4^ School of Life Sciences, University of Sussex, Falmer, Brighton, BN1 9QG, UK

^*^ These authors contributed equally to this work

^†^ Email: mike.harfoot@unep-wcmc.org

# Table S4. Comparison of emergent individual-level properties from the model with observations

| **Property** | **Cell/Group** | **Empirical slope** | **Model slope** | **Pr(>\|t\|)** | **Significance (<0.05)** | **Empirical intercept (identity space)** | **Model intercept (Identity space)** | **Pr(>\|t\|)** | **Significance (<0.05)** |
| --- | --- | --- | --- | --- | --- | --- | --- | --- | --- |
| Growth | T1 | 0.603 | 0.697 | 5.57E-06 | * | 0.212 | 3.434 | 0.000 | * |
| Growth | T2 | 0.603 | 0.744 | 3.09E-09 | * | 0.212 | 2.676 | 0.000 | * |
| Growth | M1 | 0.603 | 0.775 | 1.17E-16 | * | 0.212 | 6.219 | 0.000 | * |
| Growth | M2 | 0.603 | 0.746 | 1.95E-11 | * | 0.212 | 9.392 | 0.000 | * |
| Growth | All | 0.603 | 0.716 | 5.86E-11 | * | 0.212 | 5.880 | 0.000 | * |
| Growth | Terrestrial | 0.603 | 0.717 | 4.75E-11 | * | 0.212 | 3.086 | 0.000 | * |
| Growth | Marine | 0.603 | 0.768 | 6.12E-22 | * | 0.212 | 7.169 | 0.000 | * |
| Maturity | T1 | 0.172 | 0.244 | 9.04E-09 | * | 222.561 | 30.470 | 0.000 | * |
| Maturity | T2 | 0.172 | 0.160 | 4.30E-01 |  | 222.561 | 95.004 | 0.000 | * |
| Maturity | M1 | 0.172 | 0.119 | 6.12E-08 | * | 222.561 | 64.655 | 0.000 | * |
| Maturity | M2 | 0.172 | 0.132 | 1.10E-04 | * | 222.561 | 53.061 | 0.000 | * |
| Maturity | All | 0.172 | 0.131 | 1.28E-03 | * | 222.561 | 70.406 | 0.000 | * |
| Maturity | Terrestrial | 0.172 | 0.194 | 1.30E-01 |  | 222.561 | 56.536 | 0.000 | * |
| Maturity | Marine | 0.172 | 0.121 | 2.14E-10 | * | 222.561 | 61.635 | 0.000 | * |
| Mortality | T1 | -0.235 | -0.123 | 6.88E-10 | * | 1.146 | 12.550 | 0.000 | * |
| Mortality | T2 | -0.235 | -0.108 | 1.07E-13 | * | 1.146 | 6.420 | 0.000 | * |
| Mortality | M1 | -0.235 | -0.014 | 1.54E-48 | * | 1.146 | 10.485 | 0.000 | * |
| Mortality | M2 | -0.235 | -0.005 | 2.84E-32 | * | 1.146 | 8.756 | 0.000 | * |
| Mortality | All | -0.235 | -0.033 | 1.88E-67 | * | 1.146 | 7.657 | 0.000 | * |
| Mortality | Terrestrial | -0.235 | -0.113 | 5.80E-19 | * | 1.146 | 8.812 | 0.000 | * |
| Mortality | Marine | -0.235 | -0.016 | 3.57E-75 | * | 1.146 | 10.139 | 0.000 | * |
| Lifetime Reproductive Success | T1 | 0.057 | 0.253 | 2.07E-02 | * | 15.408 | 1.082 | 0.001 | * |
| Lifetime Reproductive Success | T2 | 0.057 | 0.285 | 1.24E-02 | * | 15.408 | 2.337 | 0.019 | * |
| Lifetime Reproductive Success | M1 | 0.057 | -0.026 | 9.20E-02 |  | 15.408 | 1.448 | 0.000 | * |
| Lifetime Reproductive Success | M2 | 0.057 | -0.038 | 1.24E-01 |  | 15.408 | 1.955 | 0.001 | * |
| Lifetime Reproductive Success | All | 0.057 | 0.043 | 8.50E-01 |  | 15.408 | 3.295 | 0.013 | * |
| Lifetime Reproductive Success | Terrestrial | 0.057 | 0.277 | 9.21E-03 | * | 15.408 | 1.491 | 0.002 | * |
| Lifetime Reproductive Success | Marine | 0.057 | -0.026 | 9.85E-02 |  | 15.408 | 1.534 | 0.000 | * |

The slopes and intercepts of the relationships between predicted properties and body mass compared to empirical data. The probability that the slope and intercept of the predicted relationship were different from those for the empirical data was calculated as the t-statistic of linear models fitted to combined model and empirical data for each emergent property using a categorical factor to indicate a model or empirical datum. The probability of the t-statistic for the model including the categorical factor indicates the significance of the difference.
