# Supplementary material for: Emergent Global Patterns of Ecosystem Structure and Function from a Mechanistic General Ecosystem Model
Source: PLoS Biol. 2014 Apr 22;12(4):e1001841. doi: 10.1371/journal.pbio.1001841 (PMC3995663; doi:10.1371/journal.pbio.1001841)
Supplement: Table S5 — Summary statistics of empirical community herbivore and primary producer biomasses. Summary statistics, derived from [73], for those ecosystem types most closely representing the ecosystems within the two grid cells shown in Figure 3. PB, primary producer biomass; HB, herbivore biomass. Median herbivore biomass in temperate and tropical grassland ecosystems is 52.3% of that in communities of marine phytoplankton, whereas median primary producer biomass is 57 times larger in the terrestrial compared to the marine ecosystem. (DOCX) [file pbio.1001841.s016.docx]

Supplementary Material: Emergent global patterns of ecosystem structure and function from a mechanistic General Ecosystem Model

Running head: A mechanistic general model of global ecosystems

Harfoot, M. B. J.^1,2^*^,†^, Newbold T.^1,2^*, Tittensor, D. P.^1,2,3^*, Emmott, S.^2^, Hutton, J.^1^, Lyutsarev, V. ^2^, Smith, M. J.^2^, Scharlemann, J. P. W.^1,4^, Purves, D. W.^2^

^1^ United Nations Environment Programme World Conservation Monitoring Centre, Cambridge, CB3 0DL, UK

^2^ Microsoft Research Computational Science Laboratory, Cambridge, CB1 2FB, UK

^3^ Dalhousie University, Halifax, NS, B3H 4R2, Canada

^4^ School of Life Sciences, University of Sussex, Falmer, Brighton, BN1 9QG, UK

^*^ These authors contributed equally to this work

^†^ Email: mike.harfoot@unep-wcmc.org

# Table S5. Summary statistics of empirical community herbivore and primary producer biomasses

| **Community as defined by Cebrian et al.** (Cebrian et al., 2009) | **Biomass** | **Median** |
| --- | --- | --- |
| communities of marine phytoplankton | PB (gC/m2) | 1.72 |
| communities of marine phytoplankton | HB (gC/m2) | 1.40 |
| communities of marine phytoplankton | HB:PB (%) | 52.54 |
| temperate and tropical grasslands | PB (gC/m2) | 98.52 |
| temperate and tropical grasslands | HB (gC/m2) | 0.73 |
| temperate and tropical grasslands | HB:PB (%) | 0.93 |

Summary statistics, derived from (Cebrian et al., 2009), for those ecosystem types most closely representing the ecosystems within the two grid-cells shown in Figure 3 of the manuscript. PB = primary producer biomass, HB = herbivore biomass. Median herbivore biomass in temperate and tropical grassland ecosystems is 52.3% of that in communities of marine phytoplankton, whilst median primary producer biomass is 57 times larger in the terrestrial compared to the marine ecosystem.

# References

Cebrian, J., Shurin, J. B., Borer, E. T., Cardinale, B. J., Ngai, J. T., Smith, M. D., & Fagan, W. F. (2009). Producer nutritional quality controls ecosystem trophic structure. *PLoS ONE*, *4*(3), e4929. doi:10.1371/journal.pone.0004929
