# Supplementary material for: Emergent Global Patterns of Ecosystem Structure and Function from a Mechanistic General Ecosystem Model
Source: PLoS Biol. 2014 Apr 22;12(4):e1001841. doi: 10.1371/journal.pbio.1001841 (PMC3995663; doi:10.1371/journal.pbio.1001841)
Supplement: Table S6 — Comparison of abundance–density slopes predicted by the model with observed slopes. Abundance–density relationships predicted by the model (for cells T1 and M1, Table 4) compared with observations. Empirical abundance–density relationships were derived from Jennings et al. [74]. The reported total biomass versus body mass relationships were converted to abundance versus body mass relationships, by dividing the total biomass in each mass bin by the central mass of that mass bin, which can be approximated as subtracting 1 from the slope of total biomass against body mass. (DOCX) [file pbio.1001841.s017.docx]

Supplementary Material: Emergent global patterns of ecosystem structure and function from a mechanistic General Ecosystem Model

Running head: A mechanistic general model of global ecosystems

Harfoot, M. B. J.^1,2^*^,†^, Newbold T.^1,2^*, Tittensor, D. P.^1,2,3^*, Emmott, S.^2^, Hutton, J.^1^, Lyutsarev, V. ^2^, Smith, M. J.^2^, Scharlemann, J. P. W.^1,4^, Purves, D. W.^2^

^1^ United Nations Environment Programme World Conservation Monitoring Centre, Cambridge, CB3 0DL, UK

^2^ Microsoft Research Computational Science Laboratory, Cambridge, CB1 2FB, UK

^3^ Dalhousie University, Halifax, NS, B3H 4R2, Canada

^4^ School of Life Sciences, University of Sussex, Falmer, Brighton, BN1 9QG, UK

^*^ These authors contributed equally to this work

^†^ Email: mike.harfoot@unep-wcmc.org

# Table S6. Comparison of abundance-density slopes predicted by the model with observed slopes

| **Community** | **Slope** | **Upper 95% Confidence Limit** | **Lower 95% Confidence Limit** | **t value** | **Pr(>\|t\|)** | **Significance (<0.05)** | **Model R-squared** |
| --- | --- | --- | --- | --- | --- | --- | --- |
| Marine Carnivores | -0.02 | 0.05 | -0.08 | 0.67 | 5.02E-01 |  | 5.94E-03 |
| Marine Herbivores | -0.07 | 0.04 | -0.18 | -1.64 | 1.04E-01 |  | 3.43E-02 |
| Marine Omnivores | -0.38 | -0.21 | -0.55 | -0.44 | 6.58E-01 |  | 2.58E-03 |
| Terrestrial Carnivores | -0.01 | 0.04 | -0.05 | -0.06 | 9.55E-01 |  | 4.25E-05 |
| Terrestrial Herbivores | -0.15 | -0.02 | -0.28 | -2.75 | 7.46E-03 | * | 9.04E-02 |
| Terrestrial Omnivores | -0.10 | 0.01 | -0.21 | -1.60 | 1.13E-01 |  | 3.27E-02 |
| All individuals of all species > 64g in the northern North Sea fish community. For the year 2002 | -2.23 | -2.59 | -1.89 | na | na | na | na |
| As above but for 2003 | -2.65 | -2.99 | -2.33 | na | na | na | na |
| As above but for 2004 | -2.48 | -2.73 | -2.23 | na | na | na | na |

Abundance-density relationships predicted by the model (for cells T1 and M1, Table 2) compared with observations. Empirical abundance density relationships were derived from Jennings et al. [1]. The reported total biomass vs. body mass relationships were converted to abundance vs. body mass relationships, by dividing the total biomass in each mass bin by the central mass of that mass bin, which can be approximated as subtracting one from the slope of total biomass against body mass.

# References

1. Jennings S, De Oliveira JAA, Warr KJ (2007) Measurement of body size and abundance in tests of macroecological and food web theory. J Anim Ecol 76: 72–82. doi:10.1111/j.1365-2656.2006.01180.x.
