# Supplementary material for: Emergent Global Patterns of Ecosystem Structure and Function from a Mechanistic General Ecosystem Model
Source: PLoS Biol. 2014 Apr 22;12(4):e1001841. doi: 10.1371/journal.pbio.1001841 (PMC3995663; doi:10.1371/journal.pbio.1001841)
Supplement: Text S1 — Technical and mathematical details of the model. (DOCX) [file pbio.1001841.s018.docx]

Supplementary Material: Emergent global patterns of ecosystem structure and function from a mechanistic General Ecosystem Model

Running head: A mechanistic general model of global ecosystems

Harfoot, M. B. J.^1,2^*^,†^, Newbold T.^1,2^*, Tittensor, D. P.^1,2,3^*, Emmott, S.^2^, Hutton, J.^1^, Lyutsarev, V. ^2^, Smith, M. J.^2^, Scharlemann, J. P. W.^1,4^, Purves, D. W.^2^

^1^ United Nations Environment Programme World Conservation Monitoring Centre, Cambridge, CB3 0DL, UK

^2^ Microsoft Research Computational Science Laboratory, Cambridge, CB1 2FB, UK

^3^ Dalhousie University, Halifax, NS, B3H 4R2, Canada

^4^ School of Life Sciences, University of Sussex, Falmer, Brighton, BN1 9QG, UK

^*^ These authors contributed equally to this work

^†^ Email: mike.harfoot@unep-wcmc.org

# Text S1. Technical and mathematical details of the model

## Autotroph Ecology

The following difference equations (expanded from Equation 1 of the main text) describe the time dynamics of biomass in terrestrial (Equation 1) and marine autotroph stocks (Equation 2). Note that the units of biomass are grams of wet biomass.

| $B_{l,\left( t+\Delta t \right)}=B_{l \left( t \right)}+{\Delta B}_{l}^{Growth}-{\Delta B}_{l}^{Mort}$ |  |
| --- | --- |

| $B_{p,(t+\Delta t)}=B_{p (t)}+{\Delta B}_{p}^{Growth}-{\Delta B}_{p}^{Mort}$ |  |
| --- | --- |

where *B_l_* and *B_p_* are the biomass of leaves and phytoplankton respectively, and Δt is a single time step.

### Marine phytoplankton model

The growth, ${\Delta B}_{p}^{Growth}$, and mortality, ${\Delta B}_{p}^{Mort}$, of phytoplankton over a time step Δ*t* are given by:

| ${\Delta B}_{p}^{Growth}={NPP}_{m}^{M}.\xi.A_{cell}.{\delta t}_{NPP}$ , and |  |
| --- | --- |

| ${\Delta B}_{p}^{Mort}=L_{p,Herbivory}$. |  |
| --- | --- |

where ${NPP}_{m}^{M}$ is an estimate of monthly net primary productivity (NPP) from the Vertically Generalized Production Model [1] using the standard parameterization of the model with surface irradiance corrected for cloudiness, *M* represents a marine cell, *m* is the month, $\xi$ is the conversion factor from carbon to wet matter, $A_{cell}$ is the grid-cell area,${\delta t}_{NPP}$ is a scalar to convert ${NPP}_{m}^{M}$ from its monthly value to the model time step, and $L_{p,Herbivory}$ is the cumulative phytoplankton biomass consumed through herbivory by both herbivores and omnivores during a time step.

### Terrestrial plant model

The leaf growth, ${\Delta B}_{l}^{Growth}$, and mortality, ${\Delta B}_{l}^{Mort}$, rates are given as:

| ${\Delta B}_{l}^{Growth}= G_{ever}+ G_{decid}$ |  |
| --- | --- |

| ${\Delta B}_{l}^{Mort}={{\delta t}_{\mu,l}(\mu_{ever}.B}_{l,\left( t \right)}.f_{ever} +\mu_{decid}B_{l,\left( t \right)}.(1-f_{ever} ))+L_{l,Herbivory}$ |  |
| --- | --- |

where $G_{ever}$ and $G_{decid}$ are the growth rates of evergreen and deciduous leaf stocks respectively, $\mu_{ever}$and $\mu_{decid}$ are the mean leaf mortality rates for evergreen and deciduous stocks respectively,$L_{l,Herbivory}$ is the cumulative leaf biomass consumed through herbivory by both herbivores and omnivores during a time step, and ${\delta t}_{\mu,l}$is a scalar to convert $\mu_{Leaf}$ from its annual rate to the model time step. The equations for the growth rates of evergreen and deciduous leaf stocks are given as:

| $G_{ever}={NPP}_{m}^{T}.A_{cell}.\psi.{\delta t}_{NPP}.\left( 1-f_{struct} \right){.f}_{LeafMort}.f_{ever}$ |  |
| --- | --- |

| $G_{decid}={NPP}_{m}^{T}.A_{cell}.\psi.{\delta t}_{NPP}.\left( 1-f_{struct} \right).f_{LeafMort}.(1-f_{ever})$ |  |
| --- | --- |

where ${NPP}_{m}^{T}$ is monthly terrestrial NPP in kg C m^-2^ month^-^1, *ψ* is a conversion factor from carbon to wet matter, *f_Struct_* is the fractional allocation of primary production to structural tissue, *f_ever_* is the proportion of NPP produced by evergreen leaves at a particular location, and *f_LeafMort_* is the proportion of total mortality that is leaf mortality. The fractional allocation to structural tissue, mean fractional leaf mortality, mean leaf mortality rate, evergreen leaf mortality rate, deciduous leaf mortality rate, and proportion of productivity from evergreen leaves are defined as:

| $f_{Struct}= {\min\left( \frac{f_{struct}^{min}e^{\varphi_{f_{struct}}.{NPP}^{Terr}}}{1+f_{Struct}^{min}.{(e}^{\varphi_{f_{struct}}.{NPP}^{Terr}}-1)}, 0.99 \right).f}_{Struct}^{max}$ |  |
| --- | --- |

| $f_{LeafMort}=\frac{\mu_{Leaf}}{\mu_{Leaf}+\mu_{FineRoot}}$ |  |
| --- | --- |

| $\mu_{Leaf}=$ $e^{f_{ever}.\ln\left( \mu_{ever} \right)+\left( 1-f_{ever} \right).ln(\mu_{decid})}$ |  |
| --- | --- |

| $\mu_{ever}=e^{(m_{e}T_{(t)}^{C}-c_{e})}$ |  |
| --- | --- |

| $\mu_{decid}=e^{-(m_{d}T_{(t)}^{C}+c_{d})}$ |  |
| --- | --- |

| $f_{ever}=a_{f_{ever}}.{F_{frost}}^{2}+b_{f_{ever}}.F_{frost}+ c_{f_{ever}}$ |  |
| --- | --- |

where *μ_FineRoot_* is the rate of mortality of fine roots per year; and $T_{(t)}^{C}$ is monthly average temperature in °C. Mortality rate of fine roots per year is defined as:

| $\mu_{FineRoot}=e^{m_{f}.T_{(t)}^{C}+c_{f}}$ |  |
| --- | --- |

Yearly net primary production was modelled following the Miami model [2]:

| ${NPP}_{y}^{T}=min({NPP}_{T},{NPP}_{P})$ |  |
| --- | --- |

| ${NPP}_{T}=\frac{{NPP}_{max}}{1+e^{c_{P}-m_{P}.T_{(t)}^{C}}}$ |  |
| --- | --- |

| ${NPP}_{P}={NPP}_{max}(1-e^{-\rho.P})$ |  |
| --- | --- |

where *NPP_max­_* _­_is maximum possible net primary production, *c_p_* and *m_p_* are coefficients relating net primary production to temperature, *ρ* relates net primary production to total annual precipitation, *P*. Monthly NPP was then estimated as:

| ${NPP}_{m}^{T}={NPP}_{y}^{T}.\omega_{cell,m}$ |  |
| --- | --- |

where *ω* is an estimate of the contribution that the NPP in month *m* makes to yearly total NPP. This term was introduced to capture seasonal patterns of productivity and was estimated using monthly remote-sensed NPP sourced from the Terra/MODIS net primary productivity data product (see Table S3 for details).

## Heterotroph Ecology

The equations of state for heterotroph cohorts shown below describe the time dynamics for the body mass of an individual (Equation 6), the number of individuals (Equation 7), and the reproductive biomass accumulated by an individual (Equation 8) in cohort *i*.

| $M_{i,(t+\Delta t)}=M_{i(t)}+\Delta M_{i}^{Ass}-\Delta M_{i}^{Metab}-\Delta M_{i}^{ReproAlloc}$ |  |
| --- | --- |

| $N_{i,\left( t+\Delta t \right)}=N_{i(t)}-\Delta N_{i}^{Mort}-\sum_{k}^{N_{C}} (\Delta N_{k,i}^{Pred})$ |  |
| --- | --- |

| $R_{i,(t+\Delta t)}=R_{i,(t)}+\Delta M_{i}^{ReproAlloc}-\Delta R_{i}^{ReproEvent}$ |  |
| --- | --- |

The equations of state for each cohort in the model were iterated forward in time sequentially; i.e. one cohort acted out the ecological processes of eating, metabolism, reproduction, mortality and dispersal, its state variables were updated and then the next cohort 'acted'. As this sequential acting proceeded, each cohort may experience a different ecological environment to the previous. For example, the biomass of an autotroph stock available to be eaten by a cohort acting in the middle of the sequence may be different to that at the beginning of the time step since a previously acting cohort may have removed some biomass.

The ordering of this cohort 'action' was randomised each time step. This was a practical computational solution to the problem of large numbers of interdependent cohorts acting synchronously and will be revisited in future research.

The derivation of each of the terms in the cohort equations of state above are provided in the following sections, arranged according to the ecological process giving rise to that term.

### Eating

Each cohort, *i*, belonged to a functional group, *f*, possessing one of three feeding mode traits: herbivory, carnivory or omnivory. Each functional group assimilated biomass with an efficiency of $\sigma_{f}^{herb}$ from herbivory (herbivores and omnivores only) or $\sigma_{f}^{pred}$ from predation (carnivores and omnivores only). Each functional group engaged in feeding behaviours for a defined fraction, *τ_f_*_,_, of the proportion of each timestep for which environmental conditions enable that functional group to be active, *ς_f(t)_*. The derivation of the proportion of each timestep for which a cohort is active is detailed in the description of activity below.The total biomass assimilated each time step from all stocks, *N_s_*, and all cohorts, *N_C_*, was the sum of the biomass assimilated from herbivory, $\Delta M_{i}^{Ass,Herb}$, and predation, $\Delta M_{i}^{Ass,Pred}$:

| $\Delta M_{i}^{Ass}=\Delta M_{i}^{Ass,Herb}+\Delta M_{i}^{Ass,Pred}$ |  |
| --- | --- |

The biomasses assimilated from herbivory and predation are, respectively:

| $\Delta M_{i}^{Ass,Herb}=\left[ \sum_{k}^{N_{S}} B_{k,\left( t^{*} \right)}.(1-e^{-F_{i,k}.\Delta t_{d}.\tau_{f}.\varsigma_{f,(t)}}) \right].\varepsilon_{f}^{herb}$ |  |
| --- | --- |

and,

| $\Delta M_{i}^{Ass,Pred}=\left[ \sum_{j}^{N_{C}} M_{j,(t^{*})}.N_{j,(t^{*})}.\left( 1-e^{-F_{i,j}.\Delta t_{d}{.\tau}_{f}.\varsigma_{f,(t)}} \right) \right].\varepsilon_{f}^{pred}$ |  |
| --- | --- |

where $B_{k,\left( t^{*} \right)}$, $M_{j,(t^{*})}$ and $N_{j,(t^{*})}$ are the biomasses of autotroph stock *k*, and the individual body mass and abundance of prey cohort *i*. The use of and derivation of exponential differencing within the timestep is described below (see Equations 60 –64). Note that as a result of the sequential, random order of cohort action introduced above, at the time cohort *i* eats, autotroph stock *k* or prey cohort *j* may have been acted upon by other cohorts and may have acted itself, and thus the time index used for referring to stock biomass and prey cohort properties is given as *t^*^* to indicate that these variables may have changed since the start of the time step. *F_i,k_* is the instantaneous fraction of autotroph stock *k* eaten by cohort *i* per day, and *F_i,j_* is the instantaneous fraction of animal cohort *j* eaten by cohort *i* per day. For both *Δt_d_* is the length of the model time step in days. The instantaneous fractions of stocks and cohorts eaten per day were related to the total biomass of autotroph stocks and the densities of animal cohorts following a Holling’s Type III functional response:

| $F_{i,k}= N_{i,(t^{*})}.\frac{K_{i,k}}{1+\sum_{l}^{N_{S}} K_{i,l}.H_{i,l}}.\frac{1}{B_{k,(t^{*})}}$ (where i is herbivorous) |  |
| --- | --- |

| $F_{i,k}=N_{i,(t^{*})}.\frac{K_{i,k}}{1+\sum_{l}^{N_{S}} K_{i,l}.H_{i,l}+\sum_{m}^{N_{C}} K_{i,m}.H_{i,m}}.\frac{1}{B_{k,(t^{*})}}$ (where i is omnivorous) |  |
| --- | --- |

| $F_{i,j}=N_{i,(t^{*})}.\frac{K_{i,j}}{1+\sum_{m}^{N_{C}} K_{i,m}.H_{i,m}}.\frac{1}{N_{j,(t^{*})}}$ (where i is carnivorous) |  |
| --- | --- |

| $F_{i,j}=N_{i,(t^{*})}.\frac{K_{i,j}}{1+\sum_{l}^{N_{S}} K_{i,l}.H_{i,l}+\sum_{m}^{N_{C}} K_{i,m}.H_{i,m}}.\frac{1}{N_{j,(t^{*})}}$ (where i is omnivorous) |  |
| --- | --- |

where *K_i,k_* is the potential biomass, in grams, eaten from autotroph stock *k* by herbivore cohort *i* in a day, and *K_i,j_* is the potential number of individuals of a prey cohort *j* eaten by cohort *i* in a day, both in the absence of handling time constraints. $\sum_{l}^{N_{S}} K_{i,l}.H_{i,l}$ is the total time in days, per day spent searching for food, that would be taken to handle all potential autotroph biomass eaten from each stock *l* among all autotroph stocks *N_s_*. And $\sum_{m}^{N_{C}} K_{i,m}.H_{i,m}$ is the total time in days, per day spent searching for food, that would be taken to handle all potential animal prey items in each cohort *m* among all cohorts *N_C_*; where *H_i,l_* is the time in days for an individual in cohort *i* to handle one gram of autotroph biomass in stock *l* and H_i,m_ is the time in days for an individual in cohort *i* to handle one individual prey item in cohort *m*.

For **herbivory**, the potential rate of biomass eating from stock *k* by cohort *i* was modelled as:

| $K_{i,k}=\alpha_{i,k}.\left( \frac{\phi_{herb,f}.B_{k,\left( t^{*} \right)}}{A_{cell}} \right)^{2}$ |  |
| --- | --- |

where, $\phi_{herb,f}$is the proportion of the current biomass of stock *k* that is experienced by cohort *i*. Note that where $\phi_{herb,f}$ is less than 1.0 then this does not represent a limit on the total amount of stock *k* consumed, since each cohort experiences a separate proportion of the total stock. *α_i,k_* is the effective rate at which an individual herbivore searches its environment in hectares per day, and which is assumed to scale linearly with herbivore body mass:

| $\alpha_{i,k}=\alpha_{0}^{herb}.M_{i,(t)}$ |  |
| --- | --- |

where $\alpha_{0}^{herb}$ is the effective rate per unit body mass at which a herbivore searches its environment in ha day^-1^ gram^-1^.

The handling time for herbivory was assumed to scale according to a power-law function, with exponent *b^herb^*, of the ratio of some reference mass, $M_{ref}^{herb}$ to the herbivore individual body mass:

| $H_{i,k}={h_{0}^{herb}.\left( \frac{M_{ref}^{herb}}{M_{i,(t)}} \right)}^{b^{herb}}$ |  |
| --- | --- |

where *h_0_* is the time in days that it would take a herbivore of body mass equal to the reference mass, to handle one gram of autotroph biomass.

Now that the calculation of herbivory rates on autotroph stocks has been introduced, the loss rate for an autotroph stock *s*, *L_s,herbivory_*, as used in Equations 4 and 6 can now be defined as:

| $L_{s,Herbivory}= \sum_{i=1}^{N_{c}} B_{s,\left( t^{*} \right)}.\left( 1-e^{-F_{i,s}.\Delta t_{d}.\tau_{f}.\varsigma_{f,(t)}} \right)$ |  |
| --- | --- |

For **predation**, the potential number of prey items eaten from cohort *j* by cohort *i* was modelled as:

| $K_{i,j}=\alpha_{i,j}.\left( \frac{N_{j,\left( t^{*} \right)}}{A_{cell}} \right).\Theta_{i,j}$ |  |
| --- | --- |

where *α_i,j_* is the effective rate at which an individual predator searches its environment and successfully kills prey in ha day^-1^, which scales linearly with predator body mass:

| $\alpha_{i,j}=\alpha_{0}^{pred}.M_{i,(t)}.\varpi_{i,j}$ |  |
| --- | --- |

where $\alpha_{0}^{pred}$ is the constant describing the effective rate per unit body mass at which any predator searches its environment in ha day^‑1^ gram^-1^, and *ϖ_i,j_* is the probability of successfully capturing a prey item, which was modelled following [3], assuming that the probability of a predator capturing prey of some proportion of its own body mass follows a normal distribution around some optimum proportion $\theta_{i}^{opt}$ with standard deviation $\sigma_{pred-prey}^{opt}$:

| $\varpi_{i,j}=exp\left[ -\left( \frac{ln\left( \frac{M_{j,\left( t^{*} \right)}}{M_{i,\left( t \right)}} \right)-ln\left( \theta_{i}^{opt} \right)}{\sigma_{pred-prey}^{opt}} \right)^{2} \right]$. |  |
| --- | --- |

For each predator cohort, *i*, the optimal proportional prey mass, $\theta_{i}^{opt}$, is given as:

| $\theta_{i}^{opt}= max\left( \theta_{min,f}^{opt}, N\left( \theta_{f}^{opt},\sigma_{f}^{opt} \right) \right).$ |  |
| --- | --- |

$\Theta_{i,j}$is the cumulative density of organisms with a body mass lying within the same predator specific prey mass bin as *M_j_*. The cumulative densities in each of such bins is calculated as:

| $\Theta_{i,j}= \sum_{k=1}^{N_{c}} \begin{matrix} \frac{N_{k}}{A_{cell}} \\ 0 \end{matrix}\begin{matrix} , where N_{i, k}^{mass} = N_{i, j}^{mass} \\ , otherwise \end{matrix}$, |  |
| --- | --- |

and,

| $N_{i, k}^{mass}= \left\Vert\frac{\log\left( \frac{M_{k}}{M_{i}} \right)- \theta_{i}^{opt}}{0.5 .\sigma_{pred-prey}^{opt}}+ 2N_{\sigma_{pred-prey}^{opt}} \right\Vert$, |  |
| --- | --- |

where the symbol $\left\| \right\|$ means round to the nearsest integer value.

The handling time for predation was assumed to scale as a power-law function, with exponent *b^pred^*, of the reciprocal of predator body mass and linearly with prey body mass, modified from [4]:

| $H_{i,j}= h_{0}^{pred}.\left( \frac{M_{ref}^{pred}}{M_{i,(t)}} \right)^{b^{pred}}.M_{j,(t^{*})}$ |  |
| --- | --- |

where *h_0_* is the time in days that it would take a predator of body mass equal to some reference mass, $M_{ref}^{pred}$, to handle a prey individual of body mass equal to one gram.

### Activity

*ς_f(t)_* is the proportion of the time step that is suitable for a cohort of functional group *f* to be active [5], given by:

| $\varsigma_{f,(t)}= 1 (where f is endotherm)$ |  |
| --- | --- |

| $\varsigma_{f,(t)}= 1-\left( p_{Over,f}+ p_{Below, f} \right) (where f is ectotherm)$ |  |
| --- | --- |

Hence we assumed that endotherms could be active for any of the time step, whilst under certain conditions, the proportion of the timestep that is suitable for ectotherm activity is limited to be some fraction less than 1. This limitation only applies to terrestrial cohorts; marine ectotherms were assumed to have an activity proportion always equal to 1. $p_{Over,f}$ and $p_{Below, f}$refer to the average proportion or each day during the current month, for which the ambient temperature is greater than or less than, respectively, the critical upper, $T_{max, f}^{crit}$, and lower temperature, $T_{min,f}^{crit}$, limits of an ectothermic functional group in the current environment. $T_{max, f}^{crit}$ and $T_{min,f}^{crit}$ apply to terrestrial ectotherm functional groups only and vary with the monthly temperature variability of the grid cell environment [6]:

| $p_{Over, f=}$  $\frac{\left\{ \frac{\pi}{2}-\sin^{-1} \left[ \begin{matrix} 1, \\ -1, \\ {2.\left( T_{max, f}^{crit}- T_{\left( t \right)}^{C} \right)}/{{\Delta T}_{Diurnal}^{C}}, \end{matrix} \begin{matrix} if \left[ T_{max, f}^{crit}-\left( T_{\left( t \right)}^{C}+{{\Delta T}_{Diurnal}^{C}}/2 \right)>0 \right] \\ f\left[ T_{max, f}^{crit}- \left( T_{\left( t \right)}^{C}-{{\Delta T}_{Diurnal}^{C}}/2 \right)<0 \right] \\ otherwise \end{matrix} \right] \right\}}{\pi}$ |  |
| --- | --- |

| $p_{Below, f}= 1- \frac{\left\{ \frac{\pi}{2}-\sin^{-1} \left[ \begin{matrix} 1, \\ -1, \\ {2.(T_{min, f}^{crit}- T_{(t)}^{C})}/{{\Delta T}_{Diurnal}^{C}}, \end{matrix} \begin{matrix} if(T_{min, f}^{crit}- \left( T_{\left( t \right)}^{C}+{{\Delta T}_{Diurnal}^{C}}/2 \right)>0) \\ if(T_{min, f}^{crit}- \left( T_{\left( t \right)}^{C}-{{\Delta T}_{Diurnal}^{C}}/2 \right)<0) \\ otherwise \end{matrix} \right] \right\}}{\pi}$ |  |
| --- | --- |

| $T_{max,f}^{crit}=m_{tol, terrestrial}.\sigma_{T_{Annual}^{C}}+c_{tol,terrestrial}+ T_{Annual}^{C} (where f is terrestrial )$ |  |
| --- | --- |

| $T_{min,f}^{crit}=T_{opt, f}- 4.\left[ \frac{T_{max,f}^{crit}- T_{opt, f}}{12} \right](where f is terrestrial )$ |  |
| --- | --- |

| $T_{opt}= m_{tsm}.\sigma_{T_{Annual}^{C}}+ c_{tsm} + T_{Annual)}^{C} (where f is terrestrial )$ |  |
| --- | --- |

where: ${\Delta T}_{Diurnal}^{C}$ is the monthly mean diurnal temperature range; $T_{Annual}^{C}$ is the annual average temperature in °C; $\sigma_{T_{Annual}^{C}}$is the standard deviation of monthly ambient temperatures across the climatological year in °C.

### Metabolism

Metabolic loss, in grams, was modelled assuming a power-law relationship with body mass and an exponential relationship with temperature following [7]. Whilst an organism is active, metabolism is described by field metabolic rates, whilst when inactive metabolism is described by basal metabolic rates [18]:

| $\Delta M_{i}^{metab}=E_{S}.\left[ \begin{aligned} \left( \varsigma_{f,\left( t \right)}.I_{0,f}^{FMR}.{exp}^{-\left( \frac{E_{A}}{k_{B}.T^{K,body}} \right)}.\left( M_{i\left( t \right)} \right)^{b_{f}^{metab, FMR}} \right) + \\ \left( \left( 1- \varsigma_{f,\left( t \right)} \right).I_{0}^{BMR}.{exp}^{-\left( \frac{E_{A}}{k_{B}{.T}^{K,body}} \right)}.\left( M_{i\left( t \right)} \right)^{b^{metab, BMR}} \right) \end{aligned} \right].{\Delta t}_{d}$ |  |
| --- | --- |

where: $\varsigma_{f,\left( t \right)}$ is the proportion of the current timestep for which environmental conditions are suitable for cohort *i*, belonging to functional group *f*, to be active. Body temperature was assumed to be 310 K for endothermic functional groups and to equal ambient temperature *T^K^* for ectothermic functional groups.

### Reproduction

Whether or not a reproductive event occurred depended on body mass, $M_{i,(t^{*})}$, and reproductive potential mass,$\boxed{R_{i,\left( t^{*} \right)}}$, after the effects of eating, metabolism and allocation to reproductive potential had been taken into account. A reproductive event was assumed to occur when the ratio of total body mass, including reproductive potential, to adult body mass exceeded a critical threshold *β*.

| $if\left( \frac{\boxed{M_{i,\left( t^{*} \right)}}+\boxed{R_{i,\left( t^{*} \right)}}}{M_{i}^{Adult}}>\beta\right)$ |  |
| --- | --- |

In this case, organisms devoted portions of their body mass *M_i_* and reproductive potential mass *R_i_* to producing a number Δ*N_j_* of offspring individuals in a new cohort *j* depending on whether they were iteroparous, and assumed to reproduce several times during their life, or semelparous, and assumed to breed only once: iteroparous organisms were assumed to devote all available reproductive potential mass; and semelparous organisms all available reproductive potential mass as well as a propotion of their current individual body mass, $M_{i,(t^{*})}$.

| $\left. \begin{aligned} \Delta R_{i}^{ReproEvent}=-\boxed{R_{i,\left( t^{*} \right)}} \end{aligned} \right.$ |  |
| --- | --- |

| $\left. \Delta M_{i}^{ReproEvent}= \left\{ \begin{aligned} 0 \left( where i is iteroparous \right), \\ {-M}_{i,\left( t^{*} \right)}.\chi(where i is semelparous) \end{aligned} \right. \right.$ |  |
| --- | --- |

| $\Delta N_{j}^{ReproEvent}=\frac{\Delta R_{i}^{ReproEvent}+\Delta M_{i}^{ReproEvent}}{M_{j}^{Juv}}$ |  |
| --- | --- |

where $M_{j}^{Juv}$ is the juvenile body mass of the new cohort. To allow for some evolution of adult and juvenile body masses between generations, in 5% of reproductive events, juvenile and adult body masses of the newly produced cohort were drawn from a normal distribution, with mean equal to, and with standard deviation that scaled linearly with, parent cohort’s adult and juvenile masses:

| $\boldsymbol{M}_{\boldsymbol{j}}^{\boldsymbol{Juv}}\mathbf{=}\boldsymbol{N}\left( \boldsymbol{M}_{\boldsymbol{i}}^{\boldsymbol{Juv}}\boldsymbol{,}\boldsymbol{\sigma}^{\boldsymbol{J}}\boldsymbol{.}\boldsymbol{M}_{\boldsymbol{i}}^{\boldsymbol{Juv}} \right)$*,* |  |
| --- | --- |

| $M_{j}^{Adult}=N\left( M_{i}^{Adult},\sigma^{A}.M_{i}^{Adult} \right)$. |  |
| --- | --- |

Offspring cohorts always belong to the same functional group as their parent cohorts.

### Non-predation mortality

We modelled three types of mortality in addition to that suffered from predation: background mortality, owing to accidents and disease, which is applied throughout an organism's life history; starvation mortality, owing to metabolic costs exceeding an organism's capacity to sustain itself, again applied throughout an organism's life history; and, senescence mortality, owing to an elevated risk of dying with increasing age, which we apply once organisms have reach reproductive maturity. The total number of individuals dying in a time step from sources of mortality other than predation was calculated as:

| $\Delta N_{i}^{Mort}=N_{i,(t)}.\left( {1-e}^{-\mu_{T}\Delta t_{d}} \right)$ |  |
| --- | --- |

where *μ_T_* is the instantaneous rate of mortality from all non-predation sources of mortality, per day, and $\Delta$*t_d_* is the length of the model time step in days.

| $\mu_{T}=\mu_{bg}+\mu_{se}+\mu_{st}$ |  |
| --- | --- |

where *μ_bg_* is a constant instantaneous rate of background mortality, *μ_se_* is the instantaneous rate of senescence mortality, and *μ_st_* is the instantaneous rate of starvation mortality, all per day. *μ_bg_* is a constant. *μ_se_* was assumed to increase exponentially after a cohort reached maturity:

| $\mu_{se}=\lambda_{se}.{exp}^{\left( \frac{t_{pm}}{t_{bm}} \right)}$ |  |
| --- | --- |

where *λ_se_* is the instantaneous rate of senescence mortality for a cohort at the point of maturity, *t_bm_* is the time that it took a cohort to reach maturity, and *t_pm_* is the time since the cohort reached maturity.

Instantaneous mortality from starvation, $\mu_{st}$, was assumed to increase as a logistic function with decreasing body mass relative to the maximum body mass ever achieved:

| $\mu_{st}=\frac{\lambda_{max}}{1+{exp}^{-\left( \frac{M_{i,\left( t^{*} \right)-\vartheta_{st}M_{i}^{max}}}{\zeta_{st}M_{i}^{max}} \right)}}$ . |  |
| --- | --- |

Where, $M_{i}^{max}$ is the maximum body mass ever achieved by individuals in cohort *i*.

### Dispersal

Cohorts were able to move between grid-cells through the process of dispersal. Three types of dispersal were included in the model, to represent differences between functional groups, life stages and realms. To limit total cohort numbers at a manageable level for computational tractability, we assumed that all individuals in a cohort dispersed together. Although this is an approximation, with sufficient numbers of cohorts and over sufficient time it should converge upon the mean expectation of individual dispersal patterns.

The first type of dispersal was diffusive natal dispersal, i.e. a ‘random walk’ dispersal of juvenile cohorts. We assumed that each cohort had a characteristic dispersal distance *d_i_*, which scaled with its body mass to the exponent, $o_{disp}$:

| $d_{i}=\nu_{disp}.\left( \frac{M_{i,(t^{*})}}{M_{ref}^{disp}} \right)^{o_{disp}}$ |  |
| --- | --- |

Where $\nu_{disp}$ is the dispersal speed of an individual of body mass equal to the dispersal reference mass, $M_{ref}^{disp}$.

Each time step, we assumed that the cohort was spread homogeneously across the grid-cell, and calculated a vector of length *d_i_* and random direction. The whole cohort was then moved along this vector, leading to some proportion of the homogeneous entity being outside of the original grid-cell. The probability of the cohort dispersing from the grid-cell that time step is the proportional area of the cohort that now lay outside the original grid-cell; this is equivalent to the neutral approximation of [8]. The probability of dispersing into any particular neighbouring grid-cell is the relative proportional area of the cohort now lying in that grid-cell. If the vector to be travelled by the cohort would lead to dispersal, but the target grid-cell is in a different realm (land to sea or vice versa), we assumed that the cohort did not disperse that time step (i.e. was bounded by the land/sea interface). All immature organisms, with the exception of planktonic organisms, disperse diffusively; the implicit assumption is that this is equivalent to them searching for new territory.

Adult cohorts, in contrast, undergo behaviourally-mediated dispersal, which could be due to either (1) low densities of individuals (i.e. dispersal to find a mate when encounter rates are very low), or (2) due to starvation (i.e. dispersal away from unsuitable feeding conditions). In the first instance, if area density of individuals is below a mass-related density threshold, given by the criterion,

| $\left. \frac{N_{i,\left( t^{*} \right)}}{A_{cell}}< \frac{\beta_{density}^{responsive}}{M_{i}^{Adult}} \right.$ , |  |
| --- | --- |

they would attempt to undergo dispersal, with the same probability and dispersal distance as outlined in the diffusive dispersal.

In the second instance, we set a starvation threshold,$\beta_{bodymass}^{responsive}$, which was the proportion of body mass lost (relative to adult mass). Therefore, if the following expression is true:

| $\left. \frac{M_{i,(t^{*})}}{M_{i}^{Adult}}< \beta_{bodymass}^{responsive} \right.$. |  |
| --- | --- |

a cohort attempts to disperse every time step while the condition remains true. The chance of a cohort dispersing increased linearly with the level below the starvation threshold. The cohort’s dispersal distance was assumed to be the same mass-related function as for diffusive dispersal.

The final dispersal type, advective-diffusive dispersal, only applies to planktonic organisms in the marine realm. Each cohort disperses based on the two-dimensional advective vector at that timestep and location, with an additional diffusive component of random direction and length following Schlag & North [9]. Velocity data were 50-year monthly averages derived from a data assimilation model, as specified in Table S3. Advective-diffusive dispersal was run multiple times with a shorter process time-step (18 hours) per model time-step (1 month), to prevent cohorts jumping between non-adjacent grid-cells.

## Technical details

### Exponential differencing within time-steps

If we take the change in body mass as a result of herbivory for example, we can demonstrate how if we were to reduce the timestep to an infinitesimally small increment, the exponential differencing approach that we employ would become equal to a continuous time representation that uses the instantaneous rates without any exponentiation.

| $\Delta M_{i}^{Ass,Herb}=\left[ \sum_{k}^{N_{S}} B_{k,\left( t^{*} \right)}.(1-e^{-F_{i,k}.\Delta t_{d}.\tau_{f}.\varsigma_{f,(t)}}) \right].\varepsilon_{f}^{herb}$ |  |
| --- | --- |

We can represent the exponential term in the core of this as a Taylor expansion:

| $e^{-F_{i,k}.\Delta t_{d}.\gamma}= 1+ \left( -F_{i,k}.\Delta t_{d}.\gamma\right)+ \frac{\left( -F_{i,k}.\Delta t_{d}.\gamma\right)^{2}}{2!}+\frac{\left( -F_{i,k}.\Delta t_{d}.\gamma\right)^{3}}{3!}+ \ldots+\frac{\left( -F_{i,k}.\Delta t_{d}.\gamma\right)^{n}}{n!}$, |  |
| --- | --- |

where $\gamma= .\tau_{f}.\varsigma_{f,(t)}$.

As $\Delta t_{d}$ tends towards an infinitesimally small value $dt$, the power terms of the Taylor expansion tend to zero and:

| $e^{-F_{i,k}.\Delta t_{d}.\gamma}=\lim_{\Delta t_{d}\to dt} 1- F_{i,k}.dt.\gamma$ |  |
| --- | --- |

And equation 1 becomes:

| $dM_{i}^{Ass,Herb}=\left[ \sum_{k}^{N_{S}} B_{k,\left( t^{*} \right)}.(1-1+ F_{i,k}.dt.\gamma) \right].\varepsilon_{f}^{herb}$ |  |
| --- | --- |

or

| $\frac{dM_{i}^{Ass,Herb}}{dt}=\left. \sum_{k}^{N_{S}} B_{k,\left( t^{*} \right)}. F_{i,k}.\gamma\right..\varepsilon_{f}^{herb}$ |  |
| --- | --- |

When the timestep is required by practicalities to be longer than *dt*, in our case $\Delta t_{d}$, the exponential differencing formulation approximates the fact that the absolute instantaneous rate of predation mortality for the prey, necessarily decreases over the duration of the timestep as the prey population decreases. The approximation is simply the assumption that the *fractional* loss rate of individuals in the prey population remains constant throughout the time step.

### Cohort merging and extinction

In order to limit the total number of cohorts in the model and ensure the model was computationally tractable, two computational processes of merging and extinction were invoked.

In order to minimise the effects of the **merging** algorithm on the model numerical integration, we merged pairs of cohorts from the same functional group with the shortest relative separation distance in trait space. The number of cohorts to be merged was identified by a threshold for the maximum number of cohorts in a grid-cell. When the number of cohorts exceeded this threshold, a number of pairs of cohorts equal to the number in excess were merged.

The separation distance, $\delta_{i,j}$, between cohort pairs (here cohorts *i* and j) was calculated as a standardised Euclidean distance:

| $\delta_{i,j}= \sqrt{\left( \frac{\left\vert M_{i}^{Adult}-M_{j}^{Adult} \right\vert}{M_{i}^{Adult}} \right)^{2}+ \left( \frac{\left\vert M_{i}^{Juv}-M_{j}^{Juv} \right\vert}{M_{i}^{Juv}} \right)^{2}+ \left( \frac{\left\vert M_{i,(t^{*})}-M_{j,(t^{*})} \right\vert}{M_{i,(t^{*})}} \right)^{2}}$ |  |
| --- | --- |

When a pair of cohorts are merged, for example cohort *j* merging into *i* to yield the merged cohort, *i^m^*, the algorithm indicates (1) that no other merges could be made to the cohort that has been merged from (*j* above) and (2) that the remaining merged cohort (*i^m^* above) could not merge to other cohorts. So the ordering in which cohort pairs were merged influenced the identity of subsequent cohorts merged. We created a list within each functional group of all cohort pair separation distances, then ordered this from shortest to longest and assumed that merging would occur from the beginning of this list. When a merge was assumed to have happened, subsequent cohort pair merges involving a cohort from the assumed merged pair that would violate the above rules were removed from the list. This left an ordered list of permissible merges. The process was repeated for each functional groupand the lists of functional group specific merges combined and sorted again from shortest to longest cohort pair separation distance. The merging algorithm then combined pairs of cohorts, commencing with the first cohort pair in the list and continuing until the required number of cohorts had been merged.

On merging, biomass was conserved by converting the biomass of one cohort into individuals of the other cohort. In the case introduced above of cohort *j* being merged into *i* to yield the merged cohort, *i^m^*, the combined cohort maintains the individual body mass, adult, juvenile and maximum achieved body masses of cohort *i*. The abundance and reproductive potential biomass of the merged cohort, *i^m^* were given respectively by:

| $N_{i^{m},(t)}= N_{i,(t)}+ \left. \frac{N_{j,(t)}. M_{j,(t)}}{M_{i,(t)}} \right.$ *and* |  |
| --- | --- |

| $R_{i^{m},(t)}= R_{i,(t)}+ \left. \frac{N_{j,(t)}. R_{j,(t)}}{N_{i,(t)}} \right.$*.* |  |
| --- | --- |

The direction of the merge was randomised so that in half of merging events involving a cohort *i* and another *j*, the new cohort would be created retaining the properties of cohort *i*, whilst in the other half of events, the new cohort would retain the properties of cohort *j*. This randomisation took place at the same time as the pairwise separation distance was calculated and the direction stored as it had implications for calculation of other permissible merges involving the merging cohorts.

**Extinction** of a cohort meant removal of the cohort from the simulation and occurred if the abundance of a cohort was found to have fallen below the threshold, $\beta^{extinct}$.

### Model initialisation

When the model is initialised, a specified number of cohorts, $\mathbb{N}_{f}$, of each functional group were seeded into each grid-cell.

For each functional group, *f*, the maximum mass,$M_{f}^{max}$, and minimum mass ,$M_{f}^{min}$, of any individual within that group were defined. For a cohort *i* belonging to functional group *f*, these end member values were used to determine the specific but immutable traits of adult mass,$M_{i}^{Adult}$, and juvenile mass,$M_{i}^{Juv}$, that determine the mass range within which the individual mass of each cohort will lie. For example, $M_{i}^{Adult}$is drawn randomly from a uniform distribution in Log_10_ space with an upper bound of $M_{f}^{max}$ and lower bound at $\omega.M_{f}^{min}$:

| $M_{i}^{Adult}={10}^{\left\{ r . \left[ \log_{10} \left( M_{f}^{max} \right)- \log_{10} \left( \omega.M_{f}^{min} \right) \right]+ \log_{10} \left( \omega.M_{f}^{min} \right) \right\}}$. |  |
| --- | --- |

$M_{i}^{Juv}$, is calculated following the draw of $M_{i}^{Adult}$ as follows:

| $M_{i}^{Juv}= \frac{M_{i}^{Adult}}{1+\mathcal{l}\Psi}$, |  |
| --- | --- |

| $\Psi=LogN(\mathcal{a}_{Adult-Juv}+{ϰ\mathcal{b}}_{Adult-Juv}.\ln M_{i}^{Adult}, \sigma_{Adult-Juv})$. |  |
| --- | --- |

$M_{i}^{Juv}$ is calculated iteratively. A new value of $\Psi$ is drawn on each iteration whilst the following criteria were not met: $M_{i}^{Adult}$ must be greater than or equal to $M_{i}^{Juv}$ and $M_{i}^{Juv}$ must be greater than $M_{f}^{min}$. The parameter values in the marine realm are such that, in general, organisms have a larger adult to juvenile body mass ratio. A scaling factor, $ϰ$, is used to increase the expected adult to juvenile mass ratio for functional groups which have a greater adult to juvenile mass ratio than that for other marine organisms.

The initial individual body mass of each cohort, *i*, seeded into each grid-cell of the model was equal to $M_{i}^{Juv}$ and the initial abundance was calculated as follows:

| $N_{i,(t)}=\frac{\mathcal{s.}\left( \frac{\mathcal{c}}{\sum_{f}^{\mathcal{F}} N_{f}} \right)\mathcal{.w.}\mathfrak{b}^{\log_{10} \left( M_{i}^{Juv} \right)}.A_{cell}}{M_{i}^{Juv}}$. |  |
| --- | --- |

Where $N_{f}$is the number of cohorts to be seeded in functional group *f* and $\mathcal{F}$ is the number of functional groups defined for the current grid-cell, such that the total biomass of heterotophs in each grid-cell was reasonable, relatively insensitive to the number of cohorts and the initial abundance of each cohort scaled with the initial body mass of that cohort, approximately following [10].

### Extracting environmental variables

Values for environmental variables for each grid-cell were extracted from the datasets described in Table S3. Where the spatial resolution of the environmental variable dataset was finer than that of the model grid, then the value extracted for a model grid-cell was the mean of the values for each environmental dataset grain falling within the footprint of that model cell. Where the spatial resolution of the environmental variable layer was coarser than the model grid, the value extracted for a model grid-cell was taken as that for the environmental variable grain lying closest to the centre of that model cell.

**References**

1. Behrenfeld M, Falkowski P (1997) Photosynthetic rates derived from satellite-based chlorophyll concentration. Limnol Oceanogr 42: 1–20.

2. Lieth H (1975) Modelling the primary productivity of the world. In: Lieth H, Whittaker RH, editors. Primary productivity of the biosphere. New York, USA: Springer-Verlag. pp. 237–263.

3. Williams RJ, Anandanadesan A, Purves DW (2010) The probabilistic niche model reveals the niche structure and role of body size in a complex food web. PLoS One 5: e12092. doi:10.1371/journal.pone.0012092.

4. Petchey OL, Beckerman AP, Riede JO, Warren PH (2008) Size, foraging, and food web structure. Proc Natl Acad Sci U S A 105: 4191–4196. doi:10.1073/pnas.0710672105.

5. Buckley LB, Hurlbert AH, Jetz W (2012) Broad-scale ecological implications of ectothermy and endothermy in changing environments. Glob Ecol Biogeogr 21: 873–885. doi:10.1111/j.1466-8238.2011.00737.x.

6. Deutsch CA, Tewksbury JJ, Huey RB, Sheldon KS, Ghalambor CK, et al. (2008) Impacts of climate warming on terrestrial ectotherms across latitude. Proc Natl Acad Sci U S A 105: 6668–6672.

7. Brown JH, Gillooly JF, Allen AP, Savage VM, West GB (2004) Toward a metabolic theory of ecology. Ecology 85: 1771–1789.

8. Chisholm RA, Lichstein JW (2009) Linking dispersal, immigration and scale in the neutral theory of biodiversity. Ecol Lett 12: 1385–1393. doi:10.1111/j.1461-0248.2009.01389.x.

9. Schlag ZR, North EW (2012) Lagrangian TRANSport model (LTRANS v.2) User’s guide. Cambridge, MD.

10. Silva M, Downing JA (1995) CRC Handbook of Mammalian Body Masses. Boca Raton, Florida, USA: CRC Press.
