# Supplementary material for: Emergent Global Patterns of Ecosystem Structure and Function from a Mechanistic General Ecosystem Model
Source: PLoS Biol. 2014 Apr 22;12(4):e1001841. doi: 10.1371/journal.pbio.1001841 (PMC3995663; doi:10.1371/journal.pbio.1001841)
Supplement: Text S2 — Model time step effects. (DOCX) [file pbio.1001841.s019.docx]

Supplementary Material: Emergent global patterns of ecosystem structure and function from a mechanistic General Ecosystem Model

Running head: A mechanistic general model of global ecosystems

Harfoot, M. B. J.^1,2^*^,†^, Newbold T.^1,2^*, Tittensor, D. P.^1,2,3^*, Emmott, S.^2^, Hutton, J.^1^, Lyutsarev, V. ^2^, Smith, M. J.^2^, Scharlemann, J. P. W.^1,4^, Purves, D. W.^2^

^1^ United Nations Environment Programme World Conservation Monitoring Centre, Cambridge, CB3 0DL, UK

^2^ Microsoft Research Computational Science Laboratory, Cambridge, CB1 2FB, UK

^3^ Dalhousie University, Halifax, NS, B3H 4R2, Canada

^4^ School of Life Sciences, University of Sussex, Falmer, Brighton, BN1 9QG, UK

^*^ These authors contributed equally to this work

^†^ Email: mike.harfoot@unep-wcmc.org

# Text S2. Model time step effects

It is important to realize that the effects of altering time step on this model should not be viewed as identifying a fatal flaw in the model. For simple models of dynamics defined by differential equations describing states evolving in continuous time, it is highly desirable, and often possible, to find a numerical scheme where the dynamics are insensitive to the method of numerical simulation. But the Madingley Model is dramatically more complex, and less mathematically convenient, than a simple system of differential equations. First, the model includes fundamentally discrete events, that occur when new individuals are created via reproduction. Reproduction introduces new cohorts, which are then simulated according to our functional forms (which are written in continuous time). This adds whole new state variables, and therefore whole new sets of differential equations, regularly throughout the simulation. There is no generic numerical method for dealing with this class of model.

Second, and more importantly, ecological processes do not actually occur in continuous time. Rather, real ecological processes are subject to fundamental breaks in timescale. For example, an hour is not just 1/24 days, because some hours are darker or colder than others due to the diurnal cycle, which in turn triggers behaviours in animals and plants that vary throughout the day in complex ways. Other breaks in timescales are more specific to each organism or class of organism: for example many animals and plants reproduce only once a year, and there is a minimum time over which any one organism can reproduce itself. Because ecological processes do not occur in continuous time, ecological models simulated with a numerical method with a finite time step, are not approximations to a ‘truth’ represented by the dynamics of the model when run in continuous time. Similarly, a simulation with a shorter time-step is not necessarily more ‘true’ than one run with a longer time-step. Rather, any continuous-time equations that appear in ecological models are themselves approximations to an underlying ecology that is discontinuous in time. As such, the equations implicitly assume that they should work appropriately at a certain range of timescales, but not others (e.g. it makes sense to use models with annual time steps that have been parameterized against year-to-year dynamics to simulate processes over scales of multiple years, in steps of one year at a time; but not to use the same model to simulate seasonal dynamics within a year, or hourly dynamics within a day).

As a result, in general we must realistically accept that when dealing with models as complex as GEMs, of which the Madingley Model is intended to be an example, the model formulations and the numerics of the simulations cannot be divided nearly as neatly as is the case for more traditional, simpler ecological models. This is already accepted within the geosciences community for Earth System Models (ESMs). Although in principle, one would like to be able to take the formulation of one model (say, the latest from the UK Met Office, UKMO) and simulate it using the computational and numerical schemes at another institution (say, NCAR), in practise this is not possible for any ESM. Rather, for any given ESM, the formulations and numeric have evolved together, with aspects of each tuned with respect to the other, to give reasonable results. Similarly here, the formulation of the Madingley Model as presented here should be viewed as being contingent on the processes being simulated with a monthly time-step. Apparently, using the same formulations with much shorter time steps can sometimes allow for additional ecological phenomena to occur (exponential growth at time scales much shorter than a month) that are not treated appropriately by the current formulation.

Finally, we would stress that, due to the above, research is needed into numerical methods to simulate GEMs, as mentioned in the main article. Indeed, such research is still ongoing for GCMs, which have a much longer history and are based on physical processes that are much better understood than most ecological processes
